# Supplementary figures and images for: Cryptic genetic variation enhances primate L1 retrotransposon survival by enlarging the functional coiled coil sequence space of ORF1p
Source: PLoS Genet. 2020 Aug 14;16(8):e1008991. doi: 10.1371/journal.pgen.1008991 (PMC7449397; doi:10.1371/journal.pgen.1008991)

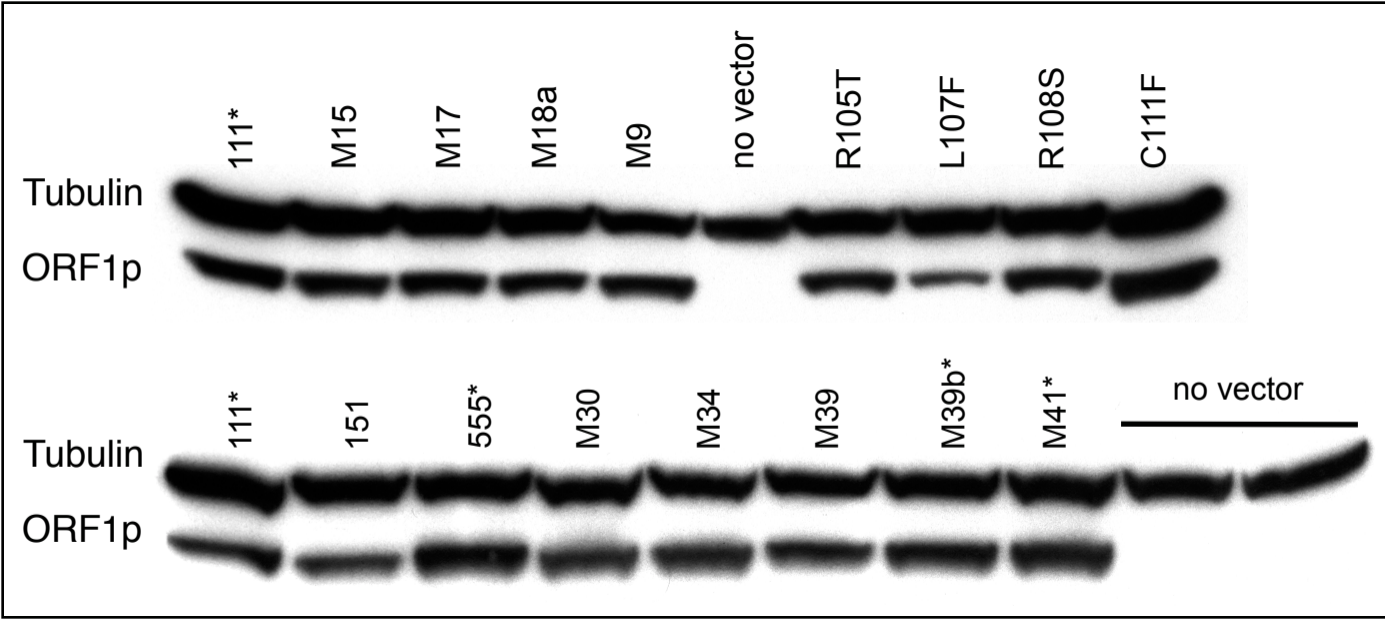

Supplement: S1 Fig — Fifty μg samples of extracts from HeLa cells expressing C-terminally FLAG-tagged ORF1p of the indicated constructs were subject to denaturing gel electrophoresis and Western blotting with anti-Flag and anti-tubulin antibodies as described in the Materials and Methods / Western Blot Analysis. The * indicates ORF1p constructs that were active for retrotransposition. (PDF) [file pgen.1008991.s001.pdf]

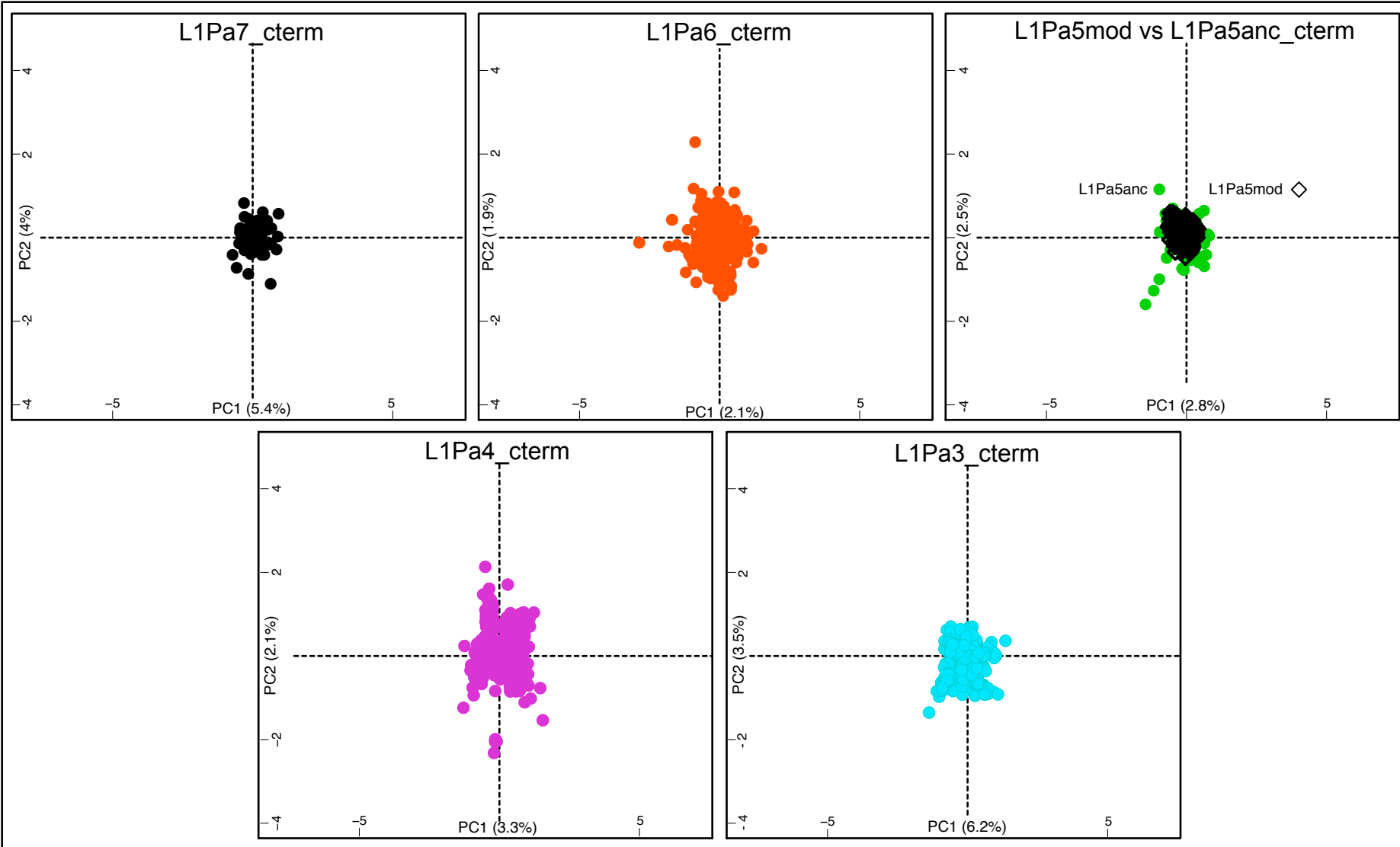

Supplement: S4 Fig — Alignments of the C-terminal half (see Fig 1A) of L1Pa7 –L1Pa3 were analyzed using the Bios2mds R package [50] as described in the Materials and Methods. (PDF) [file pgen.1008991.s004.pdf]

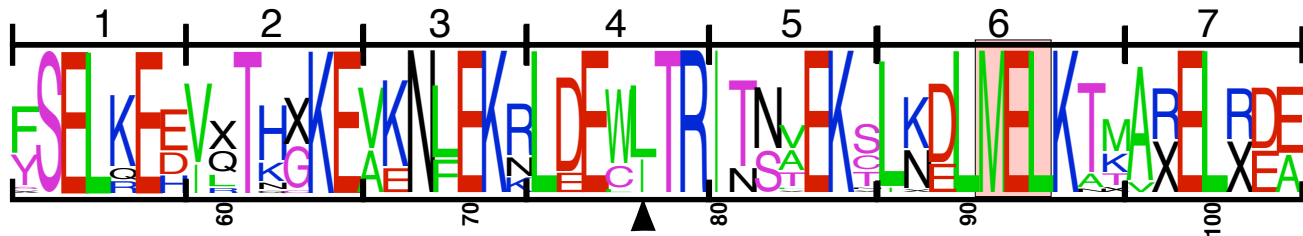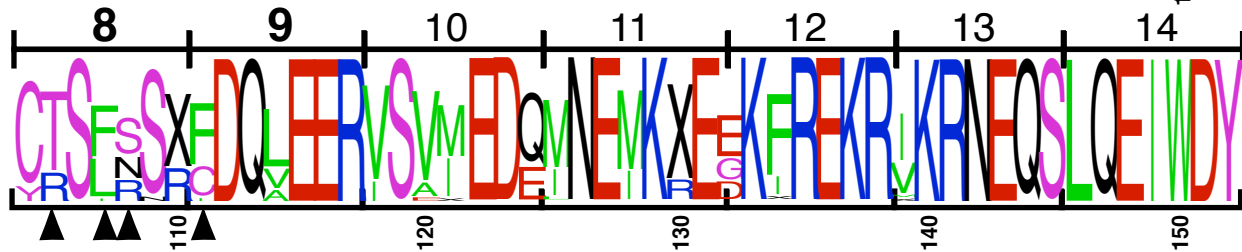

Supplement: S6 Fig — LOGO plot of 50% consensus coiled coil peptide sequences, with CG positions restored, labeled rt in S5 Fig. Arrow heads indicate position of the ancestral amino acids that are negatively epistatic in the modern context. (PDF) [file pgen.1008991.s006.pdf]
