# Supplementary material for: Cryptic genetic variation enhances primate L1 retrotransposon survival by enlarging the functional coiled coil sequence space of ORF1p
Source: PLoS Genet. 2020 Aug 14;16(8):e1008991. doi: 10.1371/journal.pgen.1008991 (PMC7449397; doi:10.1371/journal.pgen.1008991)
Supplement: S2 Fig — These assays are in addition to those shown in Fig 1C carried out as described in Materials and Methods / Retrotransposition assays. To reduce the size of the file, stained cell foci are only shown for the retrotranspositions in panel A. (PDF) [file pgen.1008991.s002.pdf]

A

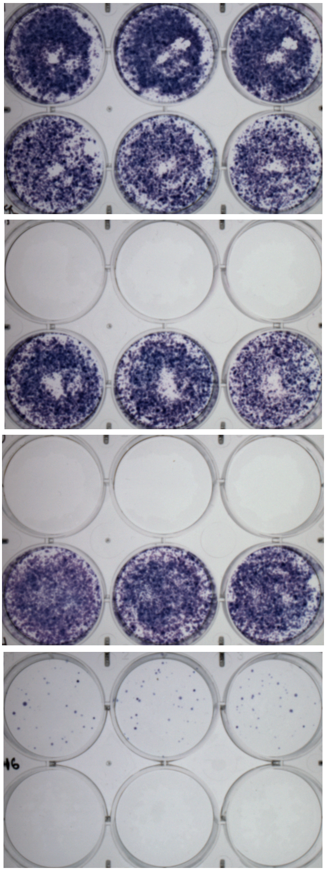

*not presented in paper

B

Wells	ORF1p	Coverage(%)
1 & 2:	111	97 & 97
3 & 4:	151	no foci
5 & 6	m15	0.7 & 0.7
7 & 8:	m17	no foci
9 &10	m18a	0.1 & 0.4
11 & 12:	m9	no foci

C

Wells	ORF1p	Coverage(%)
1 & 2	111	96 & 96
3 & 4	151	no foci
5 & 6	R105T	0.6 & 0.7
7 & 8	L107F	0.4 & 3
9 & 10	R108S	3 & 0.3
11 & 12	C111F	2 & 4

D

Wells	ORF1p	Coverage(%)
1 & 2	111	75 & 73
3 & 4	151	no foci
5 & 6	m14	71 & 66
7	m14	61
8	m15	5
9	m17	0.7
10	m18a	0.8
11	m9	0.4
12	111	67

E

Wells	ORF1p	Coverage(%)
1 & 2	111	73 & 95
3 & 4	111a	83 & 91
5 & 6	151	0.1 & 2.7
7 & 8	151y	0.1 & 0.2
9 & 10	151xa	0 & 1.3
11 & 12	m28c	0.3 & 2
