# Supplementary material for: Cryptic genetic variation enhances primate L1 retrotransposon survival by enlarging the functional coiled coil sequence space of ORF1p
Source: PLoS Genet. 2020 Aug 14;16(8):e1008991. doi: 10.1371/journal.pgen.1008991 (PMC7449397; doi:10.1371/journal.pgen.1008991)
Supplement: S5 Fig — The peptides of the indicated cluster consensus without the CG-affected amino acids (CG-null), indicated by _o, vs. the consensus sequence with the amino acids encoded by CG-affected sites restored, rt. (PDF) [file pgen.1008991.s005.pdf]

```
1.7_o   SS-LKEH VLTQ-KE AKNLEKR LE-LLTR ITSLEKN INDL MEL KNT A-EL-EA YTSINS- IDQ-EER ISEIEDQ LNEIK-E DKIREKR MKRN-QS LQEIWDY
1.7rt   SSELKEH VLTQCKE AKNLEKR LEELLTR ITSLEKN INDL MEL KNT AXELXEA YTSINSX IDQAEER ISEIEDQ LNEIKXE DKIREKR MKRNEQS LQEIWDY

3.7_o   FS-LKEH VLTH-KE AKNLEKR LD-WLTR ITSVEKS LNDL MEL KTT V-EL-EA YTSFNS- FDQ-EER ISVIEDQ INEIK-E DKIREKR VKRN-QS LQEIWDY
3.7rt   FSELKEH VLTHXKE AKNLEKR LDEWLTR ITSVEKS LNDL MEL KTT VXELXEA YTSFNSX FDQAEER ISVIEDQ INEIKXE DKIREKR VKRNEQS LQEIWDY

1b.6_o  FS-LKEH V-TH-KE AKNLEKR LD-WLTR ITSVEKS LNDL MEL KTT V-EL-E- YTSFNS- FDQAEER ISVIEDQ INEIK-E DKIREKR VKRN-QS LQEIWDY
1b.6rt  FSELKEH VLTHXKE AKNLEKR LDEWLTR ITSVEKS LNDL MEL KTT VXELXEA YTSFNSX FDQAEER ISVIEDQ INEIKXE DKIREKR VKRNEQS LQEIWDY

1c.6_o  FS-LKEH V-TH-KE AKNLEKR LD-WLTR INSVEKT LNDL MEL KTT A-EL-D- CTSFNS- FDQVEER ISVIEDQ INEIK-E DKIREKR VKRN-QS LQEIWDY
1c.6rt  FSELKEH VLTHXKE AKNLEKR LDEWLTR INSVEKT LNDL MEL KTT AXELXDA CTSFNSX FDQVEER ISVIEDQ INEIKXE DKIREKR VKRNEQS LQEIWDY

4.6_o   FS-LKEH V-TH-KE AKNLEKR LD-WLTR INSVEKT LNDL MEL KTM A-EL-D- CTSFNS- FDQVEER VSVIEDQ INEIK-E DKXREKR VKRN-QS LQEIWDY
4.6rt   FSELKEH VXTHXKE AKNLEKR LDEWLTR INSVEKT LNDL MEL KTM AXELXDA CTSFNSX FDQVEER VSVIEDQ INEIKXE DKXREKR VKRNEQS LQEIWDY

1a.6_o  FS-LKED V-TH-KE AKNLEKR LD-WLTR INSVEKT LNDL MEL KTM A-EL-D- CTSFNS- FDQVEER VSVIEDQ INEIK-E EKFREKR VKRN-QS LQEIWDY
1a.6rt  FSELKED VXTHXKE AKNLEKR LDEWLTR INSVEKT LNDL MEL KTM AXELXDA CTSFNSX FDQVEER VSVIEDQ INEIKXE EKFREKR VKRNEQS LQEIWDY

3.6_o   FS-LKED V-TH-KE AKNLEKR LD-WLTR INSVEKT LNDL MEL KTM A-EL-D- CTSFSS- FDQVEER VSVIEDQ INEMK-E EKFREKR VKRN-QS LQEIWDY
3.6rt   FSELKED VXTHXKE AKNLEKR LDEWLTR INSVEKT LNDL MEL KTM AXELXDA CTSFSSX FDQVEER VSVIEDQ INEMKXE EKFREKR VKRNEQS LQEIWDY

2.5_o   FS-LKED V-TH-KE AKNLEKR LD-WLTR INS-EKT LNDL MEL KTM A-EL-D- CTSFSS- FDQVEER VSVIEDQ MNEMK-E EKFREKR VKRN-QS LQEIWDY
2.5rt   FSELKED VXTHXKE AKNLEKR LDEWLTR INSVEKT LNDL MEL KTM AXELXDA CTSFSSX FDQVEER VSVIEDQ MNEMKXE EKFREKR VKRNEQS LQEIWDY

3.5_o   FS-LKEE V-TH-KE AKNLEKR LD-WLTR ITSVEKS LNDL MEL KTM A-EL-D- CTSFSS- FDQLEER VSVIEDQ MNEMK-E EKFREKR VKRN-QS LQEIWDY
3.5rt   FSELKEE VXTHXKE AKNLEKR LDEWLTR ITSVEKS LNDL MEL KTM AXELRDE CTSFSSX FDQLEER VSVIEDQ MNEMKXE EKFREKR VKRNEQS LQEIWDY

2.7_o   FS-LKEE VXTH-KE VKNLEKR LD-WLTR ITNXEKX LXDL MEL KTM A-EL-DE CTSFSS- FDQ-EER VSVXEDQ MNEMK-E EKFREKR XKRN-QS LQEIWDY
2.7rt   FSELKEE VXTHXKE VKNLEKR LDEWLTR ITNXEKX LXDL MEL KTM AXELRDE CTSFSSX FDQLEER VSVXEDQ MNEMKXE EKFREKR XKRNEQS LQEIWDY

2.6_o   FS-LKEE V-TH-KE VKNLEKR LD-WLTR ITNAEKS LKDL MEL KTM A-EL-D- CTSFSS- FDQLEER VSVMEDQ MNEMK-E EKFREKR IKRN-QS LQEIWDY
2.6rt   FSELKEE VQTHXKE VKNLEKR LDEWLTR ITNAEKS LKDL MEL KTM AXELRDE CTSFSSR FDQLEER VSVMEDQ MNEMKXE EKFREKR IKRNEQS LQEIWDY

1.5_o   FS-LKEE V-TH-KE VKNLEKR LD-WLTR ITNAEKS LKDL MEL KTM A-EL-D- CTSFSS- FDQLEER VSVMEDQ MNEMK-E EKFREKR IKRN-QS LQEIWDY
1.5rt   FSELKEE VXTHXKE VKNLEKR LDEWLTR ITNAEKS LKDL MEL KTM ARELRDE CTSFSSX FDQLEER VSVMEDQ MNEMKXE EKFREKR IKRNEQS LQEIWDY

3.4_o   FS-LKEE V-THGKE VKNLEKR LD-WLTR ITNAEKS LKDL MEL KT- A-EL-D- CTSFSS- FDQLEER VS-MED- MNEMK-E EKFREKR IKRN-QS LQEIWDY
3.4rt   FSELKEE VXTHGKE VKNLEKR LDEWLTR ITNAEKS LKDL MEL KTM ARELXDE CTSFSSX FDQLEER VSVMEDQ MNEMKXE EKFREKR IKRNEQS LQEIWDY

4.4_o   YS-LKEE V-THGKE VKNLEKK LD-WLTR ITNAEKS LKDL MEL KT- A-EL-D- CTSLSS- FDQLEER VS-MED- MNEMK-E EKFREKR IKRN-QS LQEIWDY
4.4rt   YSELKEE VRTHGKE VKNLEKK LDEWLTR ITNAEKS LKDL MEL KTX ARELXDE CTSLSSX FDQLEER VSVMEDQ MNEMKXE EKFREKR IKRNEQS LQEIWDY

2.3_o   XS-L-EE VXTHGKE VKNLEKK LDEWLTR ITNAEKS LKDL MEL KTM A-EL-DE CTSLSS- FDQLEER VS-MEDQ MNEMK-E EKFREKR IKRN-QS LQEIWDY
2.3rt   XSELKEE VXTHGKE VKNLEKK LDEWLTR ITNAEKS LKDL MEL KTM ARELXDE CTSLSSR FDQLEER VSVMEDQ MNEMKXE EKFREKR IKRNEQS LQEIWDY

2.4_o   YS-LKEE V-TNGKE VKNFEKK LD-WITR ITNAEKS LKDL MEL KT- A-EL-D- CTSLSN- CDQLEER VS-MED- MNEMK-E EKFREKR IKRN-QS LQEIWDY
2.4rt   YSELKEE VRTNGKE VKNFEKK LDEWITR ITNAEKS LKDL MEL KTX ARELRDE CTSLSNX CDQLEER VSVMEDE MNEMKXE EKFREKR IKRNEQS LQEIWDY

1a.4_o  YS-LQEE I-TNGKE VKNFEKK LD-WITR ITNAEKS LKEL MEL KA- A-EL-E- CRSLRS- CDQLEER VS-MED- MNEMK-E GKFREKR IKRN-QS LQEIWDY
1a.4rt  YSELQEE IQTNGKE VKNFEKK LDEWITR ITNAEKS LKEL MEL KAK ARELREE CRSLRSX CDQLEER VSVMEDE MNEMKXE GKFREKR IKRNEQS LQEIWDY

1b.4_o  YS-LQEE I-TKGKE VENFEKN LD-CITR ITNTEKC LKEL MEL KA- A-EL-E- CRSLRS- CDQLEER VS-MED- MNEMK-E GKFREKR IKRN-QS LQEIWDY
1b.4rt  YSELQEE IQTKGKE VENFEKN LDECITR ITNTEKC LKEL MEL KAK ARELREE CRSLRSR CDQLEER VSVMEDE MNEMKRE GKFREKR IKRNEQS LQEIWDY

3.3_o   YS-L-EE IQTKGKE VENFEKN LDECITR ITNTEKC LKEL MEL KAK A-EL-EE CRSLRS- CDQLEER VS-MEDE MNEMK-E GKFREKR IKRN-QS LQEIWDY
3.3rt   YSELQEE IQTKGKE VENFEKN LDECITR ITNTEKC LKEL MEL KAK ARELREE CRSLRSR CDQLEER VSVMEDE MNEMKXE GKFREKR IKRNEQS LQEIWDY

1.3_o   YS-L-ED IQTKGKE VENFEKN LEECITR ITNTEKC LKEL MEL KTK A-EL-EE CRSLRS- CDQLEER VS-MEDE MNEMK-E GKFREKR IKRN-QS LQEIWDY
1.3rt   YSELRED IQTKGKE VENFEKN LEECITR ITNTEKC LKEL MEL KTK ARELREE CRSLRSR CDQLEER VSAMEDE MNEMKRE GKFREKR IKRNEQS LQEIWDY

1.2_o   YSEL-ED IQTKGKE VENFEKN LEECITR ITNTEKC LKEL MEL KTK A-EL-EE CRSLRS- CDQLEER VS-MEDE MNEMK-E GKFREKR IKRNEQS LQEIWDY
1.2rt   YSELRED IQTKGKE VENFEKN LEECITR ITNTEKC LKEL MEL KTK ARELREE CRSLRSR CDQLEER VSAMEDE MNEMKRE GKFREKR IKRNEQS LQEIWDY

1.1_o   YSEL-ED IQTKGKE VENFEKN LEECITR ITNTEKC LKEL MEL KTK A-EL-EE CRSLRS- CDQLEER VSAMEDE MNEMK-E GKFREKR IKRNEQS LQEIWDY
1.1rt   YSELRED IQTKGKE VENFEKN LEECITR ITNTEKC LKEL MEL KTK ARELREE CRSLRSR CDQLEER VSAMEDE MNEMKRE GKFREKR IKRNEQS LQEIWDY
```
