# Supplementary material for: Cryptic genetic variation enhances primate L1 retrotransposon survival by enlarging the functional coiled coil sequence space of ORF1p
Source: PLoS Genet. 2020 Aug 14;16(8):e1008991. doi: 10.1371/journal.pgen.1008991 (PMC7449397; doi:10.1371/journal.pgen.1008991)
Supplement: S7 Fig — Alignment of L1Pa1 coiled coil peptide sequences vs the 50% consensus sequence of the CG-null L1Pa1 coiled coil. (PDF) [file pgen.1008991.s007.pdf]

```
                        60        70        80        90       100       110       120       130       140       150
                --- ----|---- ----|---- ----|---- ----|---- ----|---- ----|---- ----|---- ----|---- ----|---- ----|--
        heptad     1  |  2  |  3  |  4  |  5  |  6 stm  |  7  |  8  |  9  | 10  | 11  | 12  | 13  | 14  |
                ------^------^------^------^------^----+++--^------^------^------^------^------^------^------^------^
                abcdefgabcdefgabcdefgabcdefgabcdefgabcdbcdefgabcdefgabcdefgabcdefgabcdefgabcdefgabcdefgabcdefgabcdefg
1.1_o_50%_cn    YSEL-EDIQTKGKEVENFEKNLEECITRITNTEKCLKELMELKTKA-EL-EECRSLRS-C-QLEERVSAMEDEMNEMK-EGKFREKRIKRNEQSLQEIWDY

1_chr8_2_1      F.....EV*.N..Q.K.L..R.D.WL....SV..S.ND............D..T.FS..F....G...VI..Q......Q.......V....*.....G..
1_chr4_3_2      ......E....D..XX..Q...DK..................N...........R.............V........................I.......
1_chr15_4_3     ......E..I..........S........................P...........................V........................Y..
1_chr6_5_4      .............A.......................................................K...............................
1_chr3_6_5      .........................M...............................R.....K.K...........................T.......
1_chr7_7_6      ...............................R.....................................................................
1_chr3_8_7      ......E........K......DQ...................A...............R........V..A.............................
1_chr5_9_8      .....................................Q...............................................................
1_chr2_10_9     .....................................................................................................
1_chr2_11_10    .....A..........................................................Q....................................
1_chr3_12_11    ..............I....................................Q...................E.............................
1_chrX_13_12    ........L......................R..................................................................*..
1_chr14_14_13   .....................................................................................................
1_chr1_15_14    *....................................................................................................
1_chr18_16_15   .....................................................................................................
1_chr5_17_16    ..K..................................................................................................
1_chr8_18_17    .....................................................................................................
1_chr1_19_18    .............................................................................................T.......
1_chr8_20_19    .......................D................G............................................................
1_chr13_21_20   .....................................................................................................
1_chr4_22_21    ...........D..........................................G................E.............................
1_chr4_23_22    ........................................D..........................................................E.
1_chr5_24_23    .....................................................................................................
1_chr4_25_24    .....K..............X...........................P....................................................
1_chr14_26_25   .......................................................................................V.............
1_chrX_27_26    ...........D.........................................S...............................................
1_chr16_28_27   ............................................Q.......................V...Q..................A.........
1_chr1_29_28    ..........................................N..........................................................
1_chr1_30_29    .....................................................................................................
1_chr18_31_30   ....................................................................................Q................
1_chr13_32_31   ........................Y............................................................................
1_chr2_33_32    ....................................................S......................................K.........
1_chr14_34_33   .....................................................................................................
1_chr8_35_34    .....................................................................................................
1_chr4_36_35    .....................................................................................................
1_chr6_37_36    ....................................................Y.............................V..................
1_chr18_38_37   .....................................................................................................
1_chr9_39_38    .....................................................................................................
1_chr6_40_39    ......N..................V...........................................................................
1_chr8_41_40    .....................................................................................................
1_chr8_42_41    .............................................................P.......................................
1_chr3_43_42    ....................................................................V................................
1_chr4_44_43    .....................................................................................................
1_chr6_45_44    .......................................K.............................................................
1_chrY_46_45    .....................................................................................................
1_chrY_47_46    .......................................T.............................................................
1_chr8_48_47    .....................................................................L...............................
1_chr7_49_48    .....................................................................................................
1_chr1_50_49    .............................................................................R.......................
1_chr3_51_50    ...............................R.....................................................................
1_chr2_52_51    ......................D..............................................................................
1_chr4_53_52    .....................................................................................................
1_chr3_54_53    .....................................................................................................
1_chr5_55_54    .....................................................................................................
1_chr13_56_55   ......................................................................X..............................
1_chr4_57_56    .....................................................................................................
1_chr17_58_57   ..................................Y..................................................................
```

```
    1.1_o_50%_cn YSEL-EDIQTKGKEVENFEKNLEECITRITNTEKCLKELMELKTKA-EL-EECRSLRS-C-QLEERVSAMEDEMNEMK-EGKFREKRIKRNEQSLQEIWDY
                 ------^------^------^------^------^----+++--^------^------^------^------^------^------^------^------^
1_chr18_59_58    .............................I.......................................................................
1_chrX_60_59     ...........................................................W.........................................
1_chr11_61_60    .....................................................................................................
1_chr10_62_61    .....................................................................................................
1_chr14_63_62    .....................................................................................................
1_chr7_64_63     .....................................................................................................
1_chr5_65_64     ..K..................................................................................................
1_chr1_66_65     .................................................................S..............................K....
1_chr6_67_66     .....................................................................................................
1_chr4_68_67     .....................................................................................................
1_chr2_69_68     ........X............................................................................................
1_chr1_70_69     .....................................................................................................
1_chr20_71_70    .....................................................................................................
1_chr5_72_71     ..D..............V...................................................................................
1_chrX_73_72     ..............................................................V......................................
1_chr5_74_73     .....................................................................................................
1_chr5_75_74     .....................................................................................................
1_chr1_76_75     .....................................................................................................
1_chr1_77_76     .....................................................................................................
1_chr15_78_77    .....................................................................................................
1_chr16_79_78    .....................................................................................................
1_chr6_80_79     .....................................................................................................
1_chrX_81_80     ....................................................................V................................
1_chr13_82_81    ....................................................................T................................
1_chr11_83_82    .....................................................................................................
1_chr5_84_83     .............................X.......................................................................
1_chrX_85_84     .....................................................................................................
1_chr1_86_85     .....................................................................................................
1_chr7_87_86     .....................................................................................................
1_chrX_88_87     .....................................................................................................
1_chrX_89_88     .....................................................................................................
1_chr7_90_89     ....................................................................V................................
1_chr11_91_90    .....................................................................................................
1_chr3_92_91     .....................................................................I...............................
1_chr3_93_92     .....................................................................................................
1_chr12_94_93    .....................................................................................................
1_chr4_95_94     ................................................Q....................................................
1_chr1_96_95     .....................................................................................................
1_chr7_97_96     .....................................................................................................
1_chr1_98_97     .............................................T.........................................T.............
1_chr3_99_98     .....................................................................................................
1_chr14_100_99   .....................................................................................................
1_chrX_101_100   .....................................................................................................
1_chr5_102_101   .....................................................................................................
1_chr8_103_102   .............................................................................................T.......
1_chr11_104_103  .....................................................................................................
1_chrX_105_104   .....................................................................................................
1_chr12_106_105  .....................................................................................................
1_chr4_107_106   .....................................................................................................
1_chr10_108_107  .....................................................................................................
1_chr3_109_108   ..................................................................................I..................
1_chr2_110_109   .....................................................................................................
1_chr6_111_110   .....................................................................................................
1_chr2_112_111   .....................................................................................................
1_chr3_113_112   .....................................................................................................
1_chr1_114_113   .....................................................................................................
1_chr2_115_114   .....................................................................................................
1_chr6_116_115   ................................................................*....................................
1_chr11_117_116  .....................................................................................................
1_chr2_118_117   ...............................P.................................................................L...
1_chrX_119_118   .....................................................................................................
1_chr4_120_119   .....................................................................................................
1_chr1_121_120   ..................................................................................V..................
1_chr4_122_121   ..................................................................................V..................
```

```
   1.1_o_50%_cn YSEL-EDIQTKGKEVENFEKNLEECITRITNTEKCLKELMELKTKA-EL-EECRSLRS-C-QLEERVSAMEDEMNEMK-EGKFREKRIKRNEQSLQEIWDY
                ------^------^------^------^------^----+++--^------^------^------^------^------^------^------^------^
1_chr16_123_122 .....................................................................................................
1_chr5_124_123  ....................................................................V................................
1_chr10_125_124 .....................................................................................................
1_chr6_126_125  ..................................................................................................*..
1_chr3_127_126  .....................................................................................................
1_chr18_128_127 .....................................................................................................
1_chr17_129_128 .....................................................................................................
1_chr5_130_129  .....................................................................................................
1_chr3_131_130  ..............I......................................................................................
1_chr14_132_131 .....................................................................................................
1_chr5_133_132  .....................................................................................................
1_chrX_134_133  ..................K.....................................G............................................
1_chr2_135_134  .....................................................................................................
1_chr10_136_135 ....................................................S................................................
1_chr7_137_136  .....................................................................................................
1_chr16_138_137 .....................................................................................................
1_chr15_139_138 .....................................................................................................
1_chr4_140_139  .....................................................................................................
1_chr12_141_140 .....................................................................................................
1_chr14_142_141 .....................................................................................................
1_chr16_143_142 ....................................................................................D................
1_chr20_144_143 .............................................................................................N.......
1_chrX_145_144  .....................................................................................................
1_chr2_146_145  .....................................................................................................
1_chr4_147_146  .....................................................................................................
1_chr11_148_147 ............................................R........................................................
1_chr4_149_148  .....................................................................................................
1_chr18_150_149 ......................................................T..............................................
1_chr18_151_150 .....................................................................................................
1_chr7_152_151  .................................R...................................................................
1_chr11_153_152 .....................................................................................................
1_chr1_154_153  ..................................................................................................R..
1_chr1_155_154  ....................................................................................K................
1_chr11_156_155 .........................................Q...........................................................
1_chr12_157_156 ...........................................................................................A.........
1_chrY_158_157  .....................................................................................................
1_chr15_159_158 .....................................................................................................
1_chrX_160_159  .....................................................................................................
1_chr12_161_160 .....................................................................................................
1_chr4_162_161  .....................................................................................................
1_chrX_163_162  .....................................................................................................
1_chr18_164_163 .....................................................................................................
1_chr2_165_164  .....................................................................................................
1_chr2_166_165  ...................T.................................................................................
1_chr5_167_166  .....................................................................................................
1_chr1_168_167  .....................................................................................................
1_chr10_169_168 .............................................................................N.......................
1_chr12_170_169 .....................................................................................................
1_chr6_171_170  .....................................................................................................
1_chr5_172_171  .....................................................................................................
1_chr2_173_172  .............................................................................................N.....V.
1_chr8_174_173  ..............................X......................................................................
1_chr2_175_174  .....................................................................................................
1_chr3_176_175  .....................................................................................................
1_chr1_177_176  .....................................................................................................
1_chr6_178_177  .....................................................................................................
1_chr4_179_178  .....................................................................................................
1_chrX_180_179  .....................................................................................................
1_chr2_181_180  .....................................................................................................
1_chr3_182_181  .....................................................................................................
1_chr2_183_182  .............................................................R.......................................
1_chr2_184_183  ......................................................................................I........*.....
1_chr22_185_184 ..............I......................................................................................
1_chr11_186_185 .....................................................................................................
```

```
    1.1_o_50%_cn YSEL-EDIQTKGKEVENFEKNLEECITRITNTEKCLKELMELKTKA-EL-EECRSLRS-C-QLEERVSAMEDEMNEMK-EGKFREKRIKRNEQSLQEIWDY
                 ------^------^------^------^------^----+++--^------^------^------^------^------^------^------^------^
 1_chr2_187_186  ...............Q.....................................................................................
 1_chr3_188_187  .....................................................................................................
1_chr10_189_188  .....................................................................................................
1_chr20_190_189  .....................................................................................................
 1_chrX_191_190  .....................................................................................................
 1_chrY_192_191  .............................................V.......................................................
1_chr13_193_192  .....................................................................................................
 1_chr4_194_193  .....................................................................................................
 1_chr5_195_194  .....................................................................................................
 1_chr1_196_195  .....................................................................................................
1_chr10_197_196  .....................................................................................................
 1_chr4_198_197  .....................................................................................................
1_chr13_199_198  .....................................................................................................
 1_chr9_200_199  .....................................................................................................
 1_chr4_201_200  .....................................................................................................
 1_chr4_202_201  ..............A.................................................G....................................
 1_chr5_203_202  ..................................S..................................................................
 1_chr2_204_203  ................................................................V....................................
 1_chr7_205_204  .....DN..............................................................................................
 1_chrX_206_205  ...............Q.....................................................................................
 1_chr4_207_206  .....................................................................................................
1_chr11_208_207  .............................................................................................N.......
 1_chr7_209_208  ................................................................................E............N.......
 1_chr6_210_209  ................X....................................................................................
 1_chr1_211_210  ....................X................................................................................
1_chr20_212_211  ....................................................................T................................
 1_chr9_213_212  .....................................................................................................
1_chr13_214_213  .....................................................................................................
 1_chrX_215_214  .....................................................................................................
 1_chr4_216_215  .....................................................................................................
 1_chr2_217_216  .........................................P...........................................................
 1_chr1_218_217  ....................................................................................G................
1_chr16_219_218  .....................................................................................................
 1_chr2_220_219  .....................................................................................................
1_chr20_221_220  .....................................................................................................
1_chr15_222_221  .....................................................................................................
1_chr22_223_222  .............................................T.......................................................
 1_chr4_224_223  .....................................................................................................
1_chr12_225_224  .....................................................................................................
 1_chrX_226_225  ....................................................................................D................
 1_chr3_227_226  .................................................................K...................................
 1_chr7_228_227  .....................................................................................................
 1_chr1_229_228  .....................................................................................................
 1_chr1_230_229  .....................................................................................................
 1_chr6_231_230  .....................................................................................................
 1_chr5_232_231  .....................................................................................................
 1_chr9_233_232  .....................................................................................................
1_chr17_234_233  .....................................................................................................
 1_chr5_235_234  .....................................................................................................
 1_chr9_236_235  .....................................................................................................
 1_chr7_237_236  .....................................................................................................
 1_chr6_238_237  ..........E..........................................................................................
 1_chr9_239_238  .....................................................................................................
 1_chr1_240_239  .....................................................................................................
 1_chrX_241_240  ....................................................................P............................M...
 1_chr4_242_241  .....................................................................................................
1_chr11_243_242  .........N...........................................................................................
 1_chr5_244_243  .........................V...........................................................................
 1_chr1_245_244  .....................................................................................................
 1_chr3_246_245  .....................................................................................................
 1_chr3_247_246  .....................................................................................................
 1_chr3_248_247  .....................................................................................................
 1_chr6_249_248  .....................................................................................................
 1_chr8_250_249  .....................................................................................................
```

```
   1.1_o_50%_cn YSEL-EDIQTKGKEVENFEKNLEECITRITNTEKCLKELMELKTKA-EL-EECRSLRS-C-QLEERVSAMEDEMNEMK-EGKFREKRIKRNEQSLQEIWDY
                ------^------^------^------^------^----+++--^------^------^------^------^------^------^------^------^
1_chr1_251_250  .....................................................................................................
1_chrX_252_251  .....................................................................................................
1_chr4_253_252  .....................................................................................................
1_chr5_254_253  .....................................................................................................
1_chr9_255_254  ....................................................................T................................
1_chr11_256_255 .....................................................................................................
1_chr9_257_256  .....................................................................................................
1_chr11_258_257 .....................................................................................................
1_chr6_259_258  .....................................................................................................
1_chr15_260_259 .....................................................................................................
1_chr7_261_260  .....................................................................................................
1_chrX_262_261  .....................................................................................................
1_chr2_263_262  .....................................................................................................
1_chr8_264_263  .....................................................................................................
1_chr4_265_264  .....................................................................................................
1_chr11_266_265 .....................................................................................................
1_chr16_267_266 .............................S.......................................................................
1_chr10_268_267 .....................................................................................................
1_chr16_269_268 .....................................................................................................
1_chr4_270_269  .....................................................................................................
1_chr6_271_270  .....................................................................................................
1_chr13_272_271 .....................................................................................................
1_chr1_273_272  .....................................................................................................
1_chr6_274_273  .....................................................................................................
1_chr15_275_274 .....................................................................................................
1_chr8_276_275  .....................................................................................................
1_chr10_277_276 .....................................................................................................
1_chr14_278_277 .....................................................................................................
1_chr17_279_278 .....................................................................................................
1_chr4_280_279  .....................................................................................................
1_chr4_281_280  .....................................................................................................
1_chr5_282_281  .....................................................................................................
1_chr6_283_282  .....................................................................................................
1_chr7_284_283  .....................................................................................................
1_chr7_285_284  .....................................................................................................
1_chr8_286_285  .....................................................................................................
1_chr4_287_286  ......E..............................................................................................
1_chr1_288_287  .....................................................................................................
1_chr11_289_288 .....................................................................................................
1_chr12_290_289 .....................................................................................................
1_chr3_291_290  .....................................................................................................
1_chr4_292_291  .....................................................................................................
1_chr5_293_292  .....................................................................................................
1_chr7_294_293  .....................................................................................................
1_chr7_295_294  .....................................................................................................
1_chrX_296_295  .....................................................................................................
```
