# Supplementary material for: Cryptic genetic variation enhances primate L1 retrotransposon survival by enlarging the functional coiled coil sequence space of ORF1p
Source: PLoS Genet. 2020 Aug 14;16(8):e1008991. doi: 10.1371/journal.pgen.1008991 (PMC7449397; doi:10.1371/journal.pgen.1008991)
Supplement: S8 Fig — Alignment of L1Pa2 coiled coil peptide sequences vs the 50% consensus sequence of the CG-null L1Pa2 coiled coil. (PDF) [file pgen.1008991.s008.pdf]

```
                         60        70        80        90       100       110       120       130       140       150
                 --- ----|---- ----|---- ----|---- ----|---- ----|---- ----|---- ----|---- ----|---- ----|---- ----|--
         heptad     1  |   2  |   3  |   4  |   5  |  6 stm  |   7  |   8  |   9  |  10  |  11  |  12  |  13  |  14  |
                 ------^------^------^------^------^----+++--^------^------^------^------^------^------^------^------^
                 abcdefgabcdefgabcdefgabcdefgabcdefgabcdbcdefgabcdefgabcdefgabcdefgabcdefgabcdefgabcdefgabcdefgabcdefg
1.2_o_50%_cn     YSEL-EDIQTKGKEVENFEKNLEECITRITNTEKCLKELMELKTKA-EL-EECRSLRS-C-QLEERVS-MEDEMNEMK-EGKFREKRIKRNEQSLQEIWDY

2a_chr19_2_1     F......V*.HH..AK.L..R.DKWL...NSV..S.ND......I.....DA.T.FN..F.KV......I..Q.......E......L.............
2a_chrX_3_2      H.....EV*.N....KH.X.KTDNSV.....A..S..D.T.V..A.....DK.L..ST.*.......X............E..........K.........
2a_chr11_4_3     ...P.KEVR.H....K.L..K.D.WL.....A..S..D......TT....D..T..S..F..........K.Q.......K.C....V...KK........
2a_chr16_5_4     ..K...EVR.N....K.L..K.D*WL.....E..S..D.....AM.....D..T..S...............K.......D........I......K...C
2a_chr6_6_5      ......E.*.N....K.LX.K.D.WL.....A..S..D......V...I.D..T..S.......A...............E.....G..S....FP.....
2a_chrY_7_6      .....K...............S...T..................................................K...........E............
2a_chrX_8_7      .C....E........K..G...D.R.P................A.....................T..............R....................
2a_chr2_9_8      F......VR.QR..AK.C..R.DKWL....SV..S.ND..D...ME....DA.T.FS..F...KX....T..Q.......E......V............H
2a_chrY_10_9     .P....E........K.L....DK...G....Q.......D..A.V.............F.......................G...V.....N.......
2a_chr3_11_10    *.........E............D................................K..........T.V.....A.........................
2a_chrX_12_11    ......N...........X......V.........S......................................................ID......SE.
2a_chr5_13_12    ........*...R..............................................*.........................................
2a_chr8_14_13    ..............L.........R............................................................................
2a_chr2_15_14    .....................................*...............................................................
2a_chr15_16_15   ....................H........................V........................D..........N...................
2a_chr6_17_16    .....................................................................................................
2a_chr5_18_17    .....................................................................................................
2a_chr9_19_18    ...............................................................................D.....................
2a_chr9_20_19    ................................D..........S...........................................M.............
2a_chr6_21_20    .......................................................................................T.............
2a_chrX_22_21    .....................................................................................................
2a_chr3_23_22    .P...K.......................................................................................R.......
2a_chr6_24_23    ....................K......TV............................................R...........................
2a_chr5_25_24    ...................................................................................K............K....
2a_chr3_26_25    ...................................................................................................GC
2a_chr2_27_26    .......................................................................N.......D.....................
2a_chr13_28_27   ........................................................................G...............Q............
2a_chr14_29_28   ................................................................D...........V........................
2a_chr9_30_29    .......S.............................................................................................
2a_chr11_31_30   .C...................................................................................................
2a_chr2_32_31    ......................................................R.............................G................
2a_chr6_33_32    .....................V...............................................................................
2a_chr10_34_33   ......................D.R...........................................................Q................
2a_chr7_35_34    .....................*..........................................................X....................
2a_chr4_36_35    ............................R...D....................................................................
2a_chr8_37_36    ..................................................................................V..................
2a_chr4_38_37    ................S..................*....................................................Q.....P..T...
2a_chr3_39_38    ..................X..............................................S...................................
2a_chr9_40_39    .....................................D...............................................................
2a_chr4_41_40    .............K........A..............................................................................
2a_chr3_42_41    ..........................S..........................................................................
2a_chr7_43_42    ...................Q....F....S.......................................................................
2a_chr13_44_43   ............................R........................................................................
2a_chr2_45_44    ........................................................................................*............
2a_chr2_46_45    .........................................................I...........................................
2a_chr8_47_46    ...........................................N.........................................................
2a_chr7_48_47    ........L........L...................................................................................
2a_chr6_49_48    ................................A......................H..........................V..................
2a_chr2_50_49    .........................T.................................R.........................................
2a_chr3_51_50    ................T....................................................................................
2a_chr3_52_51    ...................................................................A...........................K.....
2a_chr18_53_52   ..................................................................................................R..
2a_chr16_54_53   .A.............................................................................................*...E.
2a_chr4_55_54    ........................................................................K...............E............
2a_chr4_56_55    .......T................S..................................G.........................................
2a_chr7_57_56    .....................................................................................................
2a_chr12_58_57   ............................................................................................E........
```

```
   1.2_o_50%_cn    YSEL-EDIQTKGKEVENFEKNLEECITRITNTEKCLKELMELKTKA-EL-EECRSLRS-C-QLEERVS-MEDEMNEMK-EGKFREKRIKRNEQSLQEIWDY
                   ------^------^------^------^------^----+++--^------^------^------^------^------^------^------^------^
2a_chr8_59_58      .......................................................................................R.............
2a_chr6_60_59      ..........................................R..........................................................
2a_chr7_61_60      ................................................................X....................................
2a_chr9_62_61      .....................................................................................................
2a_chr10_63_62     .....................................................................................................
2a_chr4_64_63      ...............................................................K.....................................
2a_chr2_65_64      .....................................................................................................
2a_chr5_66_65      ....................K................................................................................
2a_chr13_67_66     .......L..........................................................................................*..
2a_chr17_68_67     ........................R...................................................V........................
2a_chr14_69_68     .........A...........................................................................................
2a_chr12_70_69     .......T.............................................................................................
2a_chr8_71_70      .............................................................................................N.......
2a_chr12_72_71     ......................D...........................................................S..................
2a_chr6_73_72      ................................D................................................................V...
2a_chrX_74_73      ....................K.................Q..............................................................
2a_chr7_75_74      .....G.........................................K.....................................................
2a_chr2_76_75      .C.R...............N..........................................Q.............................K........
2a_chr1_77_76      ........................G.......G....................................R...............................
2a_chr4_78_77      ............................K.....................K............QGK...................................
2a_chr20_79_78     ......................D.W.......................................V..........................K.........
2a_chr9_80_79      ........X.........................................K.............................E....................
2a_chr2_81_80      .........................M...........................................................................
2a_chr4_82_81      .................Y.........G.........................................................................
2a_chr4_83_82      .................................Q....................................................*..............
2a_chr5_84_83      .............K....................................................................V..................
2a_chr17_85_84     .......M............H...........................................................................D....
2a_chr4_86_85      .....................................................................................................
2a_chr5_87_86      ...........................................................*..........V..............................
2a_chr14_88_87     ................................G.................................................Y..................
2a_chr14_89_88     .....................................................................................................
2a_chr12_90_89     .....................................................................I........................F......
2a_chr14_91_90     ..............................S.............E........................................................
2a_chr11_92_91     .....................................................................................................
2a_chr12_93_92     .................................................................T..........T........................
2a_chr3_94_93      ..............I.......................V...............................................*..............
2a_chr10_95_94     N..............................I.....................................................................
2a_chr8_96_95      .......................................................................................T.............
2a_chrX_97_96      .....................................................................................................
2a_chr9_98_97      ...............D............................E......................P.................................
2a_chr4_99_98      .......................................T.............................................................
2a_chr6_100_99     ........*.........................R..L.K.............................................................
2a_chr6_101_100    ...............................................................................D............E........
2a_chr5_102_101    .......T.................K...........................................................................
2a_chr3_103_102    .....................................................................................................
2a_chr2_104_103    ..................................................................................................G..
2a_chr5_105_104    ........................................................................D................T...........
2a_chrX_106_105    ...................................................................................................G.
2a_chr4_107_106    ......................*.................K............................................................
2a_chr3_108_107    .....................................................................................................
2a_chr11_109_108   ...........C...................................D.......V.............................................
2a_chr1_110_109    ..................................................................I..................................
2a_chr3_111_110    .....................................................................................................
2a_chr4_112_111    ...........................................................................................Q.R.......
2a_chr10_113_112   ...........................K......................................L.............R....................
2a_chr19_114_113   .................................E....Q........................Q.GX...K...........................C..
2a_chrY_115_114    ..A............................P....N.............................................S............R.....
2a_chr6_116_115    ....................K................................................................................
2a_chr12_117_116   ......E......A........D......S.............A....Q.....G.........................E....................
2a_chr11_118_117   ....................K..............................................................K.................
2a_chrX_119_118    ...................................*..............................A........................Q.........
2a_chr11_120_119   ....................S......................I....P.D......X...........................................
2a_chr10_121_120   ......X...........Q.........L..I.....................G.....................*.........................
2a_chrY_122_121    .........N......................................................................R.SI.................
```

```
   1.2_o_50%_cn  YSEL-EDIQTKGKEVENFEKNLEECITRITNTEKCLKELMELKTKA-EL-EECRSLRS-C-QLEERVS-MEDEMNEMK-EGKFREKRIKRNEQSLQEIWDY
                 ------^------^------^------^------^----+++--^------^------^------^------^------^------^------^------^
2a_chr17_123_122 .......................D.....I.......M..........V..........R................T........E...............
2a_chr8_124_123  ....................F.................V..P..*........................................................
2a_chr8_125_124  ......E...N....KS...K...W......A..S..D.....I......D....HS.......................E.....S....N....D....
2a_chr8_126_125  ..........................I...........V..................G.............N.............................
2a_chr8_127_126  ...P.........X.......................................................................................
2a_chrX_128_127  .............D...............N.....................*.................................................
2a_chr11_129_128 ....................K....................M.........G..NF..............A...........S..................
2a_chr11_130_129 ....................K...........Q........M.........G..N...............A...........S........K.........
2a_chr11_131_130 *.K..KEVR.H....K.L..K.DKWL.....A..S..D............D..T..S..F....................E....................
2a_chr7_132_131  ................................K............V.K...................P.....................K....I......
2a_chr8_133_132  ..Q.............................KN......................W.......D.................S...............R..
2a_chr7_134_133  ......................................................................................L..............
2a_chr6_135_134  ...............................................................K..........................K..........
2a_chr2_136_135  ..................X..................................................I...............................
2a_chrY_137_136  ...........................................................G..V......................................
2a_chr7_138_137  ...................Q...........................D..................I...............S..................
2a_chr8_139_138  .....................S............................................................Y..QT....K.........
2a_chr6_140_139  .............Q..................K....................................................................
2a_chr11_141_140 ......E..............................................................................................
2a_chr5_142_141  ......A......................................................................................G.......
2a_chrY_143_142  .....................V.............................................................G.................
2a_chrY_144_143  .....................V.............................................................G.................
2a_chrY_145_144  .....................V.............................................................G.................
2a_chrY_146_145  .....................V.............................................................G.................
2a_chr11_147_146 .....................................................................................................
2a_chr6_148_147  .....*...................................M..........................................K..T.............
2a_chr6_149_148  ......ET...........T..D....................A.T......................................D................
2a_chr2_150_149  .................................R.............D...............................*.....................
2a_chr6_151_150  .....V.....................KM..I...................Q.K.....R.........................................
2a_chr3_152_151  ..................L.......I........................................L.................................
2a_chr5_153_152  .........S..........................................F..................E.............................
2a_chr2_154_153  ..................................................................................................R.S
2a_chr19_155_154 .....................S.....P.........................................................................
2a_chr5_156_155  .........N....................I............................................G.........................
2a_chr5_157_156  .............................................................H.......................................
2a_chr2_158_157  ............E............................................G..........................G.AN.X...........
2a_chr20_159_158 ...........D.............................P........................................S..................
2a_chr11_160_159 ....................................X..................H...R.................T..V....................
2a_chr1_161_160  ...........................................................................................Q.........
2a_chr7_162_161  .....K...................................................R..................I........................
2a_chr1_163_162  .............K.............................................................X.........................
2a_chr1_164_163  .............................X.......D...............................................R......P........
2a_chr10_165_164 ......................................................................................G..............
2a_chr2_166_165  .........................V.................N............K..............E.............................
2a_chr2_167_166  ....................T.................................................................G..K.Q.........
2a_chr8_168_167  ..................X........................................................G.........................
2a_chr6_169_168  ........................................D............................................................
2a_chr5_170_169  .....................V.............................................................K.................
2a_chr5_171_170  .........S..............................................S....................E....................*..
2a_chr11_172_171 ..........................S.....*........................R................KK...............K.........
2a_chrX_173_172  ..................................................................................S..................
2a_chr7_174_173  ..................A..........................................................R.......................
2a_chr1_175_174  ......G..............*............................................X...............S..E...............
2a_chr13_176_175 ........................G..............T.............................................................
2a_chr3_177_176  ......................................................................................*.............C
2a_chr7_178_177  ................................................................D....................................
2a_chr3_179_178  .........................................................................................T...........
2a_chr6_180_179  ......N....S..........K..M.......................................................N....I..............
2a_chr8_181_180  .........N...........................................................I...............................
2a_chr3_182_181  .....................................................................................................
2a_chr6_183_182  ..............................................................Q......................................
2a_chr7_184_183  ...............................................................................D.....................
2a_chr11_185_184 ..A..............S...................................................................................
2a_chrY_186_185  ....................G.............................................................L.............A....
```

```
  1.2_o_50%_cn    YSEL-EDIQTKGKEVENFEKNLEECITRITNTEKCLKELMELKTKA-EL-EECRSLRS-C-QLEERVS-MEDEMNEMK-EGKFREKRIKRNEQSLQEIWDY
                  ------^------^------^------^------^----+++--^------^------^------^------^------^------^------^------^
2a_chrY_187_186   ....................G.............................................................L.............A....
2a_chr12_188_187  X...................K....V...........................................................................
2a_chrX_189_188   C..................N.......G.S.............................Y............K...........................H
2a_chr8_190_189   ....................................................................................................C
2a_chr5_191_190   .......................D.M...........................................................................
2a_chrX_192_191   ........................R............................................................................
2a_chr6_193_192   .....................................................................................................
2a_chr2_194_193   ........................................V................N....................................R......
2a_chr18_195_194  ....................H............*..............................................................A....
2a_chr5_196_195   .............................S..GN................................................................*..
2a_chr4_197_196   ..........................S..S...........................................T...........................
2a_chr10_198_197  .....Q...........................................................K...................................
2a_chrX_199_198   C....................................................................................................
2a_chr8_200_199   ...........................................................................D......S..................
2a_chr5_201_200   .................................................................................................T.Y.
2a_chr9_202_201   N.......................Y......I.R...................................................................
2a_chr4_203_202   ...............................R................................G.I..........................N.......
2a_chr1_204_203   ............................V........................................................................
2a_chr4_205_204   ....................S...........................................K....................................
2a_chr4_206_205   ..................X..................................................................................
2a_chr1_207_206   ......................D....................................*.........................................
2a_chr5_208_207   ....................................................Y................................................
2a_chr1_209_208   ......G.............D...............................R...................................Q...*........
2a_chr1_210_209   ...........D.............M....Y.........................................................Q............
2a_chr4_211_210   ...........D.............................Q.......................S...................................
2a_chr6_212_211   ...............................................K.................T...T...............................
2a_chr18_213_212  ........................................................K........S.........................D.........
2a_chr5_214_213   .................X.......V...........K............D............K.......E.............................
2a_chr16_215_214  ..................................................................................S..................
2a_chr4_216_215   .......V..........X.....................Q............................................................
2a_chr1_217_216   ............................V................................L.......................................
2a_chr18_218_217  ....................................................S.........M...................L..................
2a_chr4_219_218   .F...............................................................K...................................
2a_chr11_220_219  ......................................P.....N........................................................
2a_chr10_221_220  ..D..................................................................................................
2a_chr3_222_221   ................S.Q..F..*L....................P..............H...S...................................
2a_chr2_223_222   ........E.............................V.........................K................X..G................
2a_chr1_224_223   ..........E.......K..............R.......................T.........T.........................N...M...
2a_chr10_225_224  ...............................................................................D.....................
2a_chr1_226_225   .T.........................................A........................................D................
2a_chr11_227_226  ................................................................V..........................K.........
2a_chr11_228_227  ...........................................A........R........................................N.......
2a_chr12_229_228  S...................H..................K.............................................................
2a_chrX_230_229   ...............................R.....................................................................
2a_chr4_231_230   ...V.........*.......................................................................................
2a_chrX_232_231   .....................................................................................................
2a_chr18_233_232  .....................................................................................................
2a_chrX_234_233   ........*....................I.R.....................................................................
2a_chr1_235_234   .....................................................................................................
2a_chr12_236_235  ..........................S..........................................................................
2a_chr4_237_236   .............................S..................................................................G....
2a_chr5_238_237   ..............................................T........................E.............................
2a_chr8_239_238   .....................................................................................................
2a_chr5_240_239   .................V......W..................................................................G.........
2a_chr4_241_240   ........R.......................................................................A....................
2a_chr18_242_241  .......V...........................V...........L.....................................................
2a_chr8_243_242   .....................................G...........................................................V...
2a_chr3_244_243   ..............................T............I................................T........................
2a_chr4_245_244   ...........................................................R......................S..................
2a_chr6_246_245   ...................R.................................................................................
2a_chr7_247_246   ....................................T...............Y....G........................................*..
2a_chr5_248_247   ....................I...................................................................R............
2a_chr2_249_248   .................................................................S....D...........S..................
2a_chr2_250_249   ..............................................T......................................N...............
```

```
    1.2_o_50%_cn  YSEL-EDIQTKGKEVENFEKNLEECITRITNTEKCLKELMELKTKA-EL-EECRSLRS-C-QLEERVS-MEDEMNEMK-EGKFREKRIKRNEQSLQEIWDY
2a_chr6_251_250   ------^------^------^------^------^----+++--^------^------^------^------^------^------^------^------^
                  ..................X....X..........................................I..................................
2a_chr1_252_251   ....................................................................................K................
2a_chr3_253_252   ........................................K...NT.............H..........................I..............
2a_chr1_254_253   .......................................I.................................................K...........
2a_chr15_255_254  .........................................................................................G...........
2a_chr2_256_255   .................................N....V..............................T...............................
2a_chr2_257_256   ....................I..................T....................................................LH.......
2a_chr22_258_257  .....................................................................................................
2a_chr7_259_258   ...........................................................................................Q.........
2a_chr20_260_259  ........*..............D..........................K..............................................V...
2a_chr1_261_260   ......................K..............................................................................
2a_chr5_262_261   .............X....K....................X..........................................................R..
2a_chr2_263_262   .................I................*..................................................................
2a_chr10_264_263  ........................FV.............I.............................................................
2a_chr15_265_264  ..................................................................................S........*.........
2a_chr6_266_265   ........................Y.........................................I..................................
2a_chr4_267_266   ...............................................................Q.....................N...............
2a_chr13_268_267  ...............................I......................................................I..............
2a_chr4_269_268   ..................................G......................G.....................D......T......N.......
2a_chr12_270_269  .................I.....................T.............................................................
2a_chr4_271_270   ........................G............................................................................
2a_chr14_272_271  .Y.............Q................Q.*..................................................................
2a_chr8_273_272   ........................Y..S.I......N.................................................G............N.
2a_chr4_274_273   ..................................................................................S..................
2a_chr12_275_274  .......V......................D.................................................R....................
2a_chr13_276_275  .....................................................................................................
2a_chr9_277_276   .............Q..........................G..I.........................................................
2a_chr14_278_277  .............................P.......Q............................................................*..
2a_chr10_279_278  .....................................V...............................................................
2a_chr2_280_279   .....................................................................................................
2a_chr11_281_280  .............G............N..........................................................................
2a_chr3_282_281   ......N............................................................................T.........I.......
2a_chr5_283_282   ............................K.K......................................................................
2a_chr14_284_283  ...........................................................R...K.S................................*..
2a_chr6_285_284   ............................M.............................................S.................E........
2a_chr4_286_285   ..V..................S..S............................................................................
2a_chr5_287_286   .....................................................................................................
2a_chr3_288_287   .................X.........................................R.........................................
2a_chr6_289_288   ..A....................................T........................................A....................
2a_chr3_290_289   .....................................................................................................
2a_chr9_291_290   ..................K...............................V..................................................
2a_chr16_292_291  ..................................................................................S..................
2a_chr9_293_292   ....................................................................................................H
2a_chr18_294_293  ..............................................................P......................................
2a_chr1_295_294   ....................................................R....................I...............I...........
2a_chr2_296_295   .................L...................................................................................
2a_chr5_297_296   .................V.......................................................................G..R........
2a_chr15_298_297  ........................G..................................R...............K..........I..............
2a_chr15_299_298  ..................G......................................................................T...........
2a_chr8_300_299   ...........D........K..........................................................Q.....................
2a_chr4_301_300   ..................................................................................S..................
2a_chr13_302_301  .T......................G.........................................................L....X.......P.....
2a_chr13_303_302  ........................Y..................................................................K.........
2a_chrX_304_303   ...................R.................................................................................
2a_chr4_305_304   ................................Q....................................................................
2a_chrX_306_305   .............Q..................G........................................................G.QK........
2a_chr4_307_306   ...........................................................*......I..................................
2a_chr12_308_307  .................SX..............................................T...................................
2a_chr7_309_308   .....................................................................................................
2a_chr16_310_309  ........E............................................................T............V..................
2a_chr11_311_310  ....................................................R................................................
2a_chr18_312_311  ..................................S...............K..................................................
2a_chr8_313_312   .....................................................................................................
2a_chr2_314_313   .........................................................................I...........................
```

```
1.2_o_50%_cn      YSEL-EDIQTKGKEVENFEKNLEECITRITNTEKCLKELMELKTKA-EL-EECRSLRS-C-QLEERVS-MEDEMNEMK-EGKFREKRIKRNEQSLQEIWDY
                  ------^------^------^------^------^----+++--^------^------^------^------^------^------^------^
2a_chr5_315_314   .....................V................M...........................................S..................
2a_chr7_316_315   .................X................................................................S..............L...
2a_chr10_317_316  .....................................................................................................
2a_chr2_318_317   ................................K.................................................V..................
2a_chr9_319_318   ........R...............Y............................................................................
2a_chr3_320_319   .................S..................................................................X.K..X...........
2a_chr5_321_320   ............................................................................K...........E............
2a_chr6_322_321   ..K........................T.......................................................G.....K...........
2a_chr13_323_322  ..................................................................................S..................
2a_chr8_324_323   .....................................................................................................
2a_chrX_325_324   ......................................................R....S.E.....................T.................
2a_chr18_326_325  .....................................A............................................S..................
2a_chr8_327_326   ...........................................S.........................................................
2a_chr18_328_327  ...................................I..............................................L..................
2a_chr9_329_328   ..........................I..........................................................................
2a_chr15_330_329  ......................AV.........N.................................................................G.
2a_chr8_331_330   ........E............................................................................................
2a_chr8_332_331   ...........................................................S......................................*..
2a_chr20_333_332  ..............A.......................W....A..............................K.......S...............L..
2a_chr11_334_333  ........................Y.....................................V....................................N.
2a_chr11_335_334  ......G............N...............S.................*...............................................
2a_chr5_336_335   .........................T...............................................................X...........
2a_chr18_337_336  ...............................I..................................................S..................
2a_chr8_338_337   ...............................K..................................................S..................
2a_chrX_339_338   ........................Y............................................................................
2a_chr7_340_339   .......V.............V..................................G............................................
2a_chrX_341_340   ......E..................................Q.......................................N...................
2a_chr6_342_341   .....................................................................................................
2a_chr2_343_342   ...............................R.....................................................................
2a_chr15_344_343  .......................................T.............................................................
2a_chr10_345_344  ..........................................................................................K..........
2a_chr15_346_345  .................L........I...............N.....Q..........R.............................G.........G.
2a_chr15_347_346  ..........................................Q..........................................................
2a_chr4_348_347   ..............................S...........................................S.............N............
2a_chr16_349_348  .....................................................................................................
2a_chr10_350_349  ......................G..............................................................................
2a_chr9_351_350   ...........................G......................................................S..................
2a_chr7_352_351   ....................................................W................................................
2a_chr4_353_352   ................................................I...................................X................
2a_chr4_354_353   ...I..................................................I....*......................S...............R..
2a_chr6_355_354   ...............................................................................X-.S..................
2a_chr11_356_355  ...........V.........................................................................................
2a_chrX_357_356   .....................................D...............................................................
2a_chr3_358_357   ........................Y.............................................................T..............
2a_chrX_359_358   ...........................T.......................................................T.................
2a_chr4_360_359   ......................D..T......................................................................D....
2a_chr1_361_360   ............N..................R..................................................S..................
2a_chr13_362_361  ...........................K...............................................................K.X.......
2a_chr5_363_362   ..................................................................................S..................
2a_chr1_364_363   .....................................................................................................
2a_chr2_365_364   ...........V........K........I...........................N...........................................
2a_chr5_366_365   ..................................................................I..................................
2a_chr18_367_366  ..................................................................................................*..
2a_chr8_368_367   .......................................................................E..S...............T..........
2a_chr15_369_368  ...............*.Y.................................K..............................................R..
2a_chr8_370_369   .....................................................................................................
2a_chr13_371_370  .....................................................................................................
2a_chr9_372_371   ...........................................................Y..............K.........K................
2a_chr18_373_372  .....................................................................................................
2a_chr8_374_373   ................................................*..Q..............................S..................
2a_chr1_375_374   ......................................V...........................................S..................
2a_chr11_376_375  .........................................................................IK..........................
2a_chr9_377_376   ......................................P...............N..............................................
2a_chr8_378_377   .........................................Q...........................................................
```

```
   1.2_o_50%_cn    YSEL-EDIQTKGKEVENFEKNLEECITRITNTEKCLKELMELKTKA-EL-EECRSLRS-C-QLEERVS-MEDEMNEMK-EGKFREKRIKRNEQSLQEIWDY
                   ------^------^------^------^------^----+++--^------^------^------^------^------^------^------^------^
2a_chr2_379_378    ...............................P.....................................................................
2a_chrX_380_379    .....................................................................................................
2a_chr2_381_380    .....................................*............................................S..................
2a_chr13_382_381   ...................................................................T.................................
2a_chr12_383_382   ...............................K............N..........................G.............................
2a_chr8_384_383    .....K....E.......................................................................S...............R..
2a_chr4_385_384    .............*..............R........................................................................
2a_chr10_386_385   ......................................................................................G.............C
2a_chr10_387_386   ..............................................................M..........I........................*..
2a_chr9_388_387    .............A........G..............................................................................
2a_chr5_389_388    ....................K.............................................................S.K................
2a_chr1_390_389    ..................................................................................S..................
2a_chrX_391_390    .....................................................................................................
2a_chr22_392_391   .....................................................................................................
2a_chr12_393_392   ...............................................................*.....................................
2a_chr18_394_393   .....................................................................................................
2a_chr7_395_394    ....................................................................................................
2a_chr18_396_395   .......................Q...........S..............................X..................................
2a_chr18_397_396   .....................................................................................................
2a_chr5_398_397    .P.............G.....................................................................................
2a_chr10_399_398   .....................................................................................................
2a_chrX_400_399    .P...........................N........V...............R..............................................
2a_chr1_401_400    .............................................T........................................I..............
2a_chr13_402_401   ..................................................................................................C..
2a_chr15_403_402   .........................................................................R...........................
2a_chr11_404_403   .....................................................................................................
2a_chr2_405_404    .....................................................................................................
2a_chr3_406_405    .............................IK......................................................................
2a_chr2_407_406    .................................................................................................M...
2a_chr8_408_407    .......................K................................G............................................
2a_chr9_409_408    .....................................................................................................
2a_chr14_410_409   ..................X..................................................................................
2a_chr2_411_410    ..............................K.................I....................................................
2a_chr3_412_411    .......................................I.............................................................
2a_chr1_413_412    ...........................................................................Q.........................
2a_chr18_414_413   .....................................................................................................
2a_chr1_415_414    ..................................................*..................................................
2a_chr1_416_415    .....................................................................................................
2a_chr3_417_416    ....................................................Y............................T.............K.....
2b_50_CG_417       .....................................................................................................
2b_chrY_2_419      .....K...............S...T........F.........................................R...........E............
2b_chr6_3_420      ....................D...........................................................A.V.........*........
2b_chr2_4_421      .....................................................................................................
2b_chr8_5_422      ........................................................................*............................
2b_chr4_6_423      ...........................................................*.....................................M...
2b_chrY_7_424      .........................V.............I...................................D......V..................
2b_chr7_8_425      ..........................................................................K............M....R........
2b_chrX_9_426      ...................................................................................K.................
2b_chr5_10_427     ......................D...........................................................V.A.T..........K...
2b_chr2_11_428     .P...................................................................................................
2b_chr3_12_429     D..............K.....................................................................................
2b_chrX_13_430     ...........R.........................................................................................
2b_chr7_14_431     ..............................................................P.....................................C
2b_chr2_15_432     ..................................................................................V..................
2b_chrX_16_433     ..D...............................................................................V..................
2b_chr4_17_434     ...................................F-X...............................................................
2b_chr6_18_435     ..................................................................................V..................
2b_chr3_19_436     .....................................................................................................
2b_chr5_20_437     ..................................S...............................................V..................
2b_chr3_21_438     ............................................................................K........................
2b_chr2_22_439     ............................................................................I.....V..................
2b_chr13_23_440    ............................R..............................*....D...................X.K..............
2b_chr3_24_441     .....................................................................................................
2b_chr18_25_442    ...........................................A........F...................K.........V..................
```

```
   1.2_o_50%_cn  YSEL-EDIQTKGKEVENFEKNLEECITRITNTEKCLKELMELKTKA-EL-EECRSLRS-C-QLEERVS-MEDEMNEMK-EGKFREKRIKRNEQSLQEIWDY
                 ------^------^------^------^------^----+++--^------^------^------^------^------^------^------^------^
2b_chr6_26_443   ..................................................................................V..................
2b_chr12_27_444  ..................................................................................V..................
2b_chrX_28_445   .............................S....................................................V........K.........
2b_chr12_29_446  ............R......................................................................T...M.............
2b_chr5_30_447   ..................................................................................V.............D....
2b_chr8_31_448   .....................................................................V............V..................
2b_chr9_32_449   .....................................................................................................
2b_chr13_33_450  .....................................................................................................
2b_chr20_34_451  ........................Y............................................................................
2b_chr11_35_452  .........................................................R...................................T.......
2b_chr4_36_453   ..................................................................................V.X................
2b_chr6_37_454   .........................................X..............K.........................V..................
2b_chr18_38_455  .....................................................................................................
2b_chr3_39_456   ..................................................................................V................G.
2b_chr2_40_457   .............................................................................N....V..................
2b_chr3_41_458   ..................................................................................V..................
2b_chr1_42_459   .............................................V....................................V..................
2b_chr6_43_460   ..................................................................................V.......H..........
2b_chr3_44_461   .........................L...........................................................................
2b_chr21_45_462  ........................Y......A...........................W.........................................
2b_chr7_46_463   ...................N.................................................................................
2b_chr12_47_464  .....................................................................................................
2b_chr3_48_465   .......................K.............................................................................
2b_chr11_49_466  ..........................N..I...............S.......................................................
2b_chr14_50_467  .................V......................................................................R............
2b_chr12_51_468  ..A.....................................G............................................................
2b_chr12_52_469  ...............K.....................................................................................
2b_chr13_53_470  ...............................K.........................................................K..........H
2b_chr7_54_471   .....................................................................................................
2b_chr5_55_472   ......................Q..................M..........H...........G........I...........................
2b_chr4_56_473   ..T..........................S.......................................................................
2b_chr2_57_474   ................KL...................................................................................
2b_chr14_58_475  .F..................S................................................................................
2b_chrX_59_476   ................H....................................................................................
2b_chr10_60_477  .....................................................................................................
2b_chr13_61_478  ........................G..............T..............................G..............................
2b_chr7_62_479   ...................Q..............................................I*.........................N.......
2b_chr2_63_480   ...............................................................................*..................G..
2b_chr1_64_481   ......................................P..............................................................
2b_chr7_65_482   ..................................................................................L..................
2b_chr4_66_483   ...........................................................................A...............G.........
2b_chr8_67_484   .........................................................................T..............T............
2b_chr8_68_485   ..Q..................................................................................................
2b_chr2_69_486   .....D...I........G............I.........................................V.......................V...
2b_chr8_70_487   ...I.................................................................................................
2b_chr3_71_488   .................................................................M...................................
2b_chr13_72_489  ......................................V....................................................Q.........
2b_chr15_73_490  .....................................................................................................
2b_chr12_74_491  .................X...................................................................................
2b_chr1_75_492   ..................X.........................................................................K........
2b_chr7_76_493   ....................I....................................................R...........................
2b_chr3_77_494   .....................................................................................................
2b_chr17_78_495  .......F................Y............................................................................
2b_chr14_79_496  ...I..................*............S.................................................................
2b_chr8_80_497   .............................N.......................................................................
2b_chr3_81_498   ........K.....L......................................................................................
2b_chr2_82_499   .....................................................................................................
2b_chr8_83_500   ..................................................................................S..................
2b_chr20_84_501  ................................................................................E.SX.................
2b_chr14_85_502  ....................K............................................K...................................
2b_chr5_86_503   ............................................X........................................................
2b_chr17_87_504  .....................................................................................................
2b_chr12_88_505  ........................R............................................................................
2b_chr7_89_506   .....................*.........................D............................................X........
```

```
   1.2_o_50%_cn  YSEL-EDIQTKGKEVENFEKNLEECITRITNTEKCLKELMELKTKA-EL-EECRSLRS-C-QLEERVS-MEDEMNEMK-EGKFREKRIKRNEQSLQEIWDY
                 ------^------^------^------^------^----+++--^------^------^------^------^------^------^------^------^
2b_chr18_90_507  ..............................................................*.............*.........R...............
2b_chr7_91_508   .............................A.................................................................P.V...
2b_chr11_92_509  .................................................................................................V...
2b_chr2_93_510   .................................................................................................V...
2b_chr15_94_511  ....................................................................................................H
2b_chr15_95_512  .........S...........................................................................................
2b_chr10_96_513  ............N............................................R...........................................
2b_chr9_97_514   .........................................Q.............................................V.I...........
2b_chr3_98_515   .........................................................G.........*..K..............................
2b_chr17_99_516  ..........T..........................................................T...............................
2b_chr19_100_517 ...........................................................Y..........................Q..............
2b_chr6_101_518  ..................................................................................................C..
2b_chr17_102_519 .....................................................................................................
2b_chr3_103_520  ..................Q..................................................................................
2b_chr3_104_521  .....................................................................................................
2b_chr2_105_522  .........................................................N...........................................
2b_chrX_106_523  ...................................*...........................................K.....................
2b_chr8_107_524  .....................................................................................................
2b_chr12_108_525 ...........................................................................................Q.........
2b_chr1_109_526  .....................................................................................................
2b_chr17_110_527 ...............................................D.....................................................
2b_chr1_111_528  ..........................................Q..............................I...........................
2b_chrX_112_529  .............................I.......................................................................
2b_chr7_113_530  ....................I....................................................R...........................
2b_chr20_114_531 .....................................................................................................
2b_chr6_115_532  .....................................................................................................
2b_chr13_116_533 ......................................M..M..............................QI...........................
2b_chr6_117_534  .....................................................................................................
2b_chr8_118_535  .....K....E...............................Q..............................................T...........
2b_chr12_119_536 ......................A....................................F.............I...........................
2b_chr8_120_537  ..Q..................................................................................................
2b_chr3_121_538  ........................................K................R...........................................
2b_chr18_122_539 .................................................................................N...................
2b_chr4_123_540  ....................S.............................................................V..................
2b_chr6_124_541  ..................................................................................V....V.............
2b_chr5_125_542  ......................................................................*...........V............*.....
2b_chr6_126_543  .....................................................................................................
2b_chr14_127_544 .....................................K...............................................................
2b_chr4_128_545  .....................................................................................................
2b_chr10_129_546 ...V.................................................................................................
2b_chrX_130_547  ...............K.....................................................................................
2b_chr20_131_548 .....................................................................................................
2b_chr16_132_549 ..................................................................................V..................
2b_chr16_133_550 ..................................................................................V..................
2b_chr9_134_551  ..........................P........................Q..............................V..................
2b_chr1_135_552  ................................................I........................................SS..........
2b_chr2_136_553  .....................................Q................................*X..........V..................
2b_chr5_137_554  ...........................................................................A......V..................
2b_chr12_138_555 ..................................................................................V..................
2b_chr12_139_556 ..................................................................................V..................
2b_chr2_140_557  ..................................................................................V.....N............
2b_chr9_141_558  .....K...............................................................................................
2b_chr2_142_559  ..................X..............................................G...................................
2b_chr3_143_560  .....................................................................................................
2b_chr2_144_561  ...............K...................V.......................R.........................................
2b_chr9_145_562  .....................................................................................................
2b_chrY_146_563  ...........................G..........R.K..........................................................N.
2b_chrY_147_564  ...........................G..........R.K..........................................................N.
2b_chr11_148_565 ......N.................................Q............K...N.................................D......*..
2b_chrY_149_566  .......................................................................................M.............
2b_chr12_150_567 .......................*..............V..............K...............................................
2b_chrX_151_568  ................................................................................V......M.G........*..
2b_chr3_152_569  ....................Y...Y...........................S......R.........................................
2b_chr11_153_570 .F.....T.............................................................................................
```

```
     1.2_o_50%_cn YSEL-EDIQTKGKEVENFEKNLEECITRITNTEKCLKELMELKTKA-EL-EECRSLRS-C-QLEERVS-MEDEMNEMK-EGKFREKRIKRNEQSLQEIWDY
                  ------^------^------^------^------^----+++--^------^------^------^------^------^------^------^------^
  2b_chrX_154_571 ......E...........................S........A...............................................Q.........
  2b_chr7_155_572 ........................Y............................................................................
  2b_chr7_156_573 ......................................M............................L..............S..................
  2b_chr2_157_574 ...............................................................G......................T..............
  2b_chr3_158_575 .........................................................................................T...........
  2b_chr8_159_576 ......E.........H....I..................K..................................G............Q..K.........
 2b_chr14_160_577 ...P............................K..........................Y...............D.........................
  2b_chr7_161_578 ............................................................................X........................
  2b_chrY_162_579 .............................................V....................I...................T........H...Y.
 2b_chr11_163_580 .....................................................................................................
  2b_chr3_164_581 .........................................................R...........................................
  2b_chr9_165_582 .....................................................................................................
  2b_chr7_166_583 ......................................................................................S.R........V...
  2b_chr8_167_584 ......E..............................................................................................
  2b_chr6_168_585 ...........................................................*.....................................V...
 2b_chr16_169_586 .................S.......T..............................S............I..Q............................
  2b_chr4_170_587 .....................................................................I.............G.................
  2b_chr5_171_588 ..............A.......................M....................R....................V.S..................
  2b_chrX_172_589 ......................*..............................................................................
  2b_chr7_173_590 ..............GG..................G....................H.............................................
 2b_chr14_174_591 .....D...I........G............I...........................W........................X................
 2b_chr18_175_592 ...............K....................................R..................E...................K.........
  2b_chr3_176_593 ..................X..................................................................................
  2b_chrX_177_594 ...............K...........................................S......................S..................
 2b_chr11_178_595 .....................................................................................................
  2b_chr5_179_596 .....................V.......I....R..................................................................
  2b_chr3_180_597 ...............................I.....................................................................
  2b_chr8_181_598 ........................................................................................T.........G..
 2b_chr11_182_599 .......................G...................................R..............S..........................
  2b_chr3_183_600 .Y.....T.............................................................................................
  2b_chrX_184_601 .......................................................................................K.............
  2b_chrY_185_602 .....................................................................................................
  2b_chr2_186_603 ..............L................................D.....................................................
  2b_chr7_187_604 ............................................R....................................................L...
  2b_chr1_188_605 ................T...................................................................D................
  2b_chr1_189_606 ......................D........R.....................................................................
 2b_chr17_190_607 ...................R......S..........................................................................
  2b_chr1_191_608 ....................K................................................................................
 2b_chr20_192_609 .....................................................................................................
 2b_chr11_193_610 .............*.D...........................I.........................................................
  2b_chrX_194_611 ...........................................................................................K.........
  2b_chr3_195_612 ......................D..............................................................................
  2b_chr4_196_613 .................................................................T...V........................F..L...
 2b_chr13_197_614 ........................Y.........................................I..................................
  2b_chr8_198_615 ..........E........................F............Q...............Q........I...........................
  2b_chr8_199_616 .....................................L.L............R................................................
  2b_chr1_200_617 ..................................................................L..................................
  2b_chr2_201_618 .......................D.............................*...................V...........................
  2b_chr4_202_619 .......T................................Q..................................................K.........
  2b_chr2_203_620 ...............................................................................*..................G..
  2b_chr3_204_621 ..................Q..................................................................................
  2b_chr4_205_622 .............K.......................................................................................
 2b_chr21_206_623 .....K..................................................................K......K....................C
  2b_chr2_207_624 ...........................................P.............................T...........................
  2b_chr2_208_625 .............................N...........................R...........................................
 2b_chr15_209_626 ...........D.................S.......Q.......V...................K...................................
  2b_chr2_210_627 .................X.......................................R........................................R..
  2b_chr6_211_628 ..................................................................I..................................
 2b_chr11_212_629 ..D....TR.............A.....T..............................*.........................................
  2b_chr9_213_630 ...............K.....*............Y..................................................................
  2b_chr4_214_631 ......................D.......SI.....................................................................
  2b_chr2_215_632 X..........C.*......................................................................D................
  2b_chr3_216_633 .....................................................................................................
 2b_chr20_217_634 ....................T...............................G.N..K......................E....................
```

```
    1.2_o_50%_cn  YSEL-EDIQTKGKEVENFEKNLEECITRITNTEKCLKELMELKTKA-EL-EECRSLRS-C-QLEERVS-MEDEMNEMK-EGKFREKRIKRNEQSLQEIWDY
                  ------^------^------^------^------^----+++--^------^------^------^------^------^------^------^------^
2b_chr8_218_635   .........................................................R.........................................G.
2b_chr15_219_636  .........................V........G..................................................................
2b_chr5_220_637   .........................................................................I.............T.............
2b_chr4_221_638   .............................S......................................................................D
2b_chr7_222_639   ......................................................R.........................E.S..........R.......
2b_chr10_223_640  .....................................................................................................
2b_chr8_224_641   ...................................................................................................V.
2b_chr5_225_642   .....................................Q............................................S..................
2b_chr16_226_643  ..................................................D..................................................
2b_chr12_227_644  ...P............T.....................P...........................I..................................
2b_chr7_228_645   ............................................................................I..............Q.........
2b_chrX_229_646   ...............................R.....................................................................
2b_chr7_230_647   ...............................RD...............P..................L.V...............................
2b_chr4_231_648   .....................................................................................................
2b_chr9_232_649   .....................*..................Q............................................................
2b_chr15_233_650  .....................................................................................................
2b_chr13_234_651  .....................................................................................................
2b_chr5_235_652   .....................................................................................................
2b_chr15_236_653  .........S.............................................................G.............................
2b_chr6_237_654   ............................K...............................................L...........Q.....F......
2b_chr3_238_655   .............K...........................................................K...............KK..........
2b_chr14_239_656  .....................................................................................................
2b_chr7_240_657   ......................................................R...............................I..............
2b_chr6_241_658   .........................V.....................................................................H.....
2b_chr2_242_659   ....................S................................................................................
2b_chr4_243_660   ......................................................R..............................................
2b_chr7_244_661   ...........................................A.........................................................
2b_chr8_245_662   .....................................................................................................
2b_chr4_246_663   ................................................V....................................................
2b_chr6_247_664   ...............K........................................................Q............................
2b_chrX_248_665   .....................................................................................................
2b_chr4_249_666   ...............K.....................................................................................
2b_chrX_250_667   ...........X..........................................T..................R...........................
2b_chr9_251_668   ..................................................................................V..................
2b_chr11_252_669  ...................................................................*.................................
2b_chr3_253_670   .......................................................................E.............................
2b_chr2_254_671   .......................................................H........................R....................
2b_chr14_255_672  ..................................................................................L...S..............
2b_chr5_256_673   ...................N.................................................................................
2b_chr3_257_674   .....D..............................................Y................I......................*........
2b_chr6_258_675   .....................................................................................................
2b_chrX_259_676   ........................Y..............................FS..F....................................K....
2b_chr5_260_677   .......................D...................................*.........................................
2b_chr10_261_678  ..................................Y..................................................................
2b_chr18_262_679  .................................*...................................................................
2b_chr20_263_680  .........................................................................I...........................
2b_chr12_264_681  ........................................Q............................................................
2b_chr5_265_682   ........E..V..............................................................S....................E.....
2b_chr4_266_683   ...........................................................*.........................................
2b_chr9_267_684   ....................T......................................................K.N.D.......M.............
2b_chr18_268_685  ......A............................................................L.....................K...........
2b_chr14_269_686  .....................................................................................................
2b_chr5_270_687   ......................D........K..Y.........................................I.............K..........
2b_chr2_271_688   ...................Q.S...............................................................................
2b_chr20_272_689  ..................................................................A...K..............................
2b_chr2_273_690   .......V............................................................................D................
2b_chr10_274_691  ..................................................................L..................................
2b_chr3_275_692   .........................................................N...........................................
2b_chr11_276_693  .........................................................................X-X.........................
2b_chr1_277_694   ................................................................................E............T.......
2b_chr11_278_695  .....................................................................................................
2b_chr8_279_696   ...............D............T.........................R..............................................
2b_chr16_280_697  .......................................................................................L....*........
2b_chr4_281_698   .............................................T......S...................................N.........R..
```

```
    1.2_o_50%_cn  YSEL-EDIQTKGKEVENFEKNLEECITRITNTEKCLKELMELKTKA-EL-EECRSLRS-C-QLEERVS-MEDEMNEMK-EGKFREKRIKRNEQSLQEIWDY
                  ------^------^------^------^------^----+++--^------^------^------^------^------^------^------^------^
 2b_chr2_282_699  .....................................................................................................
 2b_chr7_283_700  ................S.....................V.................................*............................
 2b_chr5_284_701  ....................................................H............S..................................C
2b_chr12_285_702  ...........S...........................................R.............................................
 2b_chr5_286_703  ...................N................................*................................................
 2b_chrX_287_704  ..................................Y.......................................................Y..........
2b_chr12_288_705  ...................N.................................................................................
2b_chr11_289_706  .....................................................................................................
 2b_chrX_290_707  ..D........................................................W.......................................E.
2b_chr10_291_708  .....................................................................................................
2b_chr12_292_709  ....................I................................................................................
 2b_chr5_293_710  ...........................................................*..........A..............................
2b_chr14_294_711  ......................K..............................................................................
 2b_chr9_295_712  ...............G.....................................................................E...............
 2b_chrY_296_713  .....................................................................V.........K.....................
 2b_chr7_297_714  .....................................................................................................
 2b_chr2_298_715  .....................................................................................................
2b_chr12_299_716  .......................................I.................................I..........Q................
2b_chr16_300_717  ..................................................K.................................D................
 2b_chr1_301_718  ......N.................................................................................R.S..........
2b_chr15_302_719  ..Q....................................T.............................................................
 2b_chr7_303_720  .........A...........F......................................................I........................
 2b_chr2_304_721  .........................M...............V........................................V..........N.......
 2b_chr9_305_722  .................................................................................................V...
 2b_chr9_306_723  .................................................................................................V...
 2b_chr4_307_724  ..............D.........................................S....H.......................................
 2b_chr4_308_725  ........................Y..............................................................M..........C..
2b_chr17_309_726  ..K..................................................................................................
 2b_chr2_310_727  ..........................I..........................................................................
2b_chr15_311_728  .....................................................................................................
 2b_chr7_312_729  ....................................X.................................................G..............
 2b_chr8_313_730  ..................G..................................................................................
2b_chr15_314_731  .....................................................................................................
 2b_chr2_315_732  ...........................................................*..P......................................
 2b_chr6_316_733  ................................................................................................*.*..
 2b_chr2_317_734  .............................I.......................................................................
2b_chr14_318_735  ...............D.....................................................................................
 2b_chr1_319_736  .........S........................*........................Y.........................................
 2b_chr6_320_737  ........................................................G............................................
 2b_chr2_321_738  ...........................................................Y.........................................
 2b_chr4_322_739  ............................LI.......................................................................
 2b_chr1_323_740  ..K.................................................................................D.............R.C
 2b_chr2_324_741  .....................................................................R...............................
 2b_chr2_325_742  .....................................................................R..........R....................
 2b_chr6_326_743  ..................................................................A..................................
 2b_chr8_327_744  ..........................................................................................T..........
2b_chr14_328_745  ..............L......................................................................................
 2b_chr1_329_746  ......................D..............................T...............T...I...........................
2b_chr10_330_747  .........S...............K...........................................................................
 2b_chr6_331_748  ...............................K..................................I..............N..X.KK.....R.......
 2b_chr2_332_749  .....................*...............................................................................
2b_chr16_333_750  ..................................W..................................................................
 2b_chr6_334_751  ................................G...........................................I........................
2b_chr17_335_752  ....................................................................................................C
 2b_chr5_336_753  .................................................................................T...................
2b_chr13_337_754  ................................Q....................................................................
2b_chr12_338_755  ...............................................D..................................................*..
 2b_chr4_339_756  .....................................................................................................
 2b_chr6_340_757  ...........................................................................................QK........
 2b_chr5_341_758  ......E....................................................R.........................................
 2b_chr8_342_759  ..................................................................................V..................
 2b_chr2_343_760  ..............A............................P.........................................................
2b_chr12_344_761  .....................................................................................................
 2b_chr8_345_762  ...........................................................................V...............K.........
```

```
     1.2_o_50%_cn YSEL-EDIQTKGKEVENFEKNLEECITRITNTEKCLKELMELKTKA-EL-EECRSLRS-C-QLEERVS-MEDEMNEMK-EGKFREKRIKRNEQSLQEIWDY
                  ------^------^------^------^------^----+++--^------^------^------^------^------^------^------^
  2b_chr2_346_763 .......................................I.............................................................
  2b_chr1_347_764 ..................................Y..................................................................
 2b_chr11_348_765 .....................................................................................................
 2b_chr15_349_766 .....................................................................................................
 2b_chr10_350_767 ...........................T......Y...................................K..............................
  2b_chr8_351_768 ............................................................................X.....................Q..
  2b_chrX_352_769 ..................K.....................................G............................................
  2b_chr4_353_770 ...........................................................................................G........C
  2b_chr1_354_771 ................D....................................................................................
  2b_chr8_355_772 .........................................................N...........................................
  2b_chr5_356_773 .......................................T.............................................................
 2b_chr10_357_774 ...................................................................................G.................
 2b_chr12_358_775 ....................S..........................................*.....................................
  2b_chr3_359_776 .....................................................................................................
  2b_chr3_360_777 ................................G....................................................................
 2b_chr11_361_778 .....................................................................................................
  2b_chrX_362_779 ................................K..............K.....................................................
  2b_chr1_363_780 .......................................K.................C...............I...........................
  2b_chr7_364_781 .........S...............................................................T...........................
  2b_chr4_365_782 ...............................................A..A........*.........................................
 2b_chr10_366_783 .....................................................................................................
  2b_chr8_367_784 ...........................S...............A.V.......................................................
 2b_chr15_368_785 .....................................................................................................
  2b_chr6_369_786 .....................................................................................................
 2b_chr10_370_787 .............A.........A.................................................................I...........
  2b_chr1_371_788 .....................................................................................................
  2b_chr3_372_789 .....................................................................................................
  2b_chr9_373_790 ................................*....................................................................
 2b_chr14_374_791 ...............D.....................................................................................
 2b_chr16_375_792 .....................................................................................................
  2b_chr8_376_793 .....................................................................................................
  2b_chrX_377_794 .................................................................................................M..D
  2b_chr4_378_795 ...........D..D.......................................N..........................R...................
 2b_chr12_379_796 ......................*....................A.........................T...............................
  2b_chrX_380_797 .............................................................................R......D.............R..
  2b_chr1_381_798 ...................................................*.................................................
 2b_chr13_382_799 ......................................................................................I..............
  2b_chr1_383_800 ....................H................................................................................
  2b_chrY_384_801 .....................................................................V..............................S
  2b_chr5_385_802 .....................................................................................................
 2b_chr11_386_803 ...................E.................................................R.................L.............
 2b_chr21_387_804 .....................................................................................................
  2b_chr3_388_805 .................................................................GX..................................
 2b_chr18_389_806 ..................Q........................................R.........................................
 2b_chr18_390_807 .....................................................................................................
 2b_chr10_391_808 .....................................................................R................G..............
 2b_chr12_392_809 .............Q...............................................M......................................
  2b_chr5_393_810 .....................................................................................................
  2b_chrX_394_811 .................XG..........S.............................R......................V..................
  2b_chr1_395_812 ....................I.......................N..........................................K.............
  2b_chr2_396_813 .....................................................................................................
  2b_chr4_397_814 ................................D..........................Y.........................................
  2b_chrX_398_815 .T..............................................V...................................G................
 2b_chr10_399_816 .....................................................................................................
  2b_chr4_400_817 .............Q.......................................................................................
  2b_chr3_401_818 .....................................................................................................
 2b_chr20_402_819 ......................*..................................................................I...........
  2b_chr3_403_820 .....................................................................................................
  2b_chr5_404_821 ...........................................................*.........................................
 2b_chr10_405_822 .....................................*...............................................................
  2b_chrY_406_823 ..........E..........................................................................................
  2b_chr8_407_824 .....................................................................................................
 2b_chr18_408_825 ......................................................................Q..............................
  2b_chr3_409_826 .....................................................................................................
```

```
      1.2_o_50%_cn YSEL-EDIQTKGKEVENFEKNLEECITRITNTEKCLKELMELKTKA-EL-EECRSLRS-C-QLEERVS-MEDEMNEMK-EGKFREKRIKRNEQSLQEIWDY
                   ------^------^------^------^------^----+++--^------^------^------^------^------^------^------^------^
2b_chr10_410_827  .C..........................................................................................K........
2b_chr13_411_828  .....................................................................................................
2b_chr11_412_829  .............K.......................................................................................
2b_chr3_413_830   .........................................................................I...........................
2b_chr4_414_831   .....................................................................................................
2b_chr2_415_832   .....................................................................................................
2b_chr6_416_833   .....................................................................................................
2b_chr3_417_834   ..................X........................S.........................................................
2b_chr5_418_835   ....................K....K...........................................................................
2b_chr3_419_836   ....................H................................................................................
2b_chr8_420_837   .....................................................................................................
2b_chr2_421_838   ...........................................P.........................................................
2b_chr3_422_839   .....................................................................................................
2b_chr15_423_840  .....................................................................................................
2b_chr5_424_841   .....................................................................................N...............
2b_chr1_425_842   .....................................................................................................
2b_chr4_426_843   .....................................................................................................
2b_chr10_427_844  .....................................................................................................
2b_chr5_428_845   .....................................................................................................
2b_chr11_429_846  .....................................................................................................
2b_chr19_430_847  .....................................................................................................
2b_chr8_431_848   ...............G.....................................................................................
2b_chr15_432_849  ........................................................................XX...........................
2b_chr12_433_850  .....................................................................................................
2b_chr18_434_851  .....................................................................................................
2b_chr2_435_852   .....................................................................................................
2b_chr4_436_853   ......................................................................................I..............
2b_chr12_437_854  .....................................................................................................
2b_chr16_438_855  .......................................................................A.............................
2b_chr5_439_856   ......................................................................................I..............
2b_chr9_440_857   ...........................................A.........................................................
2b_chr6_441_858   .....................................................................................................
2b_chr4_442_859   .....................................................................................................
2b_chr3_443_860   .....................................................................................................
2b_chrX_444_861   .....................................................................................................
2b_chr5_445_862   .....................................................................................................
2b_chr8_446_863   .....................................................................................................
2b_chr5_447_864   .....................................................................................................
2b_chr1_448_865   .....................................................................................................
2b_chr2_449_866   .....................................................................................................
2b_chr8_450_867   .....................................................................................................
2b_chr7_451_868   .....................................................................................................
2b_chr11_452_869  .......L.............................................................................................
2b_chr4_453_870   .....................................................................................................
2b_chr8_454_871   .P.....................G...............................................................KE............
2b_chr8_455_872   .....................................................................................................
2b_chr8_456_873   .........................................R.........Q...............................................V.
2b_chr4_457_874   .............*.......................................................................................
2b_chr2_458_875   .....................................................................................................
2b_chr3_459_876   D.............................T........................F.........................................V...
2b_chr2_460_877   ....................................................Y................................................
2b_chr6_461_878   .....................................................................................................
2b_chr12_462_879  ...........C.........................................G...............................................
2b_chr3_463_880   .....................................................................................................
2b_chr8_464_881   .............................P........M..............................................................
2b_chr1_465_882   .....................................................................................................
2b_chr10_466_883  ............................................................................V........................
2b_chr16_467_884  .........................T...........................................................................
2b_chr4_468_885   ........HS.................................I..........................Q........K.....................
2b_chr8_469_886   .......................V.............................................................................
2b_chr8_470_887   .....................................................................................................
2b_chr5_471_888   .....................................................................................................
2b_chr1_472_889   .....................................................................................................
2b_chr7_473_890   .................L.........................................................................A.........
```

```
    1.2_o_50%_cn  YSEL-EDIQTKGKEVENFEKNLEECITRITNTEKCLKELMELKTKA-EL-EECRSLRS-C-QLEERVS-MEDEMNEMK-EGKFREKRIKRNEQSLQEIWDY
                  ------^------^------^------^------^----+++--^------^------^------^------^------^------^------^------^
2b_chr13_474_891  .....................................................................................................
2b_chr18_475_892  .....................................................................................................
2b_chr18_476_893  ......................................................I.................................Q..G.........
2b_chr6_477_894   ................................................V..K.................................................
2b_chr10_478_895  .....................................................................................................
2b_chr15_479_896  ......................................P..............................................................
2b_chr9_480_897   ..................................................................................V..................
2b_chr2_481_898   .....................................................................................................
2b_chr21_482_899  .....................................................................................................
2b_chr2_483_900   ...............K.....................................................T...............................
2b_chr11_484_901  .....................................................................................................
2b_chr12_485_902  ...........................T.........................................................................
2b_chr3_486_903   ..*...G..............................................................................................
2b_chrX_487_904   ..............I.....K..X.........N...................................................................
2b_chr7_488_905   ..................................................................................V..................
2b_chr2_489_906   ..........................................................................................K..........
2b_chrX_490_907   .....................................................................................................
2b_chr13_491_908  ..................................*..................................................................
2b_chr2_492_909   ..................................................D......................T..........D................
2b_chr2_493_910   .....................................................................................................
2b_chr11_494_911  .............................................................R.......................................
2b_chr8_495_912   .......................................................R..........................V..................
2b_chr14_496_913  ...........V.........................................................................................
2b_chrX_497_914   .......S...................................A......................................V..................
2b_chr15_498_915  .....................................................................................................
2b_chr2_499_916   ...........V.........................................................................................
2b_chr1_500_917   .....................................................................................................
2b_chr6_501_918   ...............................................................K.....................................
2b_chr20_502_919  .....................................................................................................
2b_chr21_503_920  .....................................................................................................
2b_chr1_504_921   ...........................T...R.....................................................................
2b_chr21_505_922  ..................................................................................V.....Q......*.....
2b_chrX_506_923   .....................................................................................................
2b_chr1_507_924   ......H.........................................................................................*....
2b_chr11_508_925  .......................................................................................V.............
2b_chr7_509_926   ...........................................................W.........................................
2b_chr3_510_927   ....................................................................................X................
2b_chr2_511_928   .................*X..................................................................................
2b_chr15_512_929  .....................................................................................................
2b_chrX_513_930   .....................................................................................................
2b_chr1_514_931   .....................................................................................................
2b_chr17_515_932  ................................................................................................D....
2b_chr12_516_933  D....................................................................................................
2b_chr14_517_934  ........E............................................................................................
2b_chr7_518_935   .......................K.............K.........................................D.....................
2b_chr4_519_936   ........................Y.........................................I..................................
2b_chr11_520_937  .....................................................................................................
2b_chr1_521_938   ......H.....................................................................R........................
2b_chr14_522_939  .............................I.......................................................N...............
2b_chr18_523_940  ..................................S..........................L.......................................
2b_chr3_524_941   ........................Y............................................................................
2b_chr9_525_942   .....................................................................................................
2b_chr19_526_943  .....................................................................................................
2b_chr10_527_944  .....................................................................................................
2b_chr2_528_945   .....................................................................................................
```
