# Supplementary material for: Cryptic genetic variation enhances primate L1 retrotransposon survival by enlarging the functional coiled coil sequence space of ORF1p
Source: PLoS Genet. 2020 Aug 14;16(8):e1008991. doi: 10.1371/journal.pgen.1008991 (PMC7449397; doi:10.1371/journal.pgen.1008991)
Supplement: S9 Fig — Alignment of L1Pa2 CG-null coiled coil peptide sequences that have an F at position 134 vs the 50% consensus sequence of L1Pa2. (PDF) [file pgen.1008991.s009.pdf]

```
                        60        70        80        90       100       110       120       130       140       150
                --- ----|---- ----|---- ----|---- ----|---- ----|---- ----|---- ----|---- ----|---- ----|---- ----|--
        heptad     1  |   2  |   3  |   4  |   5  |  6 stm  |   7  |   8  |   9  |  10  |  11  |  12  |  13  |  14  |
                ------^------^------^------^------^----+++--^------^------^------^------^------^------^------^------^
                abcdefgabcdefgabcdefgabcdefgabcdefgabcdbcdefgabcdefgabcdefgabcdefgabcdefgabcdefgabcdefgabcdefgabcdefg
1.2_o_50%_cn    YSELREDIQTKGKEVENFEKNLEECITRITNTEKCLKELMELKTKARELREECRSLRSRCDQLEERVSAMEDEMNEMKREGKFREKRIKRNEQSLQEIWDY

a_L1PA2_2_1     F...-..V*.HH..AK.L..R.DKWL...NSV..S.ND......I.-..-DA.T.FN.-F-KV.....EI..Q.....-.E......L.............
a_L1PA2_3_2     H...-.EV*.N....KH.X.KTDNSV.....A..S..D.T.V..A.-..-DK.L..ST-*-......XV.........-.E..........K.........
a_L1PA2_5_3     ..K.-.EVR.N....K.L..K.D*WL.....E..S..D.....AM.-..-D..T..S.-.-...........K.....-.D........I......K...C
a_L1PA2_6_4     ....-.E.*.N....K.LX.K.D.WL.....A..S..D......V.-.I-D..T..S.-.-...A.............-.E.....G..S....FP.....
a_L1PA2_7_5     ....-K...............S...T....................-..-........-.-...............K.-.........E............
a_L1PA2_8_6     .C..-.E........K..G...D.R.P................A..-..-........-.-....T..V.........-.R....................
a_L1PA2_9_7     F...-..VR.QR..AK.C..R.DKWL....SV..S.ND..D...ME-..-DA.T.FS.-F-..KX...VT..Q.....-.E......V............H
a_L1PA2_10_8    .P..-.E........K.L....DK...G....Q.......D..A.V-..-........-F-.......V.........-....G...V.....N.......
a_L1PA2_11_9    *...-.....E............D......................-..-......K.-.-......T.V.....A..-......................
a_L1PA2_12_10   ....-.N...........X......V.........S..........-..-........-.-.................-...........ID......SE.
a_L1PA2_13_11   ....-...*...R.................................-..-........-*-.................-......................
a_L1PA2_14_12   ....-.........L.........R.....................-..-........-.-.................-......................
a_L1PA2_15_13   ....-................................*........-..-........-.-.................-......................
a_L1PA2_16_14   ....-...............H........................V-..-........-.-.........D.......-..N...................
a_L1PA2_17_15   ....-.........................................-..-........-.-.................-......................
a_L1PA2_18_16   ....-.........................................-..-........-.-.................-......................
a_L1PA2_19_17   ....-.........................................-..-........-.-.................-D.....................
a_L1PA2_20_18   ....-...........................D..........S..-..-........-.-.................-........M.............
a_L1PA2_21_19   ....-.........................................-..-........-.-.................-........T.............
a_L1PA2_22_20   ....-.........................................-..-........-.-.................-......................
a_L1PA2_23_21   .P..-K........................................-..-........-.-.................-..............R.......
a_L1PA2_24_22   ....-...............K......TV.................-..-........-.-............R....-......................
a_L1PA2_25_23   ....-.........................................-..-........-.-.................-....K............K....
a_L1PA2_26_24   ....-.........................................-..-........-.-.................-....................GC
a_L1PA2_27_25   ....-.........................................-..-........-.-..........N......-D.....................
a_L1PA2_28_26   ....-.........................................-..-........-.-...........G.....-.........Q............
a_L1PA2_29_27   ....-.........................................-..-........-.-...D...........V.-......................
a_L1PA2_30_28   ....-..S......................................-..-........-.-.................-......................
a_L1PA2_31_29   .C..-.........................................-..-........-.-.................-......................
a_L1PA2_32_30   ....-.........................................-..-....R...-.-.................-.....G................
a_L1PA2_33_31   ....-................V........................-..-........-.-.................-......................
a_L1PA2_34_32   ....-.................D.R.....................-..-........-.-.................-.....Q................
a_L1PA2_35_33   ....-................*........................-..-........-.-.................-.X....................
a_L1PA2_36_34   ....-.......................R...D.............-..-........-.-.................-......................
a_L1PA2_38_35   ....-...........S..................*..........-..-........-.-.................-.........Q.....P..T...
a_L1PA2_39_36   ....-.............X...........................-..-........-.-....S............-......................
a_L1PA2_40_37   ....-................................D........-..-........-.-.................-......................
a_L1PA2_41_38   ....-........K........A.......................-..-........-.-.................-......................
a_L1PA2_42_39   ....-.....................S...................-..-........-.-.......V.........-......................
a_L1PA2_43_40   ....-..............Q....F....S................-..-........-.-.................-......................
a_L1PA2_44_41   ....-.......................R.................-..-........-.-.................-......................
a_L1PA2_45_42   ....-.........................................-..-........-.-.................-.........*............
a_L1PA2_46_43   ....-.........................................-..-.......I-.-.................-......................
a_L1PA2_47_44   ....-......................................N..-..-........-.-.................-......................
a_L1PA2_48_45   ....-...L........L............................-..-........-.-.................-......................
a_L1PA2_50_46   ....-....................T....................-..-........-R-.................-......................
a_L1PA2_51_47   ....-...........T.............................-..-........-.-.................-......................
a_L1PA2_52_48   ....-.........................................-..-........-.-......AV.........-................K.....
a_L1PA2_53_49   ....-.........................................-..-........-.-.................-...................R..
a_L1PA2_54_50   .A..-.........................................-..-........-.-.................-................*...E.
a_L1PA2_55_51   ....-.........................................-..-........-.-...........K.....-.........E............
a_L1PA2_56_52   ....-..T................S.....................-..-........-G-.................-......................
a_L1PA2_57_53   ....-.........................................-..-........-.-.................-......................
a_L1PA2_58_54   ....-.........................................-..-........-.-.................-.............E........
a_L1PA2_59_55   ....-.........................................-..-........-.-.................-.........R............
a_L1PA2_60_56   ....-.....................................R...-..-........-.-.................-......................
a_L1PA2_61_57   ....-.........................................-..-........-.-...X.............-......................
```

```
1.2_o_50%_cn    YSELREDIQTKGKEVENFEKNLEECITRITNTEKCLKELMELKTKARELREECRSLRSRCDQLEERVSAMEDEMNEMKREGKFREKRIKRNEQSLQEIWDY
                ------^------^------^------^------^----+++--^------^------^------^------^------^------^------^------^
a_L1PA2_62_58   ...-..........................................-..-........-.-.................-......................
a_L1PA2_63_59   ...-..........................................-..-........-.-.................-......................
a_L1PA2_64_60   ...-..........................................-..-........-.-..K..............-......................
a_L1PA2_65_61   ...-..........................................-..-........-.-.................-......................
a_L1PA2_66_62   ...-................K.........................-..-........-.-.......V.........-......................
a_L1PA2_67_63   ....-..L......................................-..-........-.-.................-...................*..
a_L1PA2_68_64   ...-....................R.....................-..-........-.-...............V.-......................
a_L1PA2_69_65   ....-....A....................................-..-........-.-.................-......................
a_L1PA2_70_66   ....-..T......................................-..-........-.-.................-......................
a_L1PA2_71_67   ...-..........................................-..-........-.-.................-..............N.......
a_L1PA2_73_68   ...-............................D.............-..-........-.-.......T.........-..................V...
a_L1PA2_74_69   ...-................K.................Q.......-..-........-.-.................-......................
a_L1PA2_75_70   ....-G........................................-K.-........-.-.................-......................
a_L1PA2_76_71   .C.R-..............N..........................-..-........-.-.Q...............-.............K........
a_L1PA2_77_72   ...-....................G.......G.............-..-........-.-........R........-......................
a_L1PA2_78_73   ...-........................K.................-..-K.......-.-..QGK............-......................
a_L1PA2_79_74   ...-..................D.W.....................-..-........-.-...V.............-............K.........
a_L1PA2_80_75   ...-....X.....................................-..-K.......-.-.................-.E....................
a_L1PA2_81_76   ...-.....................M....................-..-........-.-.................-......................
a_L1PA2_82_77   ...-.............Y.........G..................-..-........-.-.......V.........-......................
a_L1PA2_83_78   ...-.............................Q............-..-........-.-.................-.......*..............
a_L1PA2_85_79   ....-..M............H.........................-..-........-.-.................-.................D....
a_L1PA2_86_80   ...-..........................................-..-........-.-.................-......................
a_L1PA2_87_81   ...-..........................................-..-........-*-.........V.......-......................
a_L1PA2_89_82   ...-..........................................-..-........-.-.................-......................
a_L1PA2_90_83   ...-..........................................-..-........-.-........I........-...............F......
a_L1PA2_91_84   ...-..........................S.............E.-..-........-.-.................-......................
a_L1PA2_92_85   ...-..........................................-..-........-.-.................-......................
a_L1PA2_93_86   ...-..........................................-..-........-.-....T..........T.-......................
a_L1PA2_94_87   ...-..........I.......................V.......-..-........-.-.................-.......*..............
a_L1PA2_95_88   N..-...........................I..............-..-........-.-.................-......................
a_L1PA2_96_89   ...-..........................................-..-........-.-.................-.........T............
a_L1PA2_97_90   ...-..........................................-..-........-.-.................-......................
a_L1PA2_98_91   ...-...........D............................E.-..-........-.-......P..........-......................
a_L1PA2_99_92   ...-...................................T......-..-........-.-.......E.........-......................
a_L1PA2_100_93  ...-....*.........................R..L.K......-..-........-.-.................-......................
a_L1PA2_101_94  ...-..........................................-..-........-.-.................-D............E........
a_L1PA2_102_95  ....-..T.................K....................-..-........-.-.................-......................
a_L1PA2_103_96  ...-..........................................-..-........-.-.................-......................
a_L1PA2_104_97  ...-..........................................-..-........-.-.................-...................G..
a_L1PA2_105_98  ...-..........................................-..-........-.-...........D.....-..........T...........
a_L1PA2_106_99  ...-..........................................-..-........-.-.................-....................G.
a_L1PA2_107_100 ...-..................*.................K.....-..-........-.-.................-......................
a_L1PA2_108_101 ...-..........................................-..-........-.-.................-......................
a_L1PA2_109_102 ...-.......C..................................-D.-.....V..-.-.......V.........-......................
a_L1PA2_110_103 ...-..........................................-..-........-.-.....I...........-......................
a_L1PA2_111_104 ...-..........................................-..-........-.-.................-......................
a_L1PA2_112_105 ...-..........................................-..-........-.-.................-............Q.R.......
a_L1PA2_113_106 ...-.......................K..................-..-........-.-.....L...........-.R....................
a_L1PA2_114_107 ...-.............................E....Q.......-..-........-.-..Q.GX...K.......-...................C..
a_L1PA2_116_108 ...-................K.........................-..-........-.-.................-......................
a_L1PA2_117_109 ....-.E......A........D......S.............A..-.Q-....G...-.-.......V.........-.E....................
a_L1PA2_118_110 ...-................K.........................-..-........-.-.................-....K.................
a_L1PA2_119_111 ...-...............................*..........-..-........-.-.....A.V.........-............Q.........
a_L1PA2_120_112 ...-................S......................I..-.P-D......X-.-.................-......................
a_L1PA2_121_113 ....-.X...........Q.........L..I..............-..-...G....-.-..............*..-......................
a_L1PA2_123_114 ...-...................D.....I.......M........-.V-........-R-...............T.-......E...............
a_L1PA2_124_115 ...-................F.................V..P..*.-..-........-.-.................-......................
a_L1PA2_125_116 ....-.E...N....KS...K...W......A..S..D.....I..-..-D....HS.-.-.......V.........-.E.....S....N....D....
a_L1PA2_126_117 ...-......................I...........V.......-..-.......G-.-.......M..N......-......................
a_L1PA2_127_118 ...P-........X................................-..-........-.-.................-......................
a_L1PA2_128_119 ...-.........D...............N................-..-.*......-.-.......V.........-......................
a_L1PA2_131_120 *.K.-KEVR.H....K..L.K.DKWL.....A..S..D........-..-D..T..S.-F-.......V.........-.E....................
a_L1PA2_132_121 ...-............................K............V-K.-........-.-......P..........-..........K....I......
```

```
1.2_o_50%_cn    YSELREDIQTKGKEVENFEKNLEECITRITNTEKCLKELMELKTKARELREECRSLRSRCDQLEERVSAMEDEMNEMKREGKFREKRIKRNEQSLQEIWDY
                ------^------^------^------^------^----+++--^------^------^------^------^------^------^------^------^
a_L1PA2_134_122 ....-.........................................-..-........-.-.................-........L.............
a_L1PA2_135_123 ....-.........................................-..-........-.-..K....V.........-...........K..........
a_L1PA2_136_124 ....-.............X...........................-..-........-.-........I........-......................
a_L1PA2_137_125 ....-.........................................-..-........-G-.V.....G.........-......................
a_L1PA2_140_126 ....-........Q..................K.............-..-........-.-.................-......................
a_L1PA2_141_127 ....-.E.......................................-..-........-.-.......V.........-......................
a_L1PA2_142_128 ....-.A.......................................-..-........-.-.................-..............G.......
a_L1PA2_143_129 ....-................V........................-..-........-.-.................-....G.................
a_L1PA2_144_130 ....-................V........................-..-........-.-.................-....G.................
a_L1PA2_145_131 ....-................V........................-..-........-.-.................-....G.................
a_L1PA2_146_132 ....-................V........................-..-........-.-.................-....G.................
a_L1PA2_147_133 ....-.........................................-..-........-.-.................-......................
a_L1PA2_148_134 ....-*...................................M....-..-........-.-.................-.....K..T.............
a_L1PA2_149_135 ....-.ET...........T..D....................A.T-..-........-.-.......V.........-.....D................
a_L1PA2_150_136 ....-............................R............-D.-........-.-.................-*.....................
a_L1PA2_151_137 ....-V.....................KM..I..............-..-.Q.K....-R-.................-......................
a_L1PA2_152_138 ....-.............L.......I...................-..-........-.-......L..........-......................
a_L1PA2_153_139 ....-....S....................................-..-..F.....-.-..........E......-......................
a_L1PA2_154_140 ....-.........................................-..-........-.-.................-...................R.S
a_L1PA2_155_141 ....-................S.....P..................-..-........-.-.................-......................
a_L1PA2_156_142 ....-....N....................I...............-..-........-.-..............G..-......................
a_L1PA2_157_143 ....-.........................................-..-........-.-H................-......................
a_L1PA2_158_144 ....-.......E.................................-..-.......G-.-.................-.....G.AN.X...........
a_L1PA2_160_145 ....-...............................X.........-..-.....H..-R-................T-.V....................
a_L1PA2_161_146 ....-.........................................-..-........-.-.................-............Q.........
a_L1PA2_162_147 ....-K........................................-..-.......R-.-...............I.-......................
a_L1PA2_163_148 ....-........K................................-..-........-.-..............X..-......................
a_L1PA2_164_149 ....-........................X.......D........-..-........-.-.................-......R......P........
a_L1PA2_165_150 ....-.........................................-..-........-.-.................-.......G..............
a_L1PA2_166_151 ....-....................V.................N..-..-......K.-.-..........E......-......................
a_L1PA2_167_152 ....-...............T.........................-..-........-.-.................-.......G..K.Q.........
a_L1PA2_168_153 ....-.............X...........................-..-........-.-..............G..-......................
a_L1PA2_169_154 ....-...................................D.....-..-........-.-.................-......................
a_L1PA2_170_155 ....-................V........................-..-........-.-.................-....K.................
a_L1PA2_171_156 ....-....S....................................-..-......S.-.-................E-...................*..
a_L1PA2_172_157 ....-.....................S.....*.............-..-.......R-.-.............KK..-............K.........
a_L1PA2_174_158 ....-.............A...........................-..-........-.-................R-......................
a_L1PA2_176_159 ....-...................G..............T......-..-........-.-.................-......................
a_L1PA2_177_160 ....-.........................................-..-........-.-.................-.......*.............C
a_L1PA2_178_161 ....-.........................................-..-........-.-...D.............-......................
a_L1PA2_179_162 ....-.........................................-..-........-.-.................-..........T...........
a_L1PA2_180_163 ....-.N....S..........K..M....................-..-........-.-.......V.........-..N....I..............
a_L1PA2_181_164 ....-....N....................................-..-........-.-........I........-......................
a_L1PA2_182_165 ....-.........................................-..-........-.-.................-......................
a_L1PA2_183_166 ....-.........................................-..-........-.-.Q...............-......................
a_L1PA2_184_167 ....-.........................................-..-........-.-.................-D.....................
a_L1PA2_185_168 ..A.-............S............................-..-........-.-.................-......................
a_L1PA2_188_169 X...-...............K....V....................-..-........-.-.................-......................
a_L1PA2_189_170 C...-..............N.......G.S................-..-........-Y-...........K.....-.....................H
a_L1PA2_190_171 ....-.........................................-..-........-.-.................-.....................C
a_L1PA2_191_172 ....-..................D.M....................-..-........-.-.................-......................
a_L1PA2_192_173 ....-...................R.....................-..-........-.-.................-......................
a_L1PA2_193_174 ....-.........................................-..-........-.-.................-......................
a_L1PA2_194_175 ....-...................................V.....-..-.......N-.-.................-...............R......
a_L1PA2_195_176 ....-...............H............*............-..-........-.-.................-.................A....
a_L1PA2_196_177 ....-........................S..GN............-..-........-.-.................-...................*..
a_L1PA2_197_178 ....-.....................S..S................-..-........-.-............T....-......................
a_L1PA2_198_179 ....-Q........................................-..-........-.-....K............-......................
a_L1PA2_199_180 C...-.........................................-..-........-.-.................-......................
a_L1PA2_201_181 ....-.........................................-..-........-.-.................-..................T.Y.
a_L1PA2_202_182 N...-...................Y......I.R............-..-........-.-.................-......................
a_L1PA2_203_183 ....-..........................R..............-..-........-.-...G.I...........-..............N.......
a_L1PA2_204_184 ....-.......................V.................-..-........-.-.................-......................
a_L1PA2_205_185 ....-...............S.........................-..-........-.-...K.............-......................
```

```
1.2_o_50%_cn    YSELREDIQTKGKEVENFEKNLEECITRITNTEKCLKELMELKTKARELREECRSLRSRCDQLEERVSAMEDEMNEMKREGKFREKRIKRNEQSLQEIWDY
                ------^------^------^------^------^----+++--^------^------^------^------^------^------^------^------^
a_L1PA2_206_186 ....-.............X...........................-..-........-.-.......V.........-......................
a_L1PA2_207_187 ....-.................D.......................-..-........-*-.................-......................
a_L1PA2_208_188 ....-.........................................-..-..Y.....-.-.................-......................
a_L1PA2_209_189 ....-.G.............D.........................-..-..R.....-.-.......V.........-.........Q...*........
a_L1PA2_210_190 ....-......D.............M....Y...............-..-........-.-.................-.........Q............
a_L1PA2_211_191 ....-......D.............................Q....-..-........-.-....S............-......................
a_L1PA2_212_192 ....-.........................................-K.-........-.-....T...T........-......................
a_L1PA2_213_193 ....-.........................................-..-......K.-.-....S............-............D.........
a_L1PA2_214_194 ....-............X.......V...........K........-..-D.......-.-..K.......E......-......................
a_L1PA2_216_195 ....-..V..........X.....................Q.....-..-........-.-.......V.........-......................
a_L1PA2_217_196 ....-.......................V.................-..-........-.-L................-......................
a_L1PA2_219_197 .F..-.........................................-..-........-.-....K............-......................
a_L1PA2_220_198 ....-.................................P.....N.-..-........-.-.................-......................
a_L1PA2_221_199 ..D.-.........................................-..-........-.-.................-......................
a_L1PA2_222_200 ....-...........S.Q..F..*L...................P-..-........-.-H...S............-......................
a_L1PA2_223_201 ....-...E.............................V.......-..-........-.-...K.............-..X..G................
a_L1PA2_224_202 ....-.....E.......K..............R............-..-.......T-.-......T..........-..............N...M...
a_L1PA2_225_203 ....-.........................................-..-........-.-.................-D.....................
a_L1PA2_226_204 .T..-......................................A..-..-........-.-.................-.....D................
a_L1PA2_227_205 ....-.........................................-..-........-.-...V.............-............K.........
a_L1PA2_228_206 ....-......................................A..-..-..R.....-.-.......V.........-..............N.......
a_L1PA2_229_207 S...-...............H..................K......-..-........-.-.................-......................
a_L1PA2_230_208 ....-..........................R..............-..-........-.-.................-......................
a_L1PA2_231_209 ...V-........*................................-..-........-.-.................-......................
a_L1PA2_232_210 ....-.........................................-..-........-.-.................-......................
a_L1PA2_233_211 ....-.........................................-..-........-.-.................-......................
a_L1PA2_234_212 ....-...*....................I.R..............-..-........-.-.................-......................
a_L1PA2_235_213 ....-.........................................-..-........-.-.................-......................
a_L1PA2_236_214 ....-.....................S...................-..-........-.-.................-......................
a_L1PA2_237_215 ....-........................S................-..-........-.-.................-.................G....
a_L1PA2_238_216 ....-........................................T-..-........-.-..........E......-......................
a_L1PA2_239_217 ....-.........................................-..-........-.-.................-......................
a_L1PA2_240_218 ....-............V......W.....................-..-........-.-.................-............G.........
a_L1PA2_241_219 ....-...R.....................................-..-........-.-.................-.A....................
a_L1PA2_242_220 ....-..V...........................V..........-L.-........-.-.................-......................
a_L1PA2_243_221 ....-................................G........-..-........-.-.................-..................V...
a_L1PA2_244_222 ....-.........................T............I..-..-........-.-...............T.-......................
a_L1PA2_246_223 ....-..............R..........................-..-........-.-.................-......................
a_L1PA2_247_224 ....-...................................T.........-..-..Y....G-.-.................-...................*..
a_L1PA2_248_225 ....-....................I....................-..-........-.-.................-.........R............
a_L1PA2_250_226 ....-........................................T-..-........-.-.................-......N...............
a_L1PA2_251_227 ....-..................X....X.................-..-........-.-.....I...........-......................
a_L1PA2_252_228 ....-.........................................-..-........-.-.................-.....K................
a_L1PA2_253_229 ....-...................................K...NT-..-........-H-.................-.......I..............
a_L1PA2_254_230 ....-.......................................I......-..-........-.-.................-..........K...........
a_L1PA2_255_231 ....-.........................................-..-........-.-.................-..........G...........
a_L1PA2_256_232 ....-.................................N....V.......-..-........-.-........T........-......................
a_L1PA2_257_233 ....-....................I..................T......-..-........-.-.................-.............LH.......
a_L1PA2_258_234 ....-.........................................-..-........-.-.................-......................
a_L1PA2_259_235 ....-.........................................-..-........-.-.......V.........-............Q.........
a_L1PA2_260_236 ....-...*..............D......................-..-K.......-.-.................-..................V...
a_L1PA2_261_237 ....-.................K.......................-..-........-.-.................-......................
a_L1PA2_262_238 ....-........X....K....................X......-..-........-.-.................-...................R..
a_L1PA2_263_239 ....-............I................*...........-..-........-.-.................-......................
a_L1PA2_264_240 ....-...................FV.............I......-..-........-.-.................-......................
a_L1PA2_266_241 ....-...................Y.....................-..-........-.-.....I...........-......................
a_L1PA2_267_242 ....-.........................................-..-........-.-..Q..............-......N...............
a_L1PA2_268_243 ....-...............................I.........-..-........-.-.................-.......I..............
a_L1PA2_269_244 ....-..................................G...........-..-.......G-.-.................-D......T......N.......
a_L1PA2_270_245 ....-............I.....................T......-..-........-.-.......V.........-......................
a_L1PA2_271_246 ....-...................G.....................-..-........-.-.......V.........-......................
a_L1PA2_272_247 .Y..-..........Q................Q.*...........-..-........-.-.................-......................
a_L1PA2_273_248 ....-...................Y..S.I......N.........-..-........-.-.................-.......G............N.
a_L1PA2_275_249 ....-..V......................D...............-..-........-.-.................-.R....................
```

```
1.2_o_50%_cn    YSELREDIQTKGKEVENFEKNLEECITRITNTEKCLKELMELKTKARELREECRSLRSRCDQLEERVSAMEDEMNEMKREGKFREKRIKRNEQSLQEIWDY
                ------^------^------^------^------^----+++--^------^------^------^------^------^------^------^------^
a_L1PA2_276_250 ....-.........................................-..-........-.-.................-......................
a_L1PA2_277_251 ....-........Q..........................G..I..-..-........-.-.................-......................
a_L1PA2_278_252 ....-........................P.......Q........-..-........-.-.................-...................*..
a_L1PA2_279_253 ....-................................V........-..-........-.-.................-......................
a_L1PA2_280_254 ....-.........................................-..-........-.-.......V.........-......................
a_L1PA2_281_255 ....-........G............N...................-..-........-.-.................-......................
a_L1PA2_282_256 ....-.N.......................................-..-........-.-.................-....T.........I.......
a_L1PA2_283_257 ....-.......................K.K...............-..-........-.-.................-......................
a_L1PA2_284_258 ....-.........................................-..-........-R-..K.S............-...................*..
a_L1PA2_285_259 ....-.......................M.................-..-........-.-.............S...-.............E........
a_L1PA2_286_260 ..V.-................S..S.....................-..-........-.-.......V.........-......................
a_L1PA2_287_261 ....-.........................................-..-........-.-.................-......................
a_L1PA2_288_262 ....-............X............................-..-........-R-.................-......................
a_L1PA2_289_263 ..A.-..................................T......-..-........-.-.................-.A....................
a_L1PA2_290_264 ....-.........................................-..-........-.-.................-......................
a_L1PA2_291_265 ....-.............K...........................-..-V.......-.-.................-......................
a_L1PA2_293_266 ....-.........................................-..-........-.-.................-.....................H
a_L1PA2_294_267 ....-.........................................-..-........-.-.P...............-......................
a_L1PA2_295_268 ....-.........................................-..-..R.....-.-............I....-..........I...........
a_L1PA2_296_269 ....-............L............................-..-........-.-.......V.........-......................
a_L1PA2_297_270 ....-............V............................-..-........-.-.................-..........G..R........
a_L1PA2_298_271 ....-...................G.....................-..-........-R-..............K..-.......I..............
a_L1PA2_299_272 ....-.............G...........................-..-........-.-.................-..........T...........
a_L1PA2_300_273 ....-......D........K.........................-..-........-.-.................-Q.....................
a_L1PA2_303_274 ....-...................Y.....................-..-........-.-.................-............K.........
a_L1PA2_304_275 ....-..............R..........................-..-........-.-.................-......................
a_L1PA2_305_276 ....-...........................Q.............-..-........-.-.................-......................
a_L1PA2_306_277 ....-........Q..................G.............-..-........-.-.................-..........G.QK........
a_L1PA2_307_278 ....-.........................................-..-........-*-.....I...........-......................
a_L1PA2_308_279 ....-............SX...........................-..-........-.-....T............-......................
a_L1PA2_309_280 ....-.........................................-..-........-.-.................-......................
a_L1PA2_311_281 ....-.........................................-..-..R.....-.-.................-......................
a_L1PA2_312_282 ....-.............................S...........-..-K.......-.-.................-......................
a_L1PA2_313_283 ....-.........................................-..-........-.-.................-......................
a_L1PA2_314_284 ....-.........................................-..-........-.-............I....-......................
a_L1PA2_317_285 ....-.........................................-..-........-.-.................-......................
a_L1PA2_319_286 ....-...R...............Y.....................-..-........-.-.................-......................
a_L1PA2_320_287 ....-............S............................-..-........-.-.................-.....X.K..X...........
a_L1PA2_321_288 ....-.........................................-..-........-.-...............K.-.........E............
a_L1PA2_322_289 ..K.-......................T..................-..-........-.-.................-....G.....K...........
a_L1PA2_324_290 ....-.........................................-..-........-.-.................-......................
a_L1PA2_325_291 ....-.........................................-..-....R...-S-E................-....T.................
a_L1PA2_327_292 ....-......................................S..-..-........-.-.................-......................
a_L1PA2_329_293 ....-.....................I...................-..-........-.-.................-......................
a_L1PA2_330_294 ....-.................AV.........N............-..-........-.-.................-....................G.
a_L1PA2_331_295 ....-...E.....................................-..-........-.-.................-......................
a_L1PA2_332_296 ....-.........................................-..-........-S-.................-...................*..
a_L1PA2_334_297 ....-...................Y.....................-..-........-.-.V...............-....................N.
a_L1PA2_335_298 ....-.G............N...............S..........-..-...*....-.-.................-......................
a_L1PA2_336_299 ....-....................T....................-..-........-.-.................-..........X...........
a_L1PA2_339_300 ....-...................Y.....................-..-........-.-.................-......................
a_L1PA2_340_301 ....-..V.............V........................-..-......G.-.-.................-......................
a_L1PA2_341_302 ....-.E..................................Q....-..-........-.-.................-..N...................
a_L1PA2_342_303 ....-.........................................-..-........-.-.......G.........-......................
a_L1PA2_343_304 ....-..........................R..............-..-........-.-.................-......................
a_L1PA2_344_305 ....-..................................T......-..-........-.-.................-......................
a_L1PA2_345_306 ....-.........................................-..-........-.-.................-...........K..........
a_L1PA2_346_307 ....-............L........I...............N...-.Q-........-R-.................-..........G.........G.
a_L1PA2_347_308 ....-.....................................Q...-..-........-.-.................-......................
a_L1PA2_348_309 ....-.........................S...............-..-........-.-.............S...-.........N............
a_L1PA2_349_310 ....-.........................................-..-........-.-.................-......................
a_L1PA2_350_311 ....-.................G.......................-..-........-.-.................-......................
a_L1PA2_352_312 ....-.........................................-..-..W.....-.-.................-......................
a_L1PA2_353_313 ....-.........................................-.I-........-.-.......V.........-.....X................
```

```
1.2_o_50%_cn     YSELREDIQTKGKEVENFEKNLEECITRITNTEKCLKELMELKTKARELREECRSLRSRCDQLEERVSAMEDEMNEMKREGKFREKRIKRNEQSLQEIWDY
                 ------^------^------^------^------^----+++--^------^------^------^------^------^------^------^------^
a_L1PA2_356_314  ....-......V..................................-..-........-.-.................-......................
a_L1PA2_357_315  ....-................................D........-..-........-.-.................-......................
a_L1PA2_358_316  ....-...................Y.....................-..-........-.-.................-.......T..............
a_L1PA2_359_317  ....-......................T..................-..-........-.-.................-....T.................
a_L1PA2_360_318  ....-.................D..T....................-..-........-.-.................-.................D....
a_L1PA2_362_319  ....-......................K..................-..-........-.-.................-............K.X.......
a_L1PA2_364_320  ....-.........................................-..-........-.-.......G.........-......................
a_L1PA2_365_321  ....-......V........K........I................-..-.......N-.-.......V.........-......................
a_L1PA2_366_322  ....-.........................................-..-........-.-.....I...........-......................
a_L1PA2_367_323  ....-.........................................-..-........-.-.................-...................*..
a_L1PA2_368_324  ....-.........................................-..-........-.-..........E..S...-...........T..........
a_L1PA2_369_325  ....-..........*.Y............................-..-.K......-.-.......V.........-...................R..
a_L1PA2_370_326  ....-.........................................-..-........-.-.................-......................
a_L1PA2_371_327  ....-.........................................-..-........-.-.......V.........-......................
a_L1PA2_372_328  ....-.........................................-..-........-Y-.............K...-.....K................
a_L1PA2_373_329  ....-.........................................-..-........-.-.................-......................
a_L1PA2_376_330  ....-.........................................-..-........-.-.......V....IK...-......................
a_L1PA2_377_331  ....-.................................P.......-..-....N...-.-.................-......................
a_L1PA2_378_332  ....-....................................Q....-..-........-.-.................-......................
a_L1PA2_379_333  ....-..........................P..............-..-........-.-.................-......................
a_L1PA2_380_334  ....-.........................................-..-........-.-.................-......................
a_L1PA2_382_335  ....-.........................................-..-........-.-......T..........-......................
a_L1PA2_383_336  ....-..........................K............N.-..-........-.-..........G......-......................
a_L1PA2_385_337  ....-........*..............R.................-..-........-.-.................-......................
a_L1PA2_386_338  ....-.........................................-..-........-.-.................-.......G.............C
a_L1PA2_387_339  ....-.........................................-..-........-.-.M..........I....-...................*..
a_L1PA2_388_340  ....-........A........G.......................-..-........-.-.................-......................
a_L1PA2_391_341  ....-.........................................-..-........-.-.................-......................
a_L1PA2_392_342  ....-.........................................-..-........-.-.................-......................
a_L1PA2_393_343  ....-.........................................-..-........-.-..*..............-......................
a_L1PA2_394_344  ....-.........................................-..-........-.-.......V.........-......................
a_L1PA2_395_345  ....-.........................................-..-........-.-.................-......................
a_L1PA2_396_346  ....-..................Q...........S..........-..-........-.-.....X.E.........-......................
a_L1PA2_397_347  ....-.........................................-..-........-.-.................-......................
a_L1PA2_398_348  .P..-..........G..............................-..-........-.-.......V.........-......................
a_L1PA2_399_349  ....-.........................................-..-........-.-.................-......................
a_L1PA2_400_350  .P..-........................N........V.......-..-....R...-.-.................-......................
a_L1PA2_401_351  ....-........................................T-..-........-.-.................-.......I..............
a_L1PA2_402_352  ....-.........................................-..-........-.-.......T.........-...................C..
a_L1PA2_403_353  ....-.........................................-..-........-.-............R....-......................
a_L1PA2_404_354  ....-.........................................-..-........-.-.................-......................
a_L1PA2_405_355  ....-.........................................-..-........-.-.................-......................
a_L1PA2_406_356  ....-........................IK...............-..-........-.-.................-......................
a_L1PA2_407_357  ....-.........................................-..-........-.-.................-..................M...
a_L1PA2_408_358  ....-..................K......................-..-......G.-.-.................-......................
a_L1PA2_409_359  ....-.........................................-..-........-.-.................-......................
a_L1PA2_410_360  ....-.............X...........................-..-........-.-.................-......................
a_L1PA2_411_361  ....-.........................K...............-.I-........-.-.................-......................
a_L1PA2_412_362  ....-..................................I......-..-........-.-.................-......................
a_L1PA2_413_363  ....-.........................................-..-........-.-..............Q..-......................
a_L1PA2_414_364  ....-.........................................-..-........-.-.................-......................
a_L1PA2_415_365  ....-.........................................-..-*.......-.-.................-......................
a_L1PA2_416_366  ....-.........................................-..-........-.-.................-......................
a_L1PA2_417_367  ....-.........................................-..-..Y.....-.-.................-..T.............K.....
b_L1PA2_2_368    ....-K...............S...T........F...........-..-........-.-...............R.-.........E............
b_L1PA2_4_369    ....-.........................................-..-........-.-.......V.........-......................
b_L1PA2_5_370    ....-.........................................-..-........-.-.......V...*.....-......................
b_L1PA2_6_371    ....-.........................................-..-........-*-.................-..................M...
b_L1PA2_8_372    ....-.........................................-..-........-.-.............K...-........M....R........
b_L1PA2_9_373    ....-.........................................-..-........-.-.................-....K.................
b_L1PA2_11_374   .P..-.........................................-..-........-.-.................-......................
b_L1PA2_12_375   D...-..........K..............................-..-........-.-.................-......................
b_L1PA2_13_376   ....-......R..................................-..-........-.-.................-......................
b_L1PA2_14_377   ....-.........................................-..-........-.-.P...............-.....................C
```

```
1.2_o_50%_cn    YSELREDIQTKGKEVENFEKNLEECITRITNTEKCLKELMELKTKARELREECRSLRSRCDQLEERVSAMEDEMNEMKREGKFREKRIKRNEQSLQEIWDY
                ------^------^------^------^------^----+++--^------^------^------^------^------^------^------^------^
b_L1PA2_17_378  ....-..................................F-X........-..-........-.-.................-......................
b_L1PA2_19_379  ....-.........................................-..-........-.-.................-......................
b_L1PA2_21_380  ....-.........................................-..-........-.-...............K.-......................
b_L1PA2_23_381  ....-.......................R.................-..-........-*-...D.............-.....X.K..............
b_L1PA2_24_382  ....-.........................................-..-........-.-.................-......................
b_L1PA2_29_383  ....-.......R.................................-..-........-.-.......V.........-....T..M..............
b_L1PA2_32_384  ....-.........................................-..-........-.-.................-......................
b_L1PA2_33_385  ....-.........................................-..-........-.-.................-......................
b_L1PA2_34_386  ....-...................Y.....................-..-........-.-.................-......................
b_L1PA2_35_387  ....-.........................................-..-.......R-.-.................-..............T.......
b_L1PA2_38_388  ....-.........................................-..-........-.-.................-......................
b_L1PA2_44_389  ....-....................L....................-..-........-.-.................-......................
b_L1PA2_45_390  ....-...................Y......A..............-..-........-W-.......V.........-......................
b_L1PA2_46_391  ....-..............N..........................-..-........-.-.................-......................
b_L1PA2_47_392  ....-.........................................-..-........-.-.................-......................
b_L1PA2_48_393  ....-..................K......................-..-........-.-.................-......................
b_L1PA2_49_394  ....-.....................N..I...............S-..-........-.-.................-......................
b_L1PA2_50_395  ....-............V............................-..-........-.-.................-.........R............
b_L1PA2_51_396  ..A.-...................................G.....-..-........-.-.................-......................
b_L1PA2_52_397  ....-..........K..............................-..-........-.-.................-......................
b_L1PA2_53_398  ....-..........................K..............-..-........-.-.................-..........K..........H
b_L1PA2_54_399  ....-.........................................-..-........-.-.................-......................
b_L1PA2_55_400  ....-.................Q..................M....-..-..H.....-.-...G........I....-......................
b_L1PA2_56_401  ..T.-........................S................-..-........-.-.................-......................
b_L1PA2_57_402  ....-...........KL............................-..-........-.-.................-......................
b_L1PA2_58_403  .F..-...............S.........................-..-........-.-.................-......................
b_L1PA2_59_404  ....-...........H.............................-..-........-.-.................-......................
b_L1PA2_60_405  ....-.........................................-..-........-.-.................-......................
b_L1PA2_61_406  ....-...................G..............T......-..-........-.-.........G.......-......................
b_L1PA2_62_407  ....-..............Q..........................-..-........-.-.....I*..........-..............N.......
b_L1PA2_63_408  ....-.........................................-..-........-.-.................-*.................G..
b_L1PA2_64_409  ....-.....................................P...-..-........-.-.................-......................
b_L1PA2_66_410  ....-.........................................-..-........-.-..............A..-............G.........
b_L1PA2_67_411  ....-.........................................-..-........-.-............T....-.........T............
b_L1PA2_68_412  ..Q.-.........................................-..-........-.-.................-......................
b_L1PA2_69_413  ....-D...I........G............I..............-..-........-.-............V....-..................V...
b_L1PA2_70_414  ...I-.........................................-..-........-.-.................-......................
b_L1PA2_71_415  ....-.........................................-..-........-.-....M............-......................
b_L1PA2_72_416  ....-.....................................V...-..-........-.-.................-............Q.........
b_L1PA2_73_417  ....-.........................................-..-........-.-.................-......................
b_L1PA2_74_418  ....-............X............................-..-........-.-.................-......................
b_L1PA2_75_419  ....-.............X...........................-..-........-.-.................-.............K........
b_L1PA2_76_420  ....-...............I.........................-..-........-.-............R....-......................
b_L1PA2_77_421  ....-.........................................-..-........-.-.................-......................
b_L1PA2_78_422  ....-..F................Y.....................-..-........-.-.................-......................
b_L1PA2_79_423  ...I-.................*............S.........-..-........-.-.................-......................
b_L1PA2_80_424  ....-........................N................-..-........-.-.................-......................
b_L1PA2_81_425  ....-...K.....L...............................-..-........-.-.................-......................
b_L1PA2_82_426  ....-.........................................-..-........-.-.................-......................
b_L1PA2_85_427  ....-...............K.........................-..-........-.-....K............-......................
b_L1PA2_86_428  ....-.......................................X.-..-........-.-.................-......................
b_L1PA2_87_429  ....-.........................................-..-........-.-.................-......................
b_L1PA2_88_430  ....-...................R.....................-..-........-.-.................-......................
b_L1PA2_89_431  ....-................*........................-D.-........-.-.................-.............X........
b_L1PA2_90_432  ....-.........................................-..-........-.-*.............*..-.....R................
b_L1PA2_91_433  ....-........................A................-..-........-.-.................-................P.V...
b_L1PA2_92_434  ....-.........................................-..-........-.-.................-..................V...
b_L1PA2_93_435  ....-.........................................-..-........-.-.................-..................V...
b_L1PA2_94_436  ....-.........................................-..-........-.-.................-.....................H
b_L1PA2_95_437  ....-....S....................................-..-........-.-.................-......................
b_L1PA2_96_438  ....-.......N.................................-..-.......R-.-.................-......................
b_L1PA2_97_439  ....-....................................Q....-..-........-.-.................-........V.I...........
b_L1PA2_98_440  ....-.........................................-..-.......G-.-......*..K.......-......................
b_L1PA2_99_441  ....-.....T...................................-..-........-.-........T........-......................
```

```
1.2_o_50%_cn    YSELREDIQTKGKEVENFEKNLEECITRITNTEKCLKELMELKTKARELREECRSLRSRCDQLEERVSAMEDEMNEMKREGKFREKRIKRNEQSLQEIWDY
                ------^------^------^------^------^----+++--^------^------^------^------^------^------^------^------^
b_L1PA2_100_442 ....-.........................................-..-........-Y-.................-.......Q..............
b_L1PA2_101_443 ....-.........................................-..-........-.-.................-...................C..
b_L1PA2_102_444 ....-.........................................-..-........-.-.................-......................
b_L1PA2_103_445 ....-.............Q...........................-..-........-.-.................-......................
b_L1PA2_104_446 ....-.........................................-..-........-.-.................-......................
b_L1PA2_105_447 ....-.........................................-..-.......N-.-.................-......................
b_L1PA2_106_448 ....-..............................*..........-..-........-.-.................-K.....................
b_L1PA2_107_449 ....-.........................................-..-........-.-.................-......................
b_L1PA2_108_450 ....-.........................................-..-........-.-.................-............Q.........
b_L1PA2_109_451 ....-.........................................-..-........-.-.................-......................
b_L1PA2_110_452 ....-.........................................-D.-........-.-.......V.........-......................
b_L1PA2_111_453 ....-.....................................Q...-..-........-.-............I....-......................
b_L1PA2_112_454 ....-........................I................-..-........-.-.................-......................
b_L1PA2_113_455 ....-...............I.........................-..-........-.-............R....-......................
b_L1PA2_114_456 ....-.........................................-..-........-.-.......V.........-......................
b_L1PA2_115_457 ....-.........................................-..-........-.-.......V.........-......................
b_L1PA2_116_458 ....-......................................M..M....-..-........-.-...........QI....-......................
b_L1PA2_117_459 ....-.........................................-..-........-.-.................-......................
b_L1PA2_118_460 ....-K....E...............................Q...-..-........-.-.................-..........T...........
b_L1PA2_119_461 ....-.................A.......................-..-........-F-............I....-......................
b_L1PA2_120_462 ..Q.-.........................................-..-........-.-.................-......................
b_L1PA2_121_463 ....-...................................K.....-..-.......R-.-.......V.........-......................
b_L1PA2_122_464 ....-.........................................-..-........-.-.................-..N...................
b_L1PA2_126_465 ....-.........................................-..-........-.-.................-......................
b_L1PA2_127_466 ....-.....................................K........-..-........-.-.................-......................
b_L1PA2_128_467 ....-.........................................-..-........-.-.................-......................
b_L1PA2_129_468 ...V-.........................................-..-........-.-.................-......................
b_L1PA2_130_469 ....-..........K..............................-..-........-.-.................-......................
b_L1PA2_131_470 ....-.........................................-..-........-.-.................-......................
b_L1PA2_135_471 ....-.........................................-.I-........-.-.................-..........SS..........
b_L1PA2_141_472 ....-K........................................-..-........-.-.................-......................
b_L1PA2_142_473 ....-.............X...........................-..-........-.-....G............-......................
b_L1PA2_143_474 ....-.........................................-..-........-.-.................-......................
b_L1PA2_144_475 ....-..........K...................V..........-..-........-R-.................-......................
b_L1PA2_145_476 ....-.........................................-..-........-.-.................-......................
b_L1PA2_146_477 ....-.......................G..........R.K.....-..-........-.-.................-....................N.
b_L1PA2_147_478 ....-.......................G..........R.K.....-..-........-.-.................-....................N.
b_L1PA2_148_479 ....-.N.................................Q.....-..-...K...N-.-.................-............D......*..
b_L1PA2_149_480 ....-.........................................-..-........-.-.................-........M.............
b_L1PA2_150_481 ....-.......................*..............V.......-..-...K....-.-.................-......................
b_L1PA2_151_482 ....-.........................................-..-........-.-.......V.........-.V......M.G........*..
b_L1PA2_152_483 ....-...............Y...Y.....................-..-..S.....-R-.................-......................
b_L1PA2_153_484 .F.-..T.......................................-..-........-.-.................-......................
b_L1PA2_154_485 ....-.E...........................S........A..-..-........-.-.......V.........-............Q.........
b_L1PA2_155_486 ....-........................Y.....................-..-........-.-.................-......................
b_L1PA2_157_487 ....-.........................................-..-........-.-..G..............-.......T..............
b_L1PA2_158_488 ....-.........................................-..-........-.-.................-..........T...........
b_L1PA2_159_489 ....-.E.........H....I..................K.....-..-........-.-..............G..-.........Q..K.........
b_L1PA2_160_490 ...P-...........................K.............-..-........-Y-..............D..-......................
b_L1PA2_161_491 ....-.........................................-..-........-.-...............X.-......................
b_L1PA2_162_492 ....-........................................V-..-........-.-.....I.V.........-.......T........H...Y.
b_L1PA2_163_493 ....-.........................................-..-........-.-.......V.........-......................
b_L1PA2_164_494 ....-.........................................-..-.......R-.-.................-......................
b_L1PA2_165_495 ....-.........................................-..-........-.-.................-......................
b_L1PA2_166_496 ....-.........................................-..-........-.-.................-.......S.R........V...
b_L1PA2_167_497 ....-.E.......................................-..-........-.-.................-......................
b_L1PA2_168_498 ....-.........................................-..-........-*-.................-..................V...
b_L1PA2_169_499 ....-............S.......T....................-..-......S.-.-.......VI..Q.....-......................
b_L1PA2_170_500 ....-.........................................-..-........-.-........I........-....G.................
b_L1PA2_172_501 ....-.................*.......................-..-........-.-.................-......................
b_L1PA2_173_502 ....-.........GG..................G...........-..-.....H..-.-.................-......................
b_L1PA2_174_503 ....-D...I........G............I..............-..-........-W-.................-.....X................
b_L1PA2_175_504 ....-..........K..............................-..-..R.....-.-..........E......-............K.........
b_L1PA2_176_505 ....-.............X...........................-..-........-.-.................-......................
```

```
1.2_o_50%_cn     YSELREDIQTKGKEVENFEKNLEECITRITNTEKCLKELMELKTKARELREECRSLRSRCDQLEERVSAMEDEMNEMKREGKFREKRIKRNEQSLQEIWDY
                 ------^------^------^------^------^----+++--^------^------^------^------^------^------^------^------^
b_L1PA2_178_506  ....-.........................................-..-........-.-..................-.....................
b_L1PA2_179_507  ....-................V.......I....R...........-..-........-.-..................-.....................
b_L1PA2_180_508  ....-..........................I..............-..-........-.-..................-.....................
b_L1PA2_181_509  ....-.........................................-..-........-.-..................-........T.........G..
b_L1PA2_182_510  ....-..................G......................-..-........-R-.............S....-.....................
b_L1PA2_183_511  .Y..-..T......................................-..-........-.-.......V..........-.....................
b_L1PA2_184_512  ....-.........................................-..-........-.-..................-.......K.............
b_L1PA2_185_513  ....-.........................................-..-........-.-..................-.....................
b_L1PA2_186_514  ....-.........L...............................-D.-........-.-..................-.....................
b_L1PA2_187_515  ....-.......................................R.-..-........-.-..................-.................L...
b_L1PA2_188_516  ....-...........T.............................-..-........-.-..................-....D................
b_L1PA2_189_517  ....-.................D........R..............-..-........-.-..................-.....................
b_L1PA2_190_518  ....-..............R......S...................-..-........-.-..................-.....................
b_L1PA2_191_519  ....-...............K.........................-..-........-.-..................-.....................
b_L1PA2_192_520  ....-.........................................-..-........-.-..................-.....................
b_L1PA2_193_521  ....-........*.D...........................I..-..-........-.-..................-.....................
b_L1PA2_194_522  ....-.........................................-..-........-.-..................-...........K.........
b_L1PA2_195_523  ....-.................D.......................-..-........-.-..................-.....................
b_L1PA2_196_524  ....-.........................................-..-........-.-....T...V.........-..............F..L...
b_L1PA2_197_525  ....-...................Y.....................-..-........-.-.....I............-.....................
b_L1PA2_198_526  ....-.....E........................F..........-.Q-........-.-...Q...V....I.....-.....................
b_L1PA2_199_527  ....-................................L.L......-..-..R.....-.-..................-.....................
b_L1PA2_200_528  ....-.........................................-..-........-.-.....L............-.....................
b_L1PA2_201_529  ....-..................D......................-..-...*....-.-............V.....-.....................
b_L1PA2_202_530  ....-..T................................Q.....-..-........-.-..................-...........K.........
b_L1PA2_203_531  ....-.........................................-..-........-.-..................-*.................G..
b_L1PA2_204_532  ....-.............Q...........................-..-........-.-..................-.....................
b_L1PA2_205_533  ....-........K................................-..-........-.-..................-.....................
b_L1PA2_206_534  ....-K........................................-..-........-.-...........K......-K...................C
b_L1PA2_207_535  ....-......................................P..-..-........-.-............T.....-.....................
b_L1PA2_208_536  ....-.............................N...........-..-.......R-.-..................-.....................
b_L1PA2_209_537  ....-......D.................S.......Q.......V-..-........-.-....K..V..........-.....................
b_L1PA2_210_538  ....-............X............................-..-.......R-.-..................-..................R..
b_L1PA2_211_539  ....-.........................................-..-........-.-.....I............-.....................
b_L1PA2_212_540  ..D.-..TR.............A.....T.................-..-........-*-..................-.....................
b_L1PA2_213_541  ....-..........K.....*............Y...........-..-........-.-..................-.....................
b_L1PA2_214_542  ....-.................D.......SI..............-..-........-.-..................-.....................
b_L1PA2_215_543  X...-......C.*................................-..-........-.-..................-....D................
b_L1PA2_216_544  ....-.........................................-..-........-.-..................-.....................
b_L1PA2_217_545  ....-...............T.........................-..-..G.N..K-.-..................-E....................
b_L1PA2_218_546  ....-.........................................-..-.......R-.-.......V..........-...................G.
b_L1PA2_219_547  ....-.........................V........G......-..-........-.-.......G..........-.....................
b_L1PA2_220_548  ....-.........................................-..-........-.-............I.....-.......T.............
b_L1PA2_221_549  ....-.............................S...........-..-........-.-..................-....................D
b_L1PA2_223_550  ....-.........................................-..-........-.-..................-.....................
b_L1PA2_224_551  ....-.........................................-..-........-.-..................-...................V.
b_L1PA2_226_552  ....-.........................................-..-D.......-.-.......V..........-.....................
b_L1PA2_227_553  ...P-...........T.....................P.......-..-........-.-.....I............-.....................
b_L1PA2_228_554  ....-.........................................-..-........-.-...............I..-...........Q.........
b_L1PA2_229_555  ....-..........................R..............-..-........-.-..................-.....................
b_L1PA2_230_556  ....-..........................RD.............-.P-........-.-......LVV.........-.....................
b_L1PA2_231_557  ....-.........................................-..-........-.-..................-.....................
b_L1PA2_232_558  ....-................*..................Q.....-..-........-.-..................-.....................
b_L1PA2_233_559  ....-.........................................-..-........-.-..................-.....................
b_L1PA2_234_560  ....-.........................................-..-........-.-..................-.....................
b_L1PA2_235_561  ....-.........................................-..-........-.-..................-.....................
b_L1PA2_236_562  ....-....S....................................-..-........-.-..........G.......-.....................
b_L1PA2_237_563  ....-.......................K.................-..-........-.-.......V.......L..-........Q.....F......
b_L1PA2_238_564  ....-........K................................-..-........-.-............K.....-.........KK..........
b_L1PA2_239_565  ....-.........................................-..-........-.-..................-.....................
b_L1PA2_240_566  ....-.........................................-..-....R...-.-..................-......I..............
b_L1PA2_241_567  ....-.........................V...............-..-........-.-..................-...............H.....
b_L1PA2_242_568  ....-...............S.........................-..-........-.-..................-.....................
b_L1PA2_243_569  ....-.........................................-..-....R...-.-..................-.....................
```

```
1.2_o_50%_cn      YSELREDIQTKGKEVENFEKNLEECITRITNTEKCLKELMELKTKARELREECRSLRSRCDQLEERVSAMEDEMNEMKREGKFREKRIKRNEQSLQEIWDY
                  ------^------^------^------^------^----+++--^------^------^------^------^------^------^------^------^
b_L1PA2_244_570   ....-......................................A..-..-........-.-.................-......................
b_L1PA2_245_571   ....-.........................................-..-........-.-.................-......................
b_L1PA2_246_572   ....-.........................................-.V-........-.-.................-......................
b_L1PA2_247_573   ....-..........K..............................-..-........-.-...........Q.....-......................
b_L1PA2_248_574   ....-.........................................-..-........-.-.................-......................
b_L1PA2_249_575   ....-..........K..............................-..-........-.-.......V.........-......................
b_L1PA2_250_576   ....-......X..................................-..-....T...-.-............R....-......................
b_L1PA2_252_577   ....-.........................................-..-........-.-......*..........-......................
b_L1PA2_253_578   ....-.........................................-..-........-.-..........E......-......................
b_L1PA2_254_579   ....-.........................................-..-.....H..-.-.................-.R....................
b_L1PA2_256_580   ....-..............N..........................-..-........-.-.................-......................
b_L1PA2_257_581   ....-D........................................-..-..Y.....-.-........I........-.............*........
b_L1PA2_258_582   ....-.........................................-..-........-.-.................-......................
b_L1PA2_259_583   ....-...................Y.....................-..-.....FS.-F-.................-.................K....
b_L1PA2_260_584   ....-..................D......................-..-........-*-.................-......................
b_L1PA2_261_585   ....-.............................Y...........-..-........-.-.................-......................
b_L1PA2_262_586   ....-............................*............-..-........-.-.................-......................
b_L1PA2_263_587   ....-.........................................-..-........-.-............I....-......................
b_L1PA2_264_588   ....-...................................Q.....-..-........-.-.................-......................
b_L1PA2_265_589   ....-...E..V..................................-..-........-.-.............S...-................E.....
b_L1PA2_266_590   ....-.........................................-..-........-*-.................-......................
b_L1PA2_267_591   ....-...............T.........................-..-........-.-..............K.N-D.......M.............
b_L1PA2_268_592   ....-.A.......................................-..-........-.-......L..........-..........K...........
b_L1PA2_269_593   ....-.........................................-..-........-.-.................-......................
b_L1PA2_270_594   ....-.................D........K..Y...........-..-........-.-.......V.......I.-...........K..........
b_L1PA2_271_595   ....-..............Q.S........................-..-........-.-.................-......................
b_L1PA2_272_596   ....-.........................................-..-........-.-.....A...K.......-......................
b_L1PA2_273_597   ....-..V......................................-..-........-.-.................-.....D................
b_L1PA2_274_598   ....-.........................................-..-........-.-.....L...........-......................
b_L1PA2_275_599   ....-.........................................-..-.......N-.-.................-......................
b_L1PA2_276_600   ....-.........................................-..-........-.-............X-X..-......................
b_L1PA2_277_601   ....-.........................................-..-........-.-.................-.E............T.......
b_L1PA2_278_602   ....-.........................................-..-........-.-.................-......................
b_L1PA2_279_603   ....-..........D............T.................-..-....R...-.-.................-......................
b_L1PA2_280_604   ....-.........................................-..-........-.-.................-........L....*........
b_L1PA2_281_605   ....-........................................T-..-..S.....-.-.................-.........N.........R..
b_L1PA2_282_606   ....-.........................................-..-........-.-.................-......................
b_L1PA2_283_607   ....-...........S.....................V.......-..-........-.-...........*.....-......................
b_L1PA2_284_608   ....-.........................................-..-..H.....-.-....S............-.....................C
b_L1PA2_285_609   ....-......S..................................-..-.....R..-.-.................-......................
b_L1PA2_286_610   ....-..............N..........................-..-..*.....-.-.................-......................
b_L1PA2_287_611   ....-.............................Y...........-..-........-.-.......V.........-...........Y..........
b_L1PA2_288_612   ....-..............N..........................-..-........-.-.................-......................
b_L1PA2_289_613   ....-.........................................-..-........-.-.................-......................
b_L1PA2_290_614   ..D.-.........................................-..-........-W-.................-....................E.
b_L1PA2_291_615   ....-.........................................-..-........-.-.................-......................
b_L1PA2_292_616   ....-...............I.........................-..-........-.-.................-......................
b_L1PA2_293_617   ....-.........................................-..-........-*-.........A.......-......................
b_L1PA2_294_618   ....-.................K.......................-..-........-.-.................-......................
b_L1PA2_295_619   ....-..........G..............................-..-........-.-.................-......E...............
b_L1PA2_296_620   ....-.........................................-..-........-.-........V........-K.....................
b_L1PA2_297_621   ....-.........................................-..-........-.-.................-......................
b_L1PA2_298_622   ....-.........................................-..-........-.-.................-......................
b_L1PA2_299_623   ....-..................................I......-..-........-.-.......T....I....-.....Q................
b_L1PA2_300_624   ....-.........................................-..-K.......-.-.................-.....D................
b_L1PA2_301_625   ....-.N.......................................-..-........-.-.................-.........R.S..........
b_L1PA2_302_626   ..Q.-..................................T......-..-........-.-.................-......................
b_L1PA2_303_627   ....-....A...........F........................-..-........-.-...............I.-......................
b_L1PA2_305_628   ....-.........................................-..-........-.-.................-..................V...
b_L1PA2_306_629   ....-.........................................-..-........-.-.................-..................V...
b_L1PA2_307_630   ....-.........D...............................-..-......S.-.-H................-......................
b_L1PA2_308_631   ....-...................Y.....................-..-........-.-.................-........M..........C..
b_L1PA2_309_632   ..K.-.........................................-..-........-.-.................-......................
b_L1PA2_310_633   ....-.....................I...................-..-........-.-.................-......................
```

```
1.2_o_50%_cn    YSELREDIQTKGKEVENFEKNLEECITRITNTEKCLKELMELKTKARELREECRSLRSRCDQLEERVSAMEDEMNEMKREGKFREKRIKRNEQSLQEIWDY
                ------^------^------^------^------^----+++--^------^------^------^------^------^------^------^------^
b_L1PA2_311_634 ....-.........................................-..-........-.-.................-......................
b_L1PA2_312_635 ....-...............................X.........-..-........-.-.......V.........-.......G..............
b_L1PA2_313_636 ....-.............G...........................-..-........-.-.................-......................
b_L1PA2_314_637 ....-.........................................-..-........-.-.................-......................
b_L1PA2_315_638 ....-.........................................-..-........-*-.P...............-......................
b_L1PA2_316_639 ....-.........................................-..-........-.-.................-.................*.*..
b_L1PA2_317_640 ....-........................I................-..-........-.-.................-......................
b_L1PA2_318_641 ....-..........D..............................-..-........-.-.................-......................
b_L1PA2_319_642 ....-....S............................*.......-..-........-Y-.................-......................
b_L1PA2_320_643 ....-.........................................-..-......G.-.-.................-......................
b_L1PA2_321_644 ....-.........................................-..-........-Y-.................-......................
b_L1PA2_322_645 ....-.......................LI................-..-........-.-.................-......................
b_L1PA2_323_646 ..K.-.........................................-..-........-.-.................-.....D.............R.C
b_L1PA2_324_647 ....-.........................................-..-........-.-........R........-......................
b_L1PA2_325_648 ....-.........................................-..-........-.-........R........-.R....................
b_L1PA2_326_649 ....-.........................................-..-........-.-.....A...........-......................
b_L1PA2_327_650 ....-.........................................-..-........-.-.................-...........T..........
b_L1PA2_328_651 ....-.........L...............................-..-........-.-.................-......................
b_L1PA2_329_652 ....-.................D.......................-..-...T....-.-........T...I....-......................
b_L1PA2_330_653 ....-....S...............K....................-..-........-.-.................-......................
b_L1PA2_331_654 ....-..........................K..............-..-........-.-.....I...........-..N..X.KK.....R.......
b_L1PA2_332_655 ....-................*........................-..-........-.-.................-......................
b_L1PA2_333_656 ....-.............................W...........-..-........-.-.................-......................
b_L1PA2_334_657 ....-...........................G.............-..-........-.-...............I.-......................
b_L1PA2_335_658 ....-.........................................-..-........-.-.................-.....................C
b_L1PA2_336_659 ....-.........................................-..-........-.-.................-..T...................
b_L1PA2_337_660 ....-...........................Q.............-..-........-.-.................-......................
b_L1PA2_338_661 ....-.........................................-D.-........-.-.................-...................*..
b_L1PA2_339_662 ....-.........................................-..-........-.-.................-......................
b_L1PA2_340_663 ....-.........................................-..-........-.-.................-............QK........
b_L1PA2_341_664 ....-.E.......................................-..-........-R-.................-......................
b_L1PA2_343_665 ....-.........A............................P..-..-........-.-.................-......................
b_L1PA2_344_666 ....-.........................................-..-........-.-.................-......................
b_L1PA2_345_667 ....-.........................................-..-........-.-..............V..-............K.........
b_L1PA2_346_668 ....-..................................I......-..-........-.-.................-......................
b_L1PA2_347_669 ....-.............................Y...........-..-........-.-.................-......................
b_L1PA2_348_670 ....-.........................................-..-........-.-.................-......................
b_L1PA2_349_671 ....-.........................................-..-........-.-.................-......................
b_L1PA2_350_672 ....-......................T......Y...........-..-........-.-.........K.......-......................
b_L1PA2_351_673 ....-.........................................-..-........-.-...............X.-...................Q..
b_L1PA2_352_674 ....-.............K...........................-..-......G.-.-.................-......................
b_L1PA2_353_675 ....-.........................................-..-........-.-.................-............G........C
b_L1PA2_354_676 ....-...........D.............................-..-........-.-.................-......................
b_L1PA2_355_677 ....-.........................................-..-.......N-.-.................-......................
b_L1PA2_356_678 ....-..................................T......-..-........-.-.................-......................
b_L1PA2_357_679 ....-.........................................-..-........-.-.......V.........-....G.................
b_L1PA2_358_680 ....-...............S.........................-..-........-.-..*..............-......................
b_L1PA2_359_681 ....-.........................................-..-........-.-.................-......................
b_L1PA2_360_682 ....-...........................G.............-..-........-.-.................-......................
b_L1PA2_361_683 ....-.........................................-..-........-.-.................-......................
b_L1PA2_362_684 ....-...........................K.............-K.-........-.-.................-......................
b_L1PA2_363_685 ....-..................................K......-..-.......C-.-............I....-......................
b_L1PA2_364_686 ....-....S....................................-..-........-.-............T....-......................
b_L1PA2_365_687 ....-.........................................-A.-A.......-*-.................-......................
b_L1PA2_366_688 ....-.........................................-..-........-.-.................-......................
b_L1PA2_367_689 ....-......................S...............A.V-..-........-.-.................-......................
b_L1PA2_368_690 ....-.........................................-..-........-.-.................-......................
b_L1PA2_369_691 ....-.........................................-..-........-.-.................-......................
b_L1PA2_370_692 ....-........A.........A......................-..-........-.-.................-..........I...........
b_L1PA2_371_693 ....-.........................................-..-........-.-.................-......................
b_L1PA2_372_694 ....-.........................................-..-........-.-.................-......................
b_L1PA2_373_695 ....-...........................*.............-..-........-.-.................-......................
b_L1PA2_374_696 ....-..........D..............................-..-........-.-.................-......................
b_L1PA2_375_697 ....-.........................................-..-........-.-.................-......................
```

```
1.2_o_50%_cn     YSELREDIQTKGKEVENFEKNLEECITRITNTEKCLKELMELKTKARELREECRSLRSRCDQLEERVSAMEDEMNEMKREGKFREKRIKRNEQSLQEIWDY
                 ------^------^------^------^------^----+++--^------^------^------^------^------^------^------^------^
b_L1PA2_376_698  ....-.........................................-..-........-.-.................-.......................
b_L1PA2_377_699  ....-.........................................-..-........-.-.................-..................M..D
b_L1PA2_378_700  ....-......D..D...............................-..-....N...-.-.................-..R....................
b_L1PA2_379_701  ....-.................*....................A..-..-........-.-........T........-.......................
b_L1PA2_380_702  ....-.........................................-..-........-.-................R-.....D.............R..
b_L1PA2_381_703  ....-.........................................-..-.*......-.-.................-.......................
b_L1PA2_382_704  ....-.........................................-..-........-.-.................-.......I...............
b_L1PA2_383_705  ....-...............H.........................-..-........-.-.................-.......................
b_L1PA2_384_706  ....-.........................................-..-........-.-........V........-......................S
b_L1PA2_385_707  ....-.........................................-..-........-.-.................-.......................
b_L1PA2_386_708  ....-..............E..........................-..-........-.-........R........-........L..............
b_L1PA2_387_709  ....-.........................................-..-........-.-.................-.......................
b_L1PA2_388_710  ....-.........................................-..-........-.-....GX...........-.......................
b_L1PA2_389_711  ....-.............Q...........................-..-........-R-.................-.......................
b_L1PA2_390_712  ....-.........................................-..-........-.-.................-.......................
b_L1PA2_391_713  ....-.........................................-..-........-.-........R........-.......G...............
b_L1PA2_392_714  ....-........Q................................-..-........-.-.M...............-.......................
b_L1PA2_393_715  ....-.........................................-..-........-.-.................-.......................
b_L1PA2_395_716  ....-...............I.......................N.-..-........-.-.................-........K..............
b_L1PA2_396_717  ....-.........................................-..-........-.-.................-.......................
b_L1PA2_397_718  ....-...........................D.............-..-........-Y-.................-.......................
b_L1PA2_398_719  .T..-.........................................-.V-........-.-.......V.........-.....G.................
b_L1PA2_399_720  ....-.........................................-..-........-.-.................-.......................
b_L1PA2_400_721  ....-........Q................................-..-........-.-.................-.......................
b_L1PA2_401_722  ....-.........................................-..-........-.-.................-.......................
b_L1PA2_402_723  ....-.................*.......................-..-........-.-.................-..........I............
b_L1PA2_403_724  ....-.........................................-..-........-.-.................-.......................
b_L1PA2_404_725  ....-.........................................-..-........-*-.................-.......................
b_L1PA2_405_726  ....-................................*........-..-........-.-.................-.......................
b_L1PA2_406_727  ....-.....E...................................-..-........-.-.................-.......................
b_L1PA2_407_728  ....-.........................................-..-........-.-.................-.......................
b_L1PA2_408_729  ....-.........................................-..-........-.-.........Q.......-.......................
b_L1PA2_409_730  ....-.........................................-..-........-.-.................-.......................
b_L1PA2_410_731  .C..-.........................................-..-........-.-.................-.............K........
b_L1PA2_411_732  ....-.........................................-..-........-.-.................-.......................
b_L1PA2_412_733  ....-........K................................-..-........-.-.................-.......................
b_L1PA2_413_734  ....-.........................................-..-........-.-............I....-.......................
b_L1PA2_414_735  ....-.........................................-..-........-.-.................-.......................
b_L1PA2_415_736  ....-.........................................-..-........-.-.................-.......................
b_L1PA2_416_737  ....-.........................................-..-........-.-.................-.......................
b_L1PA2_417_738  ....-.............X........................S..-..-........-.-.................-.......................
b_L1PA2_418_739  ....-...............K....K....................-..-........-.-.................-.......................
b_L1PA2_419_740  ....-...............H.........................-..-........-.-.................-.......................
b_L1PA2_420_741  ....-.........................................-..-........-.-.................-.......................
b_L1PA2_421_742  ....-......................................P..-..-........-.-.................-.......................
b_L1PA2_422_743  ....-.........................................-..-........-.-.................-.......................
b_L1PA2_423_744  ....-.........................................-..-........-.-.................-.......................
b_L1PA2_424_745  ....-.........................................-..-........-.-.................-......N................
b_L1PA2_425_746  ....-.........................................-..-........-.-.................-.......................
b_L1PA2_426_747  ....-.........................................-..-........-.-.................-.......................
b_L1PA2_427_748  ....-.........................................-..-........-.-.................-.......................
b_L1PA2_428_749  ....-.........................................-..-........-.-.................-.......................
b_L1PA2_429_750  ....-.........................................-..-........-.-.................-.......................
b_L1PA2_430_751  ....-.........................................-..-........-.-.................-.......................
b_L1PA2_431_752  ....-..........G..............................-..-........-.-.................-.......................
b_L1PA2_432_753  ....-.........................................-..-........-.-...........XX....-.......................
b_L1PA2_433_754  ....-.........................................-..-........-.-.................-.......................
b_L1PA2_434_755  ....-.........................................-..-........-.-.................-.......................
b_L1PA2_435_756  ....-.........................................-..-........-.-.................-.......................
b_L1PA2_436_757  ....-.........................................-..-........-.-.................-.......I...............
b_L1PA2_437_758  ....-.........................................-..-........-.-.................-.......................
b_L1PA2_438_759  ....-.........................................-..-........-.-..........A......-.......................
b_L1PA2_439_760  ....-.........................................-..-........-.-.................-.......I...............
b_L1PA2_440_761  ....-......................................A..-..-........-.-.................-.......................
```

```
1.2_o_50%_cn     YSELREDIQTKGKEVENFEKNLEECITRITNTEKCLKELMELKTKARELREECRSLRSRCDQLEERVSAMEDEMNEMKREGKFREKRIKRNEQSLQEIWDY
                 ------^------^------^------^------^----+++--^------^------^------^------^------^------^------^------^
b_L1PA2_441_762  ....-.........................................-..-........-.-.................-......................
b_L1PA2_442_763  ....-.........................................-..-........-.-.................-......................
b_L1PA2_443_764  ....-.........................................-..-........-.-.................-......................
b_L1PA2_444_765  ....-.........................................-..-........-.-.................-......................
b_L1PA2_445_766  ....-.........................................-..-........-.-.................-......................
b_L1PA2_446_767  ....-.........................................-..-........-.-.................-......................
b_L1PA2_447_768  ....-.........................................-..-........-.-.................-......................
b_L1PA2_448_769  ....-.........................................-..-........-.-.................-......................
b_L1PA2_449_770  ....-.........................................-..-........-.-.................-......................
b_L1PA2_450_771  ....-.........................................-..-........-.-.................-......................
b_L1PA2_451_772  ....-.........................................-..-........-.-.................-......................
b_L1PA2_452_773  ....-..L......................................-..-........-.-.................-......................
b_L1PA2_453_774  ....-.........................................-..-........-.-.................-......................
b_L1PA2_454_775  .P..-..................G......................-..-........-.-.................-........KE............
b_L1PA2_455_776  ....-.........................................-..-........-.-.................-......................
b_L1PA2_456_777  ....-....................................R....-..-.Q......-.-.................-....................V.
b_L1PA2_457_778  ....-........*................................-..-........-.-.................-......................
b_L1PA2_458_779  ....-.........................................-..-........-.-.................-......................
b_L1PA2_459_780  D...-.........................T...............-..-.....F..-.-.................-..................V...
b_L1PA2_460_781  ....-.........................................-..-..Y.....-.-.................-......................
b_L1PA2_461_782  ....-.........................................-..-........-.-.................-......................
b_L1PA2_462_783  ....-......C..................................-..-...G....-.-.................-......................
b_L1PA2_463_784  ....-.........................................-..-........-.-.................-......................
b_L1PA2_464_785  ....-........................P........M.......-..-........-.-.................-......................
b_L1PA2_465_786  ....-.........................................-..-........-.-.................-......................
b_L1PA2_466_787  ....-.........................................-..-........-.-...............V.-......................
b_L1PA2_467_788  ....-....................T....................-..-........-.-.................-......................
b_L1PA2_468_789  ....-...HS.................................I..-..-........-.-.........Q.......-K.....................
b_L1PA2_469_790  ....-..................V......................-..-........-.-.................-......................
b_L1PA2_470_791  ....-.........................................-..-........-.-.................-......................
b_L1PA2_471_792  ....-.........................................-..-........-.-.................-......................
b_L1PA2_472_793  ....-.........................................-..-........-.-.................-......................
b_L1PA2_473_794  ....-............L............................-..-........-.-.................-............A.........
b_L1PA2_474_795  ....-.........................................-..-........-.-.................-......................
b_L1PA2_475_796  ....-.........................................-..-........-.-.................-......................
b_L1PA2_476_797  ....-.........................................-..-....I...-.-.................-.........Q..G.........
b_L1PA2_477_798  ....-.........................................-.V-.K......-.-.................-......................
b_L1PA2_478_799  ....-.........................................-..-........-.-.................-......................
b_L1PA2_479_800  ....-.................................P.......-..-........-.-.......T.........-......................
b_L1PA2_481_801  ....-.........................................-..-........-.-.................-......................
b_L1PA2_482_802  ....-.........................................-..-........-.-.................-......................
b_L1PA2_483_803  ....-..........K..............................-..-........-.-........T........-......................
b_L1PA2_484_804  ....-.........................................-..-........-.-.................-......................
b_L1PA2_485_805  ....-......................T..................-..-........-.-.................-......................
b_L1PA2_486_806  ..*.-.G.......................................-..-........-.-.................-......................
b_L1PA2_487_807  ....-.........I.....K..X.........N............-..-........-.-.................-......................
b_L1PA2_489_808  ....-.........................................-..-........-.-.................-...........K..........
b_L1PA2_490_809  ....-.........................................-..-........-.-.................-......................
b_L1PA2_491_810  ....-.............................*...........-..-........-.-.................-......................
b_L1PA2_492_811  ....-.........................................-..-D.......-.-............T....-.....D................
b_L1PA2_493_812  ....-.........................................-..-........-.-.................-......................
b_L1PA2_494_813  ....-.........................................-..-........-.-R................-......................
b_L1PA2_496_814  ....-......V..................................-..-........-.-.................-......................
b_L1PA2_498_815  ....-.........................................-..-........-.-.................-......................
b_L1PA2_499_816  ....-......V..................................-..-........-.-.................-......................
b_L1PA2_500_817  ....-.........................................-..-........-.-.................-......................
b_L1PA2_501_818  ....-.........................................-..-........-.-..K....T.........-......................
b_L1PA2_502_819  ....-.........................................-..-........-.-.................-......................
b_L1PA2_503_820  ....-.........................................-..-........-.-.................-......................
b_L1PA2_504_821  ....-......................T...R..............-..-........-.-.................-......................
b_L1PA2_506_822  ....-.........................................-..-........-.-.................-......................
b_L1PA2_507_823  ....-.H.......................................-..-........-.-.................-.................*....
b_L1PA2_508_824  ....-.........................................-..-........-.-.................-........V.............
b_L1PA2_509_825  ....-.........................................-..-........-W-.................-......................
```

```
1.2_o_50%_cn    YSELREDIQTKGKEVENFEKNLEECITRITNTEKCLKELMELKTKARELREECRSLRSRCDQLEERVSAMEDEMNEMKREGKFREKRIKRNEQSLQEIWDY
                ------^------^------^------^------^----+++--^------^------^------^------^------^------^------^------^
b_L1PA2_510_826 ....-.........................................-..-........-.-.................-.....X................
b_L1PA2_511_827 ....-............*X...........................-..-........-.-.......V.........-......................
b_L1PA2_512_828 ....-.........................................-..-........-.-.................-......................
b_L1PA2_513_829 ....-.........................................-..-........-.-.................-......................
b_L1PA2_514_830 ....-.........................................-..-........-.-.................-......................
b_L1PA2_515_831 ....-.........................................-..-........-.-.................-.................D....
b_L1PA2_516_832 D...-.........................................-..-........-.-.................-......................
b_L1PA2_517_833 ....-...E.....................................-..-........-.-.................-......................
b_L1PA2_518_834 ....-..................K.............K........-..-........-.-.................-D.....................
b_L1PA2_519_835 ....-...................Y.....................-..-........-.-.....I...........-......................
b_L1PA2_520_836 ....-.........................................-..-........-.-.................-......................
b_L1PA2_521_837 ....-.H.......................................-..-........-.-...............R.-......................
b_L1PA2_522_838 ....-........................I................-..-........-.-.................-......N...............
b_L1PA2_523_839 ....-.............................S...........-..-........-.-L................-......................
b_L1PA2_524_840 ....-...................Y.....................-..-........-.-.................-......................
b_L1PA2_525_841 ....-.........................................-..-........-.-.................-......................
b_L1PA2_526_842 ....-.........................................-..-........-.-.................-......................
b_L1PA2_527_843 ....-.........................................-..-........-.-.................-......................
b_L1PA2_528_844 ....-.........................................-..-........-.-.................-......................
```
