# Supplementary material for: Cryptic genetic variation enhances primate L1 retrotransposon survival by enlarging the functional coiled coil sequence space of ORF1p
Source: PLoS Genet. 2020 Aug 14;16(8):e1008991. doi: 10.1371/journal.pgen.1008991 (PMC7449397; doi:10.1371/journal.pgen.1008991)
Supplement: S10 Fig — Alignment of L1Pa2 CG-null coiled coil peptide sequences that lack F at position 134 vs the 50% consensus sequence of L1Pa2. (PDF) [file pgen.1008991.s010.pdf]

```
                        60        70        80        90       100       110       120       130       140       150
                --- ----|---- ----|---- ----|---- ----|---- ----|---- ----|---- ----|---- ----|---- ----|---- ----|--
        heptad     1  |   2  |   3  |   4  |   5  |  6 stm  |   7  |   8  |   9  |  10  |  11  |  12  |  13  |  14  |
                ------^------^------^------^------^----+++--^------^------^------^------^------^------^------^------^
                abcdefgabcdefgabcdefgabcdefgabcdefgabcdbcdefgabcdefgabcdefgabcdefgabcdefgabcdefgabcdefgabcdefgabcdefg
2ab_50_cns      YSELREDIQTKGKEVENFEKNLEECITRITNTEKCLKELMELKTKARELREECRSLRSRCDQLEERVSAMEDEMNEMKREGKFREKRIKRNEQSLQEIWDY

a_L1PA2_4_1     ...P-KEVR.H....K.L..K.D.WL.....A..S..D......TT-..-D..T..S.-F-.......V.K.Q.....-.K.C....V...KK........
a_L1PA2_37_2    ....-.........................................-..-........-.-.................-...V..................
a_L1PA2_49_3    ....-...........................A.............-..-.....H..-.-.................-...V..................
a_L1PA2_72_4    ....-.................D.......................-..-........-.-.................-...S..................
a_L1PA2_84_5    ....-........K................................-..-........-.-.................-...V..................
a_L1PA2_88_6    ....-...........................G.............-..-........-.-.................-...Y..................
a_L1PA2_115_7   ..A.-..........................P....N.........-..-........-.-.......V.........-...S............R.....
a_L1PA2_122_8   ....-....N....................................-..-........-.-.......V.........-.R.SI.................
a_L1PA2_129_9   ....-...............K....................M....-..-.G..NF..-.-.........A.......-...S..................
a_L1PA2_130_10  ....-...............K...........Q........M....-..-.G..N...-.-.........A.......-...S........K.........
a_L1PA2_133_11  ..Q.-...........................KN............-..-......W.-.-...D.............-...S...............R..
a_L1PA2_138_12  ....-..............Q..........................-D.-........-.-.....I.V.........-...S..................
a_L1PA2_139_13  ....-................S........................-..-........-.-.................-...Y..QT....K.........
a_L1PA2_159_14  ....-......D.............................P....-..-........-.-.................-...S..................
a_L1PA2_173_15  ....-.........................................-..-........-.-.......V.........-...S..................
a_L1PA2_175_16  ....-.G..............*........................-..-........-.-.....X...........-...S..E...............
a_L1PA2_186_17  ....-...............G.........................-..-........-.-.................-...L.............A....
a_L1PA2_187_18  ....-...............G.........................-..-........-.-.................-...L.............A....
a_L1PA2_200_19  ....-.........................................-..-........-.-.............D..-...S..................
a_L1PA2_215_20  ....-.........................................-..-........-.-.................-...S..................
a_L1PA2_218_21  ....-.........................................-..-..S.....-.-.M...............-...L..................
a_L1PA2_245_22  ....-.........................................-..-........-R-.................-...S..................
a_L1PA2_249_23  ....-.........................................-..-........-.-....S....D.......-...S..................
a_L1PA2_265_24  ....-.........................................-..-........-.-.................-...S........*.........
a_L1PA2_274_25  ....-.........................................-..-........-.-.................-...S..................
a_L1PA2_292_26  ....-.........................................-..-........-.-.................-...S..................
a_L1PA2_301_27  ....-.........................................-..-........-.-.................-...S..................
a_L1PA2_302_28  .T..-.......................G.................-..-........-.-.................-...L....X.......P.....
a_L1PA2_310_29  ....-...E.....................................-..-........-.-........T........-...V..................
a_L1PA2_315_30  ....-................V................M.......-..-........-.-.................-...S..................
a_L1PA2_316_31  ....-............X............................-..-........-.-.................-...S..............L...
a_L1PA2_318_32  ....-...........................K.............-..-........-.-.................-...V..................
a_L1PA2_323_33  ....-.........................................-..-........-.-.................-...S..................
a_L1PA2_326_34  ....-................................A........-..-........-.-.................-...S..................
a_L1PA2_328_35  ....-..............................I..........-..-........-.-.................-...L..................
a_L1PA2_333_36  ....-.........A.......................W....A..-..-........-.-.............K...-...S...............L..
a_L1PA2_337_37  ....-..........................I..............-..-........-.-.......T.........-...S..................
a_L1PA2_338_38  ....-..........................K..............-..-........-.-.......V.........-...S..................
a_L1PA2_351_39  ....-......................G..................-..-........-.-.................-...S..................
a_L1PA2_354_40  ...I-.........................................-..-....I...-*-.................-...S...............R..
a_L1PA2_355_41  ....-.........................................-..-........-.-.................-X-.S..................
a_L1PA2_361_42  ....-.......N..................R..............-..-........-.-.................-...S..................
a_L1PA2_363_43  ....-.........................................-..-........-.-.......V.........-...S..................
a_L1PA2_374_44  ....-.........................................-.*-.Q......-.-.................-...S..................
a_L1PA2_375_45  ....-.................................V.......-..-........-.-.................-...S..................
a_L1PA2_381_46  ....-................................*........-..-........-.-.................-...S..................
a_L1PA2_384_47  ....-K....E...................................-..-........-.-.................-...S...............R..
a_L1PA2_389_48  ....-...............K.........................-..-........-.-.................-...S.K................
a_L1PA2_390_49  ....-.........................................-..-........-.-.................-...S..................
b_L1PA2_3_50    ....-...............D.........................-..-........-.-.................-.A.V.........*........
b_L1PA2_7_51    ....-....................V.............I......-..-........-.-.............D..-...V..................
b_L1PA2_10_52   ....-.................D.......................-..-........-.-.................-...V.A.T..........K...
b_L1PA2_15_53   ....-.........................................-..-........-.-.................-...V..................
b_L1PA2_16_54   ..D.-.........................................-..-........-.-.................-...V..................
b_L1PA2_18_55   ....-.........................................-..-........-.-.................-...V..................
b_L1PA2_20_56   ....-.............................S...........-..-........-.-.................-...V..................
b_L1PA2_22_57   ....-.........................................-..-........-.-..............I.-...V..................
```

```
  2ab_50_cns    YSELREDIQTKGKEVENFEKNLEECITRITNTEKCLKELMELKTKARELREECRSLRSRCDQLEERVSAMEDEMNEMKREGKFREKRIKRNEQSLQEIWDY
                ------^------^------^------^------^----+++-_^------^------^------^------^------^------^------^------^
b_L1PA2_25_58   ....-.........................................A..-..-..F.....-.-.........K.....-...V...................
b_L1PA2_26_59   ....-.........................................-..-........-.-.................-...V...................
b_L1PA2_27_60   ....-.........................................-..-........-.-.................-...V...................
b_L1PA2_28_61   ....-....................S...............-..-........-.-.................-...V........K.........
b_L1PA2_30_62   ....-.........................................-..-........-.-.................-...V.............D....
b_L1PA2_31_63   ....-.........................................-..-........-.-........V........-...V...................
b_L1PA2_36_64   ....-.........................................-..-........-.-.................-...V.X.................
b_L1PA2_37_65   ....-....................................X....-..-......K.-.-.................-...V...................
b_L1PA2_39_66   ....-.........................................-..-........-.-.................-...V................G.
b_L1PA2_40_67   ....-.........................................-..-........-.-................N-...V...................
b_L1PA2_41_68   ....-.........................................-..-........-.-.................-...V...................
b_L1PA2_42_69   ....-........................................V-..-........-.-.................-...V...................
b_L1PA2_43_70   ....-.........................................-..-........-.-.................-...V.......H..........
b_L1PA2_65_71   ....-.........................................-..-........-.-.................-...L...................
b_L1PA2_83_72   ....-.........................................-..-........-.-.................-...S...................
b_L1PA2_84_73   ....-.........................................-..-........-.-.................-.E.SX..................
b_L1PA2_123_74  ....-...............S.........................-..-........-.-.................-...V...................
b_L1PA2_124_75  ....-.........................................-..-........-.-.................-...V....V..............
b_L1PA2_125_76  ....-.........................................-..-........-.-.........*.......-...V............*.....
b_L1PA2_132_77  ....-.........................................-..-........-.-.................-...V...................
b_L1PA2_133_78  ....-.........................................-..-........-.-.................-...V...................
b_L1PA2_134_79  ....-.....................P...................-..-.Q......-.-.................-...V...................
b_L1PA2_136_80  ....-................................Q........-..-........-.-.........*X......-...V...................
b_L1PA2_137_81  ....-.........................................-..-........-.-..............A..-...V...................
b_L1PA2_138_82  ....-.........................................-..-........-.-.................-...V...................
b_L1PA2_139_83  ....-.........................................-..-........-.-.................-...V...................
b_L1PA2_140_84  ....-.........................................-..-........-.-.................-...V.....N............
b_L1PA2_156_85  ....-.................................M.......-..-........-.-......LV.........-...S...................
b_L1PA2_171_86  ....-.........A.......................M.......-..-........-R-.................-.V.S...................
b_L1PA2_177_87  ....-..........K..............................-..-........-S-.................-...S...................
b_L1PA2_222_88  ....-.........................................-..-....R...-.-.................-.E.S..........R.......
b_L1PA2_225_89  ....-................................Q........-..-........-.-.................-...S...................
b_L1PA2_251_90  ....-.........................................-..-........-.-.................-...V...................
b_L1PA2_255_91  ....-.........................................-..-........-.-.................-...L...S...............
b_L1PA2_304_92  ....-....................M...............V....-..-........-.-.................-...V..........N.......
b_L1PA2_342_93  ....-.........................................-..-........-.-.................-...V...................
b_L1PA2_394_94  ....-............XG..........S................-..-........-R-.................-...V...................
b_L1PA2_480_95  ....-.........................................-..-........-.-.......V.........-...V...................
b_L1PA2_488_96  ....-.........................................-..-........-.-.................-...V...................
b_L1PA2_495_97  ....-.........................................-..-.....R..-.-.................-...V...................
b_L1PA2_497_98  ....-..S..................................A..-..-........-.-.................-...V...................
b_L1PA2_505_99  ....-.........................................-..-........-.-.................-...V.....Q......*.....
```
