# Supplementary material for: Cryptic genetic variation enhances primate L1 retrotransposon survival by enlarging the functional coiled coil sequence space of ORF1p
Source: PLoS Genet. 2020 Aug 14;16(8):e1008991. doi: 10.1371/journal.pgen.1008991 (PMC7449397; doi:10.1371/journal.pgen.1008991)
Supplement: S11 Fig — Alignment of the L1Pa3 cluster1 coiled coil peptide sequences vs the 50% consensus sequence of the CG-null cL1.3 coiled coil and the 50% consensus sequences of the L1Pa1 and L1pa2 coiled coils. (PDF) [file pgen.1008991.s011.pdf]

```
                        60        70        80        90       100       110       120       130       140       150
                    ---:----|----:----|----:----|----:----|----:----|----:----|----:----|----:----|----:----|----:----|--
              heptad   1  |   2  |   3  |   4  |   5  |  6 stm  |   7  |   8  |   9  |  10  |  11  |  12  |  13  |  14  |
                    ______^______^______^______^______^____+++__^______^______^______^______^______^______^______^______^
                    abcdefgabcdefgabcdefgabcdefgabcdefgabcdbcdefgabcdefgabcdefgabcdefgabcdefgabcdefgabcdefgabcdefgabcdefg
1.3_o_50%_cn        YS-L-EDIQTKGKEVENFEKNLEECITRITNTEKCLKELMELKTKA-EL-EECRSLRS-CDQLEERVS-MEDEMNEMK-EGKFREKRIKRN-QSLQEIWDY

cL1.1_50%_cn_1:     ..E.................................................................A......................E.........
cL1.2_50%_cn_2:     ..E.................................................................A......................E.........
3_chr3_691-cL1_3:   ......N...........................................................................S..................
3_chr9_1178-cL1_4:  .................................................................K.....................X.X...........
3_chr12_222-cL1_5:  ..................................................................I..V...............................
3_chrX_1343-cL1_6:  ...........................T................................N........................................
3_chr5_892-cL1_7:   ............................V.............................................S..........................
3_chr8_1188-cL1_8:  .......V....................................................N........................................
3_chr9_1167-cL1_9:  .....................F...............................................................................
3_chr13_382-cL1_10: .......................................L.............................................................
3_chr3_581-cL1_11:  .................L..........................................N......L.....V........................G..
3_chr10_170-cL1_12: .....KE.................Y...........ND...P..............................................I............
3_chr11_192-cL1_13: ..................X..........................................K.......................................
3_chr5_851-cL1_14:  .....................................................................I........................F......
3_chrX_1268-cL1_15: .........N..........................................R....................V.............X.X...........
3_chr3_688-cL1_16:  ................................................................................A....................
3_chrX_1289-cL1_17: ..................Q.....Y...........................................................Q............M...
3_chr10_178-cL1_18: .T...................................................................................R...............
3_chr16_469-cL1_19: ......E.............S...Y................................................V..........X--X..S..........
3_chrX_1275-cL1_20: ..............A............................A................N........................................
3_chr5_821-cL1_21:  ...Q..E...........X.K........................................E...A.....H........V....................
3_chrX_1269-cL1_22: ......E..X-.........S......................A.......................T.....V...........................
3_chr5_871-cL1_23:  .Y...................................................................................................
3_chr5_880-cL1_24:  .....................................................................................................
3_chr8_1044-cL1_25: ....................K......................A.................................................T.......
3_chr14_386-cL1_26: .............*......K........A.I.....................................................................
3_chr2_595-cL1_27:  ........................Y............................................................................
3_chr5_841-cL1_28:  .A.V.................*......................................N....K...........................T.......
3_chr3_667-cL1_29:  ..................................................................................I..................
3_chr14_392-cL1_30: ..................*.T................................................................................
3_chr1_73-cL1_31:   ....................S.............................................................V..................
3_chr7_1057-cL1_32: ......................D...S..........................................................................
3_chrY_1303-cL1_33: H........I...*....X.K...................K...........F.G..N.Y..W..............M............H..........
3_chr22_549-cL1_34: ......E..I........Q.S...R..............L....................N............V...................N.......
3_chr16_405-cL1_35: ........................Y.......Q.................................X..................................
3_chr4_776-cL1_36:  .....................................................................................................
3_chr2_494-cL1_37:  ..................X.....Y............................................................................
3_chr19_402-cL1_38: .....G...........................R......Q...................H...QK.....E.............................
3_chr9_1186-cL1_39: ...............................I............................N..........N.................S...........
3_chr2_479-cL1_40:  .....K...................K..................................N........................................
3_chr2_465-cL1_41:  ......E...........Q.....S..*.........D..................XX........A..................T...............
3_chrX_1358-cL1_42: .....................................................................................................
3_chr2_539-cL1_43:  ......................A..................VES..................P..................N...................
3_chr1_45-cL1_44:   ..............A..X...........ID.......V.....................N........T...............................
3_chrX_1323-cL1_45: .........N.......S..S.........K..E....................C..........T.........*......................S..
3_chr6_980-cL1_46:  .....................................................................I...............................
3_chr3_693-cL1_47:  .......T................G.......................Q....................................................
3_chr16_445-cL1_48: ......E...........X..................M.......P..............N........................................
3_chr7_1091-cL1_49: .....................*...............................................................................
3_chr6_846-cL1_50:  ....................S.....I.....................................D........V...........................
3_chr7_1079-cL1_51: ............................................................................R........................
3_chr4_766-cL1_52:  ............................V...............................................T......G........*........
3_chr6_982-cL1_53:  .....................................................................................................
3_chr6_909-cL1_54:  ..........E..........................D..K............................................................
3_chr10_183-cL1_55: ......................................................R...............Q..............................
3_chr9_1191-cL1_56: .........I.......................................................S..................X.............LH.
3_chr5_874-cL1_57:  .............................I..............................N....K...................................
```

```
    1.3_o_50%_cn    YS-L-EDIQTKGKEVENFEKNLEECITRITNTEKCLKELMELKTKA-EL-EECRSLRS-CDQLEERVS-MEDEMNEMK-EGKFREKRIKRN-QSLQEIWDY
                    ------^------^------^------^------^----+++--^------^------^------^------^------^------^------^------^
3_chr7_1046-cL1_58: ..........Q..........................................................................................
3_chr1_53-cL1_59:   ..............................I.............................N........................................
3_chr1_49-cL1_60:   ....................H.....I..................................H....................I..................
3_chr8_1065-cL1_61: .........I.......I..H...................................K...N........................................
3_chr18_476-cL1_62: ......................................................R...............Q..............................
3_chr1_81-cL1_63:   ........................................G...................N........................N.M.....R....C..
3_chrY_1320-cL1_64: ...................E.F..................................................X--X....A....................
3_chr9_1187-cL1_65: ..............L......................K.............................................................N.
3_chr6_955-cL1_66:  .........................V.................N..........................K..............................
3_chrX_1282-cL1_67: ....................................................F.......N..K.....................................
3_chr6_942-cL1_68:  ........................FV...........D..........R..........GK........................................
3_chr10_161-cL1_69: ...........................TR.......................................................Q................
3_chrX_1291-cL1_70: ................S....................................................................Q...............
3_chr2_546-cL1_71:  ......G........................A..H..D...................G..........................D................
3_chr8_1154-cL1_72: ...........................................S........G....................T...........................
3_chrY_1322-cL1_73: .................X................Y.....K...E.................M..........T...........................
3_chr6_959-cL1_74:  C....K............................S..................................................................
3_chr7_1076-cL1_75: ..................A......................................................X--X........................
3_chr20_560-cL1_76: ............................................X..........................G....................*........
3_chr1_97-cL1_77:   .............................................................................................R.......
3_chrX_1345-cL1_78: ...........D.....I.............I.................................................................M...
3_chr12_257-cL1_79: ...................................................Q...P....G......................T.................
3_chr14_383-cL1_80: .....................................................Q......N....S.................T.........G.......
3_chrX_1208-cL1_81: ......E.......D.....S.....A...................................W...L..................................
3_chr1_86-cL1_82:   ....................................................Y...........................................D....
3_chr3_564-cL1_83:  ........H.....................................................M......................................
3_chr2_565-cL1_84:  ..............................D......................................................................
3_chr15_355-cL1_85: ....................S....................................R...........................................
3_chr10_172-cL1_86: ...............X.L....D.....................................N........................................
3_chr14_356-cL1_87: .........I..........S...........................R.K.........N........................................
3_chr15_376-cL1_88: ...........................T...............A................N........................................
3_chr4_773-cL1_89:  ......E......Q..............K........................................................................
3_chr2_588-cL1_90:  .......................................R...............................E.............................
3_chr8_1056-cL1_91: .......S...................................A...............H.............V...........................
3_chr13_374-cL1_92: .........................................M.................*.............I..V........................
3_chr11_174-cL1_93: .P....G....S.................I.A..........E........................................G.................
3_chr12_292-cL1_94: .................................................................T................C..................
3_chr3_610-cL1_95:  ......E.......AD.......D.L..........T............................................E...................
3_chr3_661-cL1_96:  ..........T...A...A......................................G..................T................T...V...
3_chr6_925-cL1_97:  ......E.......H.....T.............................................L..................................
3_chr6_917-cL1_98:  ......E.............S......G.............................................V...........................
3_chr4_791-cL1_99:  ................K.................*.........................N...........-X...................N.......
3_chr3_626-cL1_100: .Y.......I..........K................................................................................
3_chr6_983-cL1_101: ............................................................---......................................
3_chrX_1270-cL1_102:.............................I..K............................................N........T..............
3_chr18_492-cL1_103:.....................................................................................................
3_chr12_281-cL1_104:.........................L..............................W..S.........................................
3_chr17_485-cL1_105:.....................................................................................................
3_chr8_1036-cL1_106:....................K.......................................N........................................
3_chrX_1347-cL1_107:.....D.....D.........................................................................................
3_chr10_101-cL1_108:.......T.............................................................................................
3_chr11_258-cL1_109:.............Q..................Q..........I.............................I...............-X..........
3_chrY_1348-cL1_110:.....D.....D................V.......X...............Y...........*....................................
3_chr22_589-cL1_111:.............................I...............S.......X...................T...........N...............
3_chr4_743-cL1_112: ...........................................A............M..G.........................................
3_chr9_1166-cL1_113:..................G..F...............................................................................
3_chr2_553-cL1_114: ..........................I............KD............................................................
3_chr3_692-cL1_115: ..................................................................................S..................
3_chr1_22-cL1_116:  .......V............................N......N...........................................M.........V...
3_chr13_385-cL1_117:.....................................................................................................
3_chrX_1280-cL1_118:.......T............H.......L.K..............P.......................................................
3_chr5_855-cL1_119: .......................Q....................................N........................................
3_chr5_889-cL1_120: ............................................................N........................................
3_chr3_567-cL1_121: ...........V......................................................L..V...............................
```

```
    1.3_o_50%_cn    YS-L-EDIQTKGKEVENFEKNLEECITRITNTEKCLKELMELKTKA-EL-EECRSLRS-CDQLEERVS-MEDEMNEMK-EGKFREKRIKRN-QSLQEIWDY
                    ------^------^------^------^------^----+++--^------^------^------^------^------^------^------^------^
3_chrX_1206-cL1_122:....................S.............................K...R.....N............V......V....................
3_chr6_945-cL1_123: ..............I...G...............................G..................................................
3_chr2_559-cL1_124: .....................F............................................................................R..
3_chr7_952-cL1_125: .............A......K.......V..............I..........G..............................................
3_chr10_76-cL1_126: ........K............FR......I.............I.............................I...........................
3_chr8_1081-cL1_127:.............................N..............................N.........*.....I........................
3_chr3_625-cL1_128: ..................X............................*............H........................................
3_chrX_1287-cL1_129:N.................................S..................S.................N.............................
3_chr9_1158-cL1_130:......E..N......K.......R...............................S...............K.....................H......
3_chr20_591-cL1_131:....................H..................K..............................K..............................
3_chr3_686-cL1_132: ................K.................................................................................L..
3_chr11_186-cL1_133:........X........L.......................................................V...........................
3_chr4_765-cL1_134: ......................DK..................E................................G.........................
3_chr10_175-cL1_135:..........................................................................................S..........
3_chr10_180-cL1_136:C....................................................................................................
3_chr7_1074-cL1_137:...........................T...........................................................R.............
3_chr10_90-cL1_138: ...........................................A...............S.........................................
3_chr4_689-cL1_139: .....................................................K.....S...........G.............................
3_chr15_398-cL1_140:......................................V.....................N.....I..K...T...........................
3_chr2_480-cL1_141: ......................................................................KY.............................
3_chr6_948-cL1_142: ...Q......................................................................................K..........
3_chr12_278-cL1_143:.........................................................................X-X.........................
3_chr12_290-cL1_144:.....................................................................................................
3_chr14_347-cL1_145:....................................................R....................................G...........
3_chr8_1179-cL1_146:........................*......................................K.....................................
3_chr6_960-cL1_147: ..................................................................................................R.*
3_chr9_1125-cL1_148:...................EK.............Y.....Q......................K.......................T..........C.H
3_chr11_182-cL1_149:.......TR.............A.....T............................................L...........................
3_chr3_680-cL1_150: ......................D....................................R.........................................
3_chrX_1221-cL1_151:.....................*....................R....Q.......P..........................................*..
3_chr1_71-cL1_152:  ............I....................................................X........S...................H......
3_chrX_1239-cL1_153:.F.......................................................................................X...........
3_chr12_280-cL1_154:.....................................................................................................
3_chr7_1087-cL1_155:.....................................................................................................
3_chr11_122-cL1_156:..............L...................*.........................N........................................
3_chr10_94-cL1_157: ..........................................*..........................................................
3_chr8_1147-cL1_158: .................V................*..................................................................
3_chr16_457-cL1_159:.P.........S..........................................N......*.......................................
3_chr10_96-cL1_160: ..........................S............................................E.......D.........G...........
3_chr8_1152-cL1_161:.....................................................................................................
3_chr2_568-cL1_162: ............E................................................................................R.......
3_chr14_346-cL1_163:......................Q........................Q..................I..............................T..*
3_chr18_488-cL1_164:............................................................N........................................
3_chr19_413-cL1_165:..........................I.....................................................W.................*..
3_chr21_545-cL1_166:.....................................................................................Q...............
3_chr2_552-cL1_167: ....................................................S............................M...................
3_chr2_584-cL1_168: ......N......V...........................Q...........................................................
3_chr5_858-cL1_169: ...Q...........................I..........R.................N.............S...........I........*.....
3_chr5_833-cL1_170: ......N...........G......................................N..N............I........I.......D........E.
3_chr10_166-cL1_171:......E........D....S............................................T.......V...........................
3_chr3_694-cL1_172: .....................................................S......H........................................
3_chr16_456-cL1_173:...........................T..........................................K---D........................E.
3_chr2_572-cL1_174: .........................................................................I...........................
3_chr2_551-cL1_175: ............R..G...........................I...............G.........................................
3_chr13_370-cL1_176:..............F.....................R................................................................
3_chr7_971-cL1_177: ....................SS..Y....A..Q.................................A...*..V...........................
3_chrX_1356-cL1_178:.............................................T.......................................................
3_chr2_575-cL1_179: .....................................................................................................
3_chr6_972-cL1_180: ..................K..................................................................................
3_chr10_167-cL1_181:......E.....I.......S...S..............R.................................L..................E........
3_chr3_695-cL1_182: ..................................................K..S......H........................................
3_chr12_298-cL1_183:.....................................................................................................
3_chr9_1181-cL1_184:............................................................N........................................
3_chr4_782-cL1_185: ......................................P............K.................................................
```

```
    1.3_o_50%_cn    YS-L-EDIQTKGKEVENFEKNLEECITRITNTEKCLKELMELKTKA-EL-EECRSLRS-CDQLEERVS-MEDEMNEMK-EGKFREKRIKRN-QSLQEIWDY
                    ------^------^------^------^----+++--^------^------^------^------^------^------^------^
3_chrX_ran-cL1_186: .........N.......S..S.........K..E....................C..........T.........*......................S..
3_chrX_1235-cL1_187:......................................................N..........................N...................
3_chr13_375-cL1_188:S.......E..V........K.................V..................................T...........................
3_chr11_162-cL1_189:..............L................R.............X...........................L...........................
3_chr6_981-cL1_190: ...............................M.....................................................................
3_chr2_583-cL1_191: ..........XS.........................................................................................
3_chrX_1276-cL1_192:*....................................................................................................
3_chr2_574-cL1_193: ......E.............T....................................................V........................R..
3_chr12_272-cL1_194:..................X.........................................H........................................
3_chrX_1284-cL1_195:......E......................I.......................................................................
3_chr4_788-cL1_196: ...I..............................................................................S......I...........
3_chr2_466-cL1_197: .................C.....................T........................D....................................
3_chr8_1073-cL1_198:....................H.......................................N........................................
3_chr9_1190-cL1_199:..............A..............I..............................N............................I...........
3_chr2_437-cL1_200: .....K.......G........K........................*.....................................................
3_chr15_358-cL1_201:......E...........X....................I...............R.............................................
3_chr4_651-cL1_202: ................................................P.............................................P......
3_chr1_99-cL1_203:  ...........D.............L...........................................................................
3_chr3_676-cL1_204: ............................................................N................N.......................
3_chrX_1344-cL1_205:.....................................Q......................N.................................I......
3_chr2_580-cL1_206: .....GN....................I...I.............P..................Q......---D..........................
3_chr11_169-cL1_207:...................................................QH....................................G...........
3_chrX_1350-cL1_208:.......................................................................A.......D.....................
3_chr6_943-cL1_209: ..............A.................................................................R....................
3_chr12_266-cL1_210:.............Q......K..*.....I.......................................T...............N...T...........
3_chrY_1317-cL1_211:..................K.Y..........................Q......................................G............V.
3_chr3_579-cL1_212: ..................X......K...........D...............................................................
3_chrX_1288-cL1_213:...............................R.................................................N...................
3_chr1_95-cL1_214:  .........................L...........................................................................
3_chr2_548-cL1_215: ...............................R.....................................................................
3_chr8_1145-cL1_216:.........................T..................................Y........................................
3_chrX_1219-cL1_217:......E..............S....................N..........................................................
3_chr11_282-cL1_218:............................................................N..........E........................D....
3_chr7_1062-cL1_219:.C....E.............S...........Q........................................V...........................
3_chrX_1263-cL1_220:H......................................................F.T...........................................
3_chr8_1174-cL1_221:..........................................................................S..............G...........
3_chr7_978-cL1_222: .........................T.*.........................................................................
3_chr4_790-cL1_223: ..................Q........................................YH..X................R.........K..........
3_chr4_753-cL1_224: ..................................................K........................XX.....Y.................X
3_chrX_1237-cL1_225:....................H.....S...I.................V........................I...N......G................
3_chr16_404-cL1_226:...............................................................K.....................................
3_chrX_1338-cL1_227:...........................................A................N......................T..........I......
3_chr6_936-cL1_228: ......................................................G..........M....Q..............................
3_chr5_716-cL1_229: .F.....F................................Q.....................................................Q......
3_chr9_1169-cL1_230:.....................................................................................................
3_chr12_277-cL1_231:....................................................................................D.......H........
3_chr19_489-cL1_232:.......................*.....................................................N.......................
3_chr8_1153-cL1_233:.....................................................................................................
3_chr2_487-cL1_234: .......N..........................................K..................................................
3_chr14_381-cL1_235:............................................................N.....................S...*..............
3_chr11_111-cL1_236:......E........K................V..............D...........................K..........KVT--..........
3_chr14_387-cL1_237:.............................I.................Q.....................................................
3_chr5_810-cL1_238: ......E.E........S.............................................V.M.....Y..................-..........
3_chrY_1330-cL1_239:.................I...........I........M..........................T....................S..............
3_chr3_658-cL1_240: ...................................................................................T.................
3_chrX_1244-cL1_241:......................*.....................M...............N........................................
3_chr2_496-cL1_242: .....................................................................................................
3_chr10_199-cL1_243:...................................................D................................................N
3_chrX_1175-cL1_244:..................K.......................................................K..........................
3_chr12_295-cL1_245:....................S......................A.........................................................
3_chr1_87-cL1_246:  .....K................D...................................................................D..........
3_chr10_197-cL1_247:......E....................................................SN........................................
3_chr8_1180-cL1_248:...............................................................................................E.....
3_chr11_195-cL1_249:.....................................................................................................
```

```
    1.3_o_50%_cn    YS-L-EDIQTKGKEVENFEKNLEECITRITNTEKCLKELMELKTKA-EL-EECRSLRS-CDQLEERVS-MEDEMNEMK-EGKFREKRIKRN-QSLQEIWDY
                    ------^------^------^------^------^----+++--^------^------^------^------^------^------^------^------^
3_chr5_872-cL1_250: .....................................................................................................
3_chr3_659-cL1_251: ......N.....................................................N..............K.........................
3_chr2_585-cL1_252: ......................G....................................*..........*................X....*.F......
3_chr13_330-cL1_253:......E..............V.................................................................M.....N.......
3_chr8_1041-cL1_254:......E.....................V...........................................KV..............N.....F......
3_chr1_67-cL1_255:  ......E........................R.......................................E.......................R..*..
3_chr10_193-cL1_256:.....................................................................................................
3_chr13_368-cL1_257:.....GE.............S.............................K........*.............V...........................
3_chr13_351-cL1_258:..............................S......................G..........G..............K.....................
3_chr14_364-cL1_259:......E.......................X..........P......Q....................................................
3_chr6_975-cL1_260: .........................L...........................................................................
3_chr4_662-cL1_261: ........................................D...................N.P......................................
3_chr1_82-cL1_262:  .............................N..........................................................T............
3_chrX_1228-cL1_263:.P....E...........A...................R...........D.......................................-..........
3_chr5_802-cL1_264: ......ET...............Q..........Y.........................N......T.....V...........................
3_chr3_671-cL1_265: ........................................Q...................N..Q...........Q..................V......
3_chr6_951-cL1_266: .........A.......................................................T..........V........................
3_chr4_624-cL1_267: ......E.............S.......V.........V.................T......Q.........V...........................
3_chrX_1349-cL1_268:..................................................K.........N...........G............................
3_chr12_262-cL1_269:...............*....S...........Q.................................X.........T...R...........*.R......
3_chr3_582-cL1_270: .........................................................................T.....*.....N...............
3_chr18_478-cL1_271:...............................................Q.................XX..................................
3_chr3_660-cL1_272: ......................................P.........*...........E...........G............................
3_chrX_1271-cL1_273:C...............S....................................................................................
3_chr14_350-cL1_274:..................KQ....................K.........K........R.........................................
3_chr6_974-cL1_275: .........................................................................V...........................
3_chr5_799-cL1_276: ......V..................V..........................Y............S.................G...............V.
3_chrX_1332-cL1_277:...............*......D.W...............................K....................................R.......
3_chr7_1058-cL1_278:.........................................................................I............S.R............
3_chr5_840-cL1_279: ..................K.....G................................................V...........................
3_chr8_1035-cL1_280:D.....E........K.....................................................................................
3_chr2_421-cL1_281: F........SR................................................R.......L..............L.......Y.........*
3_chrX_1272-cL1_282:...............................................*V................M...................................
3_chr4_736-cL1_283: .........................................Q......................................E....................
3_chr5_839-cL1_284: .P....E............................................................................T..............R..
3_chr7_1051-cL1_285:......E................................I.............................................................
3_chrY_1319-cL1_286:......E.............S..............................K........N.......................V................
3_chr15_389-cL1_287:...........................................................R.........................................
3_chr12_269-cL1_288:.....................................D.......................K.......................................
3_chr2_571-cL1_289: ...................R.......................................W..............K..................I....*..
3_chrX_1346-cL1_290:........E......................................................K............T........................
3_chr8_1182-cL1_291:...................N......................Q...........................................V..............
3_chr8_1050-cL1_292:...........................................................R...................Q.....................
3_chr1_58-cL1_293:  ...........................................A.........................................Q...............
3_chr7_1090-cL1_294:.............D..................D.................D..T...............................................
3_chr8_1066-cL1_295:.....................................................................................................
3_chr4_684-cL1_296: ........................Y..................A......................A..................................
3_chr12_251-cL1_297:....................H.............F..................................................................
3_chr6_969-cL1_298: .................................................................KI..................................
3_chr5_883-cL1_299: ...........................................I...............*.....K..............E..................ES
3_chr12_275-cL1_300:......E.............S....................................................V.......R.....T.............
3_chr14_331-cL1_301:.........N...Q....G....................L.......................I...............D.....................
3_chr8_1055-cL1_302:......E.............S....V..................................N............A...........................
3_chr1_68-cL1_303:  ..................................................K.................................X.K.............D
3_chr4_709-cL1_304: ......E........K.......................T...................G...K......Q.Q............................
3_chr7_1064-cL1_305:......EV.......GD....................................................................................
3_chr8_1173-cL1_306:...........................................................H.........................................
3_chr15_373-cL1_307:...........................................................R.........................................
3_chr18_495-cL1_308:............................................................N..........EA.........L..................
3_chr4_777-cL1_309: .....................................................................................................
3_chr11_287-cL1_310:......................G......I........................R...................Y..........................
3_chrX_1342-cL1_311:.....................................................................................................
3_chr7_1083-cL1_312:...................................................K............................EN...................
3_chr3_678-cL1_313: ..........E.................................................N........................................
```

```
        1.3_o_50%_cn    YS-L-EDIQTKGKEVENFEKNLEECITRITNTEKCLKELMELKTKA-EL-EECRSLRS-CDQLEERVS-MEDEMNEMK-EGKFREKRIKRN-QSLQEIWDY
                        ------^------^------^------^------^----+++--^------^------^------^------^------^------^------^------^
3_chr10_50-cL1_314: C.....................D*.............................................................................
3_chr3_669-cL1_315: ...............................R............................N................................T.......
3_chr11_150-cL1_316:.............K.............T.............................N......................E.....S..............
3_chrX_1354-cL1_317:................................................I....................................................
3_chr1_84-cL1_318:  ......................D.............................F....................V...........................
3_chr4_679-cL1_319: D.......E...................................................N........................................
3_chr10_142-cL1_320:..................X..*....................E..P...................S...................................
3_chr5_745-cL1_321: ......E.............S........I..........................................................R............
3_chr8_1067-cL1_322:........................Y...................................................V........................
3_chr12_268-cL1_323:.........................V......D.......KPNA................N...K.......*............................
3_chr4_754-cL1_324: ...................................................................A.......V....E...*................
3_chr4_727-cL1_325: ......E.............S..........R......................R.X................V....................V.*....
3_chr4_785-cL1_326: ...........................................................SN............I...........................
3_chrY_1335-cL1_327:.................I...........I.......................................................................
3_chrX_1286-cL1_328:................................................................................R....................
3_chr2_540-cL1_329: ......E.....R.......S........................V...........................V........L...............*..
3_chr1_88-cL1_330:  .....................................................................................................
3_chrX_1274-cL1_331:.........................T.....R............................................................R........
3_chr20_563-cL1_332:....................S...............R........................*.......................................
3_chr6_900-cL1_333: ......E..........IX.....L..T......D........A.........*...........SI.....X--X.............X........G..
3_chr5_762-cL1_334: ......E.................................D............................................................
3_chr4_792-cL1_335: ............................................N........*..G............I..............X--X.............
3_chrX_1278-cL1_336:..............................T.............N........................................................
3_chrX_1279-cL1_337:................D..................*...............Q................................................S
3_chr2_578-cL1_338: .................S..S.............................................L......V...........................
3_chr2_593-cL1_339: ..............G................................K............................K........N..........Q....
3_chr7_1086-cL1_340:.....Q.V....................................................................I........................
3_chr1_80-cL1_341:  ..............I........................................................................M.............
3_chrX_1250-cL1_342:...P...............N......I....................................Q.T...................................
3_chr6_977-cL1_343: .....................................................................................................
3_chr11_139-cL1_344:.............D..................GM.........S.....................T.........G...........K.............
3_chr9_1170-cL1_345:.....................................................................................................
3_chr12_235-cL1_346:.................L...........X..................................Q...........I.....................*..
3_chr12_291-cL1_347:.........I......................D....................................................................
3_chr1_77-cL1_348:  .....................V...............................................................................
3_chr7_1089-cL1_349:...................................*.........T..............N.....................I..................
3_chr5_779-cL1_350: .Y..........................................................H.Q......................................
3_chr1_75-cL1_351:  .Y....E.............S...............................G....................V.........................N.
3_chr16_477-cL1_352:....................................................R.......N........................................
3_chrX_1218-cL1_353:.......F...C..........................................N........Q...........K...........M......I...*..
3_chr21_500-cL1_354:.....K...........I.....G.....I.............A.V..V..........Y.....T.......VD.....R...........*........
3_chr2_566-cL1_355: ....................................................S..........Q...T.I...............................
3_chr4_750-cL1_356: ........................Y...K....................................M..............E......T.............
3_chr5_893-cL1_357: .........................................................................................T...........
3_chr5_798-cL1_358: *....K.........L.IF*K..........MK..................K........N*............K............K.............
3_chr5_873-cL1_359: ..................K..................................................................................
3_chr2_474-cL1_360: ...................T.................................................................................
3_chr10_184-cL1_361:................................................................................R.V..................
3_chr11_293-cL1_362:......V..............................................................................................
3_chr19_473-cL1_363:.........................T..........................*................................................
3_chrX_1333-cL1_364:......E.............S...................*................................V...........................
3_chr10_177-cL1_365:............................V...............................N..................X-...........H........
3_chr10_188-cL1_366:............................X...................I................G...................................
3_chr2_482-cL1_367: ...............D........Y............................*........................................R......
3_chr12_265-cL1_368:.............K......................*..L...............P................K........T...................
3_chr6_950-cL1_369: ........K.........................*....L.....................E.........................K.G...........
3_chr6_938-cL1_370: .............A..............................................N........K...............................
3_chr4_769-cL1_371: ......E..................................................................V......X....................
3_chrY_1311-cL1_372:..........E..............................................................A.........G.................
3_chrX_1264-cL1_373:.......M...................................A...........F.............................................
3_chr20_577-cL1_374:....................................................................................D............T...
3_chrX_1133-cL1_375:.....................................K.T...A...................D..L..........E..X....................
3_chr3_687-cL1_376: ......E.....................................................N........................................
3_chr12_300-cL1_377:.........................M...................V.......................................................
```

```
    1.3_o_50%_cn    YS-L-EDIQTKGKEVENFEKNLEECITRITNTEKCLKELMELKTKA-EL-EECRSLRS-CDQLEERVS-MEDEMNEMK-EGKFREKRIKRN-QSLQEIWDY
                    ------^------^------^------^------^----+++--^------^------^------^------^------^------^------^------^
3_chr11_148-cL1_378:............................................................N..V..I.........................*..E.....
3_chr8_1176-cL1_379:................................D..................................................................N.
3_chr1_74-cL1_380:  .........................VA...................................V..........I...........................
3_chr21_550-cL1_381:...............Q............................................N......................................N.
3_chr12_286-cL1_382:...................E...........................D....R...........D....................................
3_chr12_285-cL1_383:.................L.E...........................D.....................................................
3_chr4_774-cL1_384: .....X...................L............................R..........................T...................
3_chr5_859-cL1_385: ...............................R........................G............................Q............R..
3_chr1_56-cL1_386:  ...............................................K.....................L......................H....X...
3_chr6_991-cL1_387: ......................................................N..............VD..............................
3_chrX_1222-cL1_388:............N..................A..........................................................K..T.......
3_chr5_861-cL1_389: .............................N...............................................................G.......
3_chr2_573-cL1_390: ....................T........I....................................I......V..........K*...............
3_chr16_491-cL1_391:..................................................................................S...T..............
3_chr5_868-cL1_392: ...I...............................................D........N.............S..........................
3_chr18_454-cL1_393:..................................Y..........................*.......................................
3_chr5_884-cL1_394: ......E...........G............................K.....................................................
3_chr8_1060-cL1_395:...............X--X.....................................................................Q............
3_chr1_83-cL1_396:  ............................................................N...........N..........................N.
3_chr8_1034-cL1_397:......................K..............................K...............................................
3_chr7_979-cL1_398: ....................I...................D............................................................
3_chr7_1059-cL1_399:......NT.....................................................K........................G.............H
3_chr9_1183-cL1_400:............................V............R...X.....................X.................................
3_chr12_228-cL1_401:...........................G........E.................R.............................X................
3_chrX_1357-cL1_402:............................K........................................................................
3_chr10_165-cL1_403:.T.................................*.............................T...............................T...
3_chr9_1172-cL1_404:.....................................................................................................
3_chr1_66-cL1_405:  .Y.........................................................SN.....................L..................
3_chr9_1177-cL1_406:..................................................................................Y..................
3_chr7_992-cL1_407: .....V...................................................................I...........................
3_chr13_396-cL1_408:.....................................K...............................................................
3_chr3_672-cL1_409: ....................................................................................................C
3_chr14_394-cL1_410:........................................................................................M............
3_chr2_484-cL1_411: ....................................Q.......................N........................................
3_chr7_1092-cL1_412:.........................RI..........................................................................
3_chr10_191-cL1_413:.............................................................................T.......................
3_chr11_244-cL1_414:....................H.......K..............A...................Q...................................H.
3_chr10_63-cL1_415: ...I.............S..........................................E...................R....................
3_chr4_768-cL1_416: ..............I...............K...S.T................................................................
3_chr11_113-cL1_417:.......................*........Q......V.......................................................R.....
3_chr3_650-cL1_418: ........................G...............G................N...........................................
3_chr2_569-cL1_419: .................S...S....S.................................N.....E......V...........................
3_chr6_990-cL1_420: .........................V...I.........I.....................E........................T...........*..
3_chrX_1230-cL1_421:......E.K................V.................X.........................................................
3_chr8_1171-cL1_422:........................................................................N............................
3_chr6_985-cL1_423: ...........................................S...D........................N.K....D............K........
3_chr2_486-cL1_424: ....................................................................................................H
3_chr1_69-cL1_426:  ......E..................................................R.........A.....V.........................G.
3_chr11_194-cL1_427:..........................................................................................H..........
3_chr19_416-cL1_428:......E...........D......T...........................G.......*..V........V...........................
3_chr12_294-cL1_429:............................................................S...............I....................M...
3_chr3_654-cL1_430: ......E..........L..........................T........................................................
3_chr5_742-cL1_431: .............................I......................G..................---D..................N.......
3_chr13_384-cL1_432:...P...................K.R.....I..S..................K...............................................
3_chrY_1312-cL1_433:.....K...............S...T..................................................K...........E............
3_chr15_321-cL1_434:.........................................................................V...........................
3_chr9_1161-cL1_435:.......................*.....................V.......................................................
3_chrX_1292-cL1_436:.....................................................................................................
3_chr12_261-cL1_437:................S.......Y............................................................................
3_chr11_198-cL1_438:...................E...................T.............................................................
3_chr6_967-cL1_439: ........................*.............................................................G..............
3_chr11_189-cL1_440:.....................................................................................................
3_chr5_786-cL1_441: .............................................................K....................Y..........N.......
3_chr5_852-cL1_442: .P..................S.................P.....................N......................................E.
```

```
      1.3_o_50%_cn    YS-L-EDIQTKGKEVENFEKNLEECITRITNTEKCLKELMELKTKA-EL-EECRSLRS-CDQLEERVS-MEDEMNEMK-EGKFREKRIKRN-QSLQEIWDY
                    ------^------^------^------^------^----+++--^------^------^------^------^------^------^------^------^
3_chr6_947-cL1_443: .........................................M..................N..*....................................C
3_chr5_879-cL1_444: ........................................*...................N....G......Q............................
3_chr5_770-cL1_445: ................SY.......................................................V..........X................
3_chr6_953-cL1_446: ........................R..............I...I.................................................R.......
3_chr8_1143-cL1_447:........R......Q..................................................I......V.........*.................
3_chrX_1341-cL1_448:.............................I..............................N....S...................................
3_chr8_1137-cL1_449:......................X.Y......RD.................K...G.....N........................................
3_chr17_490-cL1_450:.......M................S..............I...................R.................................N.......
3_chr7_988-cL1_451: .....................................................................................................
3_chr5_837-cL1_452: ........................R......R...........................................A.....T...................
3_chr9_1164-cL1_453:.....................................................................................................
3_chr15_468-cL1_454:..........N.......................................................................................C..
3_chr11_157-cL1_455:...........................................S.........................T.H.............................
3_chr4_732-cL1_456: ...........R.........................................................................................
3_chr1_100-cL1_457: .............G.......................................................................................
3_chr2_544-cL1_458: ......ET.......K....SV...K........F.........................N............V...........................
3_chr10_130-cL1_459:*.....E.............S......................A.......................L.....V...........................
3_chr1_47-cL1_460:  ......E.....................................................N......................................N.
3_chr5_869-cL1_461: ....................S.............................K.R....C..N.....L......V...........................
3_chr14_362-cL1_462:...P.......D................................................N..........E..........L...S...K..XI......
3_chr7_1085-cL1_463:......................................................R...........................................LR.
3_chr9_1168-cL1_464:.....................................................................................................
3_chr1_85-cL1_465:  ...................................................................L...G.....................N....R..
3_chr15_366-cL1_466:.....................................................................................................
3_chr8_1045-cL1_467:......E..........Y..S............N.......P........K.........Y..V.........V...........................
3_chr7_1048-cL1_468:....................D................................................................................
3_chr10_156-cL1_469:....................D.................R.......................................................F*...H.
3_chr13_380-cL1_470:....................................................Y...S............................................
3_chr6_986-cL1_471: C..........................................S................................................K........
3_chr14_363-cL1_472:......................DG...............V.....................................N.......................
3_chr8_1069-cL1_473:.....................................................................................................
3_chr4_772-cL1_474: .......................G.......A...........A................N........................................
3_chrX_1331-cL1_475:...............*......D.W...............................K....................................R.......
3_chr9_1163-cL1_476:....................D................................................................E...............
3_chr15_418-cL1_477:...........D...........................I....................H..........................XX............
3_chr15_372-cL1_478:............................V...D....................................................................
3_chr5_876-cL1_479: .......T......L.......D....................................R.........................................
3_chr10_72-cL1_480: ................................................R..K........................................R.....*.*
3_chr12_273-cL1_481:.................C........................I..............................V..T.........*..............
3_chr14_361-cL1_482:......E..........LX.S...Y.....I...F........A.............................V...........................
3_chr2_483-cL1_483: ....................K......................P...................................K.....................
3_chrX_1205-cL1_484:.............*...L...S.........IG.........N........................L........I........................
3_chr7_1070-cL1_485:.............................I.......................................I................T...K........N.
3_chr11_187-cL1_486:......E..........C..S......................................R.............V...................T.......
3_chr15_369-cL1_487:.....................................................................................................
3_chr17_472-cL1_488:............................L..........................................---D..........................
3_chr13_379-cL1_489:.....................................................................................................
3_chr6_970-cL1_490: ...............................................D.........................K........................*..
3_chrX_1258-cL1_491:......E............N...........I..............................M......T...........................M*..
3_chr11_196-cL1_492:.................................................................S...................................
3_chr6_934-cL1_493: ......E.............S....................................................V...........................
3_chrX_1251-cL1_494:................................................................................R....................
3_chr4_763-cL1_495: ..................................................K...G.....H..X.....................................
3_chr6_987-cL1_496: ....................T......................................S.........................................
3_chr11_176-cL1_497:.......V.............................D.....A.........................................I...............
3_chr15_365-cL1_498:.....K..KI...Q....................*........................................D....R......V.............
3_chr3_649-cL1_499: ............................L......................................T......................K...I......
3_chr2_464-cL1_500: ......................................................R.........D.................C..................
3_chr9_1122-cL1_501:.............*......S........................T................V..........V........V..................
3_chr7_1043-cL1_502:............R....X....................M..............................................................
3_chr1_70-cL1_503:  .P....ET............S..*..........W....................P.................V...................T.......
3_chr11_179-cL1_504:........R....................I.......................................................................
3_chr14_395-cL1_505:.........................................................................X--X........................
3_chr9_1146-cL1_506:....................S.........................................W..........V...........................
```

```
    1.3_o_50%_cn    YS-L-EDIQTKGKEVENFEKNLEECITRITNTEKCLKELMELKTKA-EL-EECRSLRS-CDQLEERVS-MEDEMNEMK-EGKFREKRIKRN-QSLQEIWDY
                    ------^------^------^------^------^----+++--^------^------^------^------^------^------^------^------^
3_chr11_145-cL1_507:......E......D.....E...................................FSR.F.........................................
3_chr3_666-cL1_508: ..............I............K.................................P.......................................
3_chr14_360-cL1_509:..................................................................*.......................K.......R..
3_chr9_1192-cL1_510:............................................................Y........................................
3_chr6_984-cL1_511: ......................................................N................................T.............
3_chr2_590-cL1_512: C.........................N..........................................................................
3_chr3_555-cL1_513: ..................................................K.*..................E.............................
3_chr6_968-cL1_514: ........................*......R......................................................G..............
3_chrX_1277-cL1_515:.........N...........................................................................................
3_chr9_1115-cL1_516:......E...............N..............................................................................
3_chr2_561-cL1_517: .......M.........C......F...................................N.....................V..................
3_chr13_371-cL1_518:.....................................................GR.........................E....................
3_chr14_391-cL1_519:..............................K.G...........................N............R...........................
3_chr16_481-cL1_520:...........................................................Y.........................R.......I.......
3_chr5_805-cL1_521: ........................R......R...........A.......................*..................G.............H
3_chr3_675-cL1_522: .......................................V...............................................M.............
3_chr11_249-cL1_523:....................................................................................Q................
cLs1.3_50%cns_524:  .....................................................................................................
```
