# Supplementary material for: Cryptic genetic variation enhances primate L1 retrotransposon survival by enlarging the functional coiled coil sequence space of ORF1p
Source: PLoS Genet. 2020 Aug 14;16(8):e1008991. doi: 10.1371/journal.pgen.1008991 (PMC7449397; doi:10.1371/journal.pgen.1008991)
Supplement: Attachment — Submitted filename: Furano_response_to_reviewers_062520.docx [file pgen.1008991.s013.docx]

We appreciate and value the efforts expended by the reviewers in reviewing our paper. And we feel badly that Reviewer #1 had such a difficult time with our presentation, although it did not seem to be an issue for the other reviewers. We revised the paper in response to the reviewers’ comments and believe that our revision and the following responses satisfactorily address their concerns. All our additions to the text of the paper are in red type in the pdf markup version of our revised paper.

Responses to the reviewers’ comments below are also in red type.

Reviewer #1: Furano and colleagues present an extension of previous work from their lab (Nabuan Naufer, et al 2016) surrounding the fascinating question of what drove the rapid evolution of L1 ORF1p in early primate evolution. In their previous work, the group observed loss of retrotransposition activity from a chimeric ORF1 comprised of segments from modern (L1HS/PA1) and ancient (PA5) L1s and nicely demonstrated that this loss of activity in the chimera derived from a comprised rate of ORF1p trimerization.

While we appreciate the reviewer’s compliment, the reviewer incorrectly stated the conclusion of our Naufer et al 2016 studies (ref 26). The 151p chimera was normal for trimer-formation. Rather, and in contrast to the retrotransposition-competent modern and ancestral ORF1p trimers (111p and 555p), the retrotransposition-null 151p trimers were unable to rapidly form stably bound oligomers on single-stranded nucleic acid.

In the present manuscript, the group presents a more thorough mutagenesis of ORF1 guided by the changes that occurred between the PA5 and PA1 consensuses. The authors create numerous chimeras and targeted mutants and measure the retrotransposition activity of each. Generally, the data support that there are mutations that have no measurable effect on in vitro retrotransposition, mutations that have moderate effect, mutations that inactivate, and mutations for which their effect of function differs based on the sequence of the rest of ORF1. The logic for why the authors chose the specific mutations they present and any deeper interpretations of interactions amongst mutations is lost in a dense and confusing narrative and presentation of the data. The subsequent analyses of the sequence space of L1 subfamilies was unclearly motivated and hard to follow, and the conclusions made from these data overreached. Overall, the manuscript’s density, jargon, and lack of narrative make it a tough fit for PLOS Genetics. With a clearer motivation of each experiment and a tempering of conclusions, this paper would be a reasonable addition to the literature in a more specialized journal.

We addressed a seminal issue in L1 biology – determing the evolutionary mechanism underlying episodes of extensive amino acid substitutions in the coiled coil ORF1. These events can coincide with the emergence of novel L1 families, most recently 30 – 10 MYA involving the L1Pa7 – L1Pa3 families. Earlier studies by us and others had shown that this episode was compatible with positive selection - more amino acid substitutions than expected by chance. This often implies adaptive evolution, suggestive of an “arms race” between L1 and its mammalian host. Determining its nature would represent a major advance in understanding the interaction between mammals and L1 retrotransposons.

But then we found that coiled coil function is rather impervious to amino acid substitutions (ref 26). With little or no phenotypic effect, and thus not subject to (hidden from) selection, amino acid substitutions could accumulate cryptically. This evolutionary model has dramatically different implications for L1/host interaction than the adaptive model. The alternative model (as elaborated in the 12 cited papers and elsewhere) is embodied by three concepts: (1) “cryptic genetic variation”- defined above; (2) “genetic robustness”, i.e., functional imperviousness to genetic change, which is permissive to cryptic genetic change; (3) “evolvability”, which denotes the cryptic genetic variation that could result from genetic robustness.

(It’s unfortunate that “evolvable” became embedded in this literature because this term is so imprecise – anything that undergoes evolutionary change by definition is evolvable, whether due to cryptic genetic change, positive selection, or genetic drift.)

With respect to L1 coiled coil evolution, and in contrast to what is implied by many of this reviewer’s comments and admonitions, these terms and their associated concepts are not arcane but quite straightforward, based on well understood evolutionary mechanisms.

Here is an example of their use from an important contributor to this field: (ref. 29)

Wagner, A. (2008). Robustness and evolvability: a paradox resolved. Proc. of the Royal Society B: Biological Sciences, 275(1630), 91-100. doi:101098/rspb.2007.1137

“First, multiple experiments show that many organisms harbour cryptic genetic variation‚ reflecting robustness‚ which can become visible in certain environments or genetic backgrounds, and thus lead to enhanced evolvability (Waddington 1953; Dun & Fraser 1959; Waddington 1959; Rutherford & Lindquist 1998; Queitsch et al. 2002).”

In the above quote the phrase “certain...genetic backgrounds” can refer to epistatic mutations that reveal cryptic genetic variation, and which in turn can be beneficial by buffering the effect of randomly occurring negative epistatic mutations. Interplay between epistasis and cryptic genetic change is a major topic in the literature that we cited.

If this model of evolutionary change explains coiled coil amino acid substitutions in the L1Pa7-L1Pa3 families, we might expect three conditions to apply: Genetic robustness (which we established experimentally), permissiveness to cryptic genetic variation, and epistatic interactions within the coiled coil.

Cryptic genetic variation could increase the diversity of coiled coil sequences (i.e., enlarge the coiled coil sequence space), which is what we found by applying principal component analysis (PCA) to determine the sequence space (diversity) of the coiled coils that populated the examined L1 families. We revealed epistasis by characterizing coiled coil function-inactivating or function-restoring mutations in the context of ancestral or modern amino acid residues.

Major Points:

1) The motivation for carrying out each experiment is unstated, and the results section is extremely abrupt in its opening. A clear presentation of the reason and rationale for each experiment would be very helpful.

We added an opening paragraph to the results that provides context for the present experiments, as well as one that summarizes our previous study (26), which revealed the biochemical defect of the inactive coiled coil mutant, 151p. We also modified the Abstract and Discussion to address difficulties the reviewer encountered with our presentation.

2) The authors seem to confound the accepted use of several disinct terms as used in the protein evolution field. First, it has been widely shown in many types of systems that natural proteins tend to be tolerant of most mutations (robustness) but also capable of functional change upon mutation at specific positions which comprise a minority of the sequence (adaptability or evolvability). This robust state of most proteins may result in the accumulation of mutations which are neutral in the context of the present fitness function, but upon some change (environmental, sequence change elsewhere, etc) become advantageous ‚ these now adapive changes were cryptic. The relevance of the concept of cryptic variation to the manuscript is unclear, and the above terms are used inappropriately. For example, (line 134) ‚”Genetic robustness would be permissive to the accumulation of crypt genetic changes - termed ‚evolvability. There are many definitions of evolvability, but evolvability and cryptic variation are not synonymous.

In contrast to what the reviewer asserts, the quoted sentence (line 134) does not equate cryptic genetic change with evolvability. Rather it states that the condition – genetic robustness - is permissive to cryptic genetic change and this permissiveness can be termed “evolvability”.

The relevance of this concept should be much more clearly elaborated.

Here is the original text from which the reviewer excerpted the above quote –now lines 173 – 180 of our revised text.

“Fig 1 shows that while some of the 30 substitutions that differentiate modern and ancestral coiled coils could be considered biochemically “conservative” (*i.e.*, amino acids of similar charge, polarity, hydrophobicity, etc.) others are not. Thus, in terms of the coiled coil-endowed property that renders ORF1p active in retrotransposition, the coiled coil is “genetically robust” *i.e.*, it can be phenotypically indifferent to a considerable degree of genetic change. Genetic robustness would be permissive to the accumulation of cryptic genetic changes - termed “evolvability” (29), which are only manifested by the effect of epistatic substitutions as illustrated by the above examples.

We fail to see any significant contradictions between the meaning of this excerpt and the one that we quoted above from the Wagner paper.

The reviewer also stated:

Further, it seems that (ln 225) ‚ “indifference of L1 retrotransposition activity to extensive ORF1p coiled coil amino acid substitutions”‚ and the (ln 228) ‚”expansion of the functional coiled coil sequence space”‚ are the same.

These two statements do not mean the same thing:

The second statement describes a possible, but not inevitable, outcome of the condition described in the first statement. Determining the nature of this outcome was a major aim of our study.

Protein robustness is the expansion of functional sequence space via indifference to mutation.

We did not make this statement, and it seems a somewhat ambiguous rephrasing of the concepts of genetic robustness and its consequences for genetic change in the coiled coil.

To clarify these statements, it would be useful to reference more of the extensive literature on protein robustness and evolvability and provide a deeper discussion of how these topics relate to the data presented.

This statement expresses assertions repeatedly made by the reviewer, but which are mostly unwarranted as we pointed out above.

3) The finding of context dependence of mutations (epistasis) within ORF1p is not surprising; it likely exists in every protein.

We agree, and it’s a particularly important issue for coiled coils (as we cited, our reference 41 and 42).

It could be interesting to interpret the observed epistasis in the context of the CC structure to try to find mechanistic explanation of the observed effects. Indeed, the manuscript would benefit from a more thorough discussion of how these findings should be interpreted in the context of the ORF1p structure (Khazina, et al. NSMB 2011), and how the present manuscript differs from the mutagenesis data presented in that paper.

All the coiled coil mutations reported in the Khazina 2011 paper were made in multiples of 2 or more, and all except 1 involved highly conserved residues that arose in L1Pa10 or older families, and all involved heptads 9 – 14 (our numbering). The exception is Khazina G132I which is an E in L1Pa5, but its conversion to the modern residue G had no effect on coiled coil function (Fig 1B and S2 Fig2). In contrast, the single epistatic mutations that we documented were in heptads 6, 8 & 9. These arose in the context of other ancestral residues (Fig 5) and their phenotypic effect is profoundly affected by them. Given the ancestral provenance of our mutants and the absence of relevant structural information it is difficult to cogently discuss our single mutations in reference to the Khazina 2011 sets of multiple mutations.

Minor Points:

1) The figure are generally very dense and hard for me to parse. I suggest minimizing the number of colors and sequences to highlight the most important points (eg, a 25 seq alignment (fig 1B) and >20 colors (fig 4))

Arbitrarily truncating our findings would be inappropriate. Each aligned sequence in Fig 2, provides context for the phenotypic effects of introduced coiled coil amino acid substitutions. Each color in Fig 4 corresponds to a distinct subset of coiled coils that contribute to the sequence diversity (sequence space) of the L1Pa7-L1Pa1 coiled coils. The alignment of the consensus sequences of these subsets (Fig 5) provides a comprehensive view of their evolutionary history, which was a main purpose of this study.

2) The construct names are non-intuitive and make any comparison of sequence differences vs activity difference tough. (fig 1)

That is why we had included the column, % ret – for percent retrotransposition activity, to provide these values for each variant in Fig 1C and S3 Fig 3.

3) If the conclusion of the PCA is that ORF1 is robust, it seems that you do not need PCA to observe and quantify variation in this sequence. (ln 155)

We do not understand this sentence, nor what the reviewer means by “ORF1 is robust”. PCA is a standard mathematical technique that we used to visualize the sequence diversity (sequence space) of ORF1 coiled coils during evolution. It cannot be used to determine the biological properties of ORF1.

4) The observation that he paired active and inactive variants share the same sequence space‚ does not demonstrate epistasis (ln 156); it is well- known that single mutations can completely inactivate with little dependence on the rest of the sequence (for example, a highly thermodynamically destabilizing mutation).

Taking the meaning of epistasis in its most general form that the phenotypic effect of a mutation depends on its genetic context (defined similarly by the reviewer in major point 3), then the variant pairs in question are indeed instances of epistasis. R105T in variant 7 is only deleterious in the modern but not ancestral context – in this case an example of negative epistasis*.*

5) The title and introduction needs much less jargon; specifically, the title borrow complex jargon from several fields, making it hard to understand for specialists from many fields.

The reviewer does not cite the jargon, but perhaps “cryptic genetic variation” and “sequence space” are included. The phrase “cryptic genetic variation” has been in the literature since at least 1982 (Ayala, 1982 PNAS:79,550) and a PubMed search for “cryptic genetic variation” returns 20 entries with titles containing this exact phrase and in literature that ranges from plant breeding to immunology. “Cryptic genetic variation” was the title of the most recent review of this topic by Paaby & Rockman in 2014 in Nature Review Genetics, 15, 247. The term “sequence space” is even more common, a PubMed search for titles with “sequence space” and “protein” returned 60 titles. Both phrases concisely embody concepts that would be difficult to describe more explicitly or with fewer words.

Therefore, we retained them in the title, but modified text in the Abstract and elsewhere to describe their derivation (meaning) which we hope will be satisfactory.

6) In the discussion, cis preference is mentioned to support why hybrid trimer formation would not be the driver of having coexisting versions of coil coiled domains within a LINE1 family. Cis preference is known for ORF2p binding to LINE1 mRNA, but this is not necessarily the case for ORF1p?

Cis preference is even more relevant for ORF1p than ORF2p. ORF1p is a non-specific nucleic acid binding protein and required in much larger molar excess over the L1 transcript than ORF2p to generate the L1RNP retrotransposition intermediate. Thus, it is critical to minimize non-productive interactions of ORF1p with non-L1 nucleic acids. Cis preference was experimentally demonstrated for L1 by Wei et al in 2001, the reference to which we noted in our citation to (48).

==================================================================================

Reviewer #2: This work is from a group that in the past has published seminal work on the domain functions of L1 ORF1p and on the evolution of LINE-1 families in both human and non-human genomes. As noted by the authors, variations in the ORF1p coiled-coil domain have previously been interpreted to adaptive evolution by the L1. In this study, Furano and group, expand significantly on the earlier studies to explore in detail the evolutionary dynamics of the coiled-coil domain of ORF1p, relating this to retrotransposition function in an established cell culture reporter assay, and demonstrating a couple of genetic phenomena along the way.

Nevertheless, this study provides no insights into the mechanism effects of the various mutations and expansions of the coiled coil domain of ORF1p, beyond the cell culture assay -- and this assay is a final readout of cumulative mechanisms of retrotransposition and does not itself provide mechanistic insights. Possible experiments might explore effects on chaperone activity, trimerization, or subcellular localization using immunomicroscopy. The authors hint at such experiments in the discussion but do not attempt them.

Apropos of the above statements relative to a mechanistic interpretation of the relationship between coiled coil changes and ORF1p activity – we had already published the biochemical defect in the coiled coil mutant 151p that was correlated with its retrotransposition null phenotype (ref 26).

We now include a paragraph describing these findings. Single molecule nucleic acid binding studies ln collaboration with the Williams laboratory (Northeastern U., ref.26) revealed a novel property of ORF1p - namely that retrotransposition competent ORF1p trimers (111p, 555p) rapidly form stably bound oligomers on long single stranded nucleic acids. In contrast, the 151p trimer cannot form such stably bound oligomers at an appreciable rate. But these studies also showed that 151p, 111p and 555p are essentially indistinguishable by several biophysical parameters (including formation of equally stable trimers) as well as by several standard oligonucleotide-based assays (nucleic acid binding affinity, nucleic acid chaperone activity, stabilization of mismatched oligonucleotide duplexes – data and citations in ref 26).

Single molecule studies are technically demanding and require highly purified nuclease-free ORF1p, which can only be prepared in sufficient quantity after expression in insect cells, a laborious and expensive process. Thus, it would not be feasible to screen the numerous coiled coil variants that we prepared for the current study using single molecule assays. Instead, we used retrotransposition assays as it is the only other assay that distinguished the ORF1p activity of 111p, 555p and 151p. Also, we are now comparing certain pairs of single amino acid substituted coiled coil active/inactive pairs and as mentioned in the Discussion one such pair, m14 vs m15 respectively (Fig 1C), have the same chaperone activity in a FRET assay.

Furthermore, as cited, this group has also previously reported that extensive amino acid substitutions have occurred during evolution of the L1Pa7-L1Pa3 families.

This is so, but as the reviewer’s following comments indicate we now have uncovered a satisfactory evolutionary mechanism for these substitutions, which would ensure L1 survival.

The question is, therefore, is the present detailed study of the ORF1 coiled coil of sufficient interest and scope for the readers of PLoS Genetics. While perhaps somewhat debatable, I would say yes. Using multiple and mutually supporting computational approaches, the study teases out the evolutionary dynamics of an essential cis protein for retrotransposition of the LINE-1, which comprises at least 17 percent of the human genome. The authors clearly show epistasis (eg., the effect of I77L) and genetic robustness of the coiled coil domain. They demonstrate in considerable detail the varied evolutionary history of the CC domain of ORF1, distinct from that of the c-terminal domain of ORF1p and of ORF2p. Phylogenetic and PCA analyses also allowed the authors to identify distinct coiled-coil clusters and to reveal their persistence through L1 family evolution.

We are gratified to read that the description of our findings was clear to the reviewer.

There are a number points that warrant further clarification/elaboration prior to publication:

On line 101 the previously described (ref. 26) "resuscitated" L1Pa5 ORF1p is noted. A sentence or two on how the resuscitation was originally engineered would help reader understanding.

We had described this process in detail previously (ref 26) and now add a brief summary of it in the Materials and Methods.

On line 114 and in Fig. 1, it is stated that ORF1 proteins are expressed at about the same extent in HeLa cells. This is clearly not the case - there would appear to be up to a 3-fold difference, especially obvious in the case of L107F. Properly, band intensities of non-saturating Western blot exposures should be measured, with ORF1p level presented as a ration of tubulin expression. Accurate measurements of expression levels (and also transfection efficiencies) are important in assessing retrotransposition assay results.

We agree with the reviewer’s point and re-worded our Results accordingly. However, the important issue was to determine whether a complete lack of retrotransposition resulted from a lack of ORF1p synthesis and S1 Fig 1 indicates that this was not the case. Interestingly, retrotransposition activities varied between either ~80 – 100% of the control (111p) or almost nil, < 5%. The one exception was the m41b variant which contained a modern amino terminal domain (Fig 1A, NTD) and a fully ancestral coiled coil. We recently found that the reduced retrotransposition activity of m41b reflects cross talk between the NTD and coiled coil and are now examining this in detail. Although this topic is beyond the scope of this paper, it is of interest as the NTD contains highly conserved phosphorylation sites that we earlier showed were essential for retrotransposition.

Regarding transfection efficiency measurements: All of the presented retrotransposition assays represent independent biological replicates and, in each assay, we include as positive and negative controls, 111p and 151p. The retrotransposition values for the 39 independent 111p assays (Fig. 1C) are well within the variance of this assay and control for the transfection efficiency obtainable given the media, transfection reagent, and HeLa cells used for each assay. Also, as evident from Fig 1C most of the critical assays were carried out numerous times (often with different plasmid preparations). We also occasionally carried out **parallel** transfections with βgalactosidase expressing plasmids to also assess the integrity of the transfection reagents, culture media and cells. As we are not making any conclusions based on subtle differences between retrotransposition activity (they are essentially all or none) we are confident in the reliability of the retrotransposition assays.

And finally, over the years we made numerous attempts to measure transfection efficiency in retrotransposition assays, including simultaneous transfection with both the retrotransposition vector and a vector expressing readily assayable products such as βgalactosidase or even a retrotransposition vector with dual reporters. Unfortunately, at least in our hands, unknowable or unforeseen interactions between the promoters on these vectors or their effects on the host often seriously skewed the readouts of the various reporters. Thus, we abandoned these efforts, and rely on the consistency of biological replicates as a measure of the reliability of the retrotransposition assay.

The antibiotic-based retrotransposition assay used here has traditionally been run by first selecting cells on hygromycin for transfection, and then on G418 to select for retrotransposition. The readout is then number of retrotransposition events/number of transfected cells, which controls for variations in transfection efficiencies. This paper failed to select on hygromycin, therefore a discussion of this fact and any possible bias should be included.

As the reviewer states, the original 1996 version of this assay was carried out by first using hygromycin selection, thought to be necessary to enrich for the retrotransposition-vector containing cells. This required an up to 2-week selection for hygromycin resistant cells which were then subjected to an additional 10 - 12 days of selection on G418 to recover the reconstituted neomycin resistance gene, the readout for retrotransposition. It was subsequently found that hygromycin selection was not necessary to obtain valid retrotransposition results thereby reducing the time for each assay from almost a month to little more than 1 week. We therefore deleted the hygromycin gene from our retrotransposition vector.

Clarity of the data would be increased by reproducing the retrotransposition frequency data in an additional column for each of the constructs described in Fig. 1B.

But we did include a column labeled % ret on both Fig 1B and S3 Fig 3 that provides the retrotransposition frequency normalized to that of 111p for each variant.

Line 181: I failed to find Supporting Data/orf1_FL/l1pa5. Is reference being made to a portion of a different file?

We apologize for this oversight on our part. We have now submitted all the relevant ORF1 sequences to the journal as Supporting Data, with our revised paper.

Line 195: I was confused by reference to the "circled node" in Fig. 4. Do you mean the colored circles?

We meant the small filed circle on the node that includes cL2.7, cL2.6, cL1.5 and cL3.4, We changed it to a large grey triangle.

Line 262: the cis-preference model is somewhat misstated, and the sentence should better read: "... whereby retrotransposition-competent L1 proteins bind their encoding transcript". The early experiments on cis- preference indicated that cis-binding was a component of successful retrotransposition, not that L1 proteins could not bind in trans to non-retrotransposition intermediates.

We made the suggested change.

Line 265. It is stated: "Furthermore, there is evidence that translation can be sequestered in subcellular compartments or translation factories (eg. 49)". The relevance of this specifically to the L1 is unclear.

If translation of individual L1 transcripts were compartmentalized, then the issue of synthesizing mixed trimers would be minimized or eliminated. We now explicitly state this in the Discussion.

Methods, line 309. A number of modifications were made to the retrotransposition vector pRTC2, including moving the reporter cassette and swapping in new promoters. Explanations of why these changes were made would be of interest to members of the field.

These changes were made piecemeal over many years based on considerations many of which would not be particularly relevant in the present context. So, I think an expanded discussion of these modifications is not warranted, except for the following: In the original vector the reporter cassette interrupted the 3’ UTR. However, in mammals the 3’ UTR contains a highly conserved motif, a G-tetraplex forming domain (G, Fig 1A), which stimulates retrotransposition (our citations 63 and 64). Therefore, we felt that this region should have been restored to its uninterrupted state.

Reviewer #3: In the manuscript entitle ‚”Cryptic genetic variation enhances primate L1 retrotransposon survival by enlarging the functional coiled coil sequence space of ORF1p” the authors present an original analysis of the human LINE-1 ORF1p coiled coil domain. They used bioinformatic tools to study the expansion of the sequence space of the coiled-coil functional domain.

This manuscript is very well written. The work presented here is of great interest in the field retrotransposon biology, it contributes to our understanding on the variability of the N terminal domain of ORF1p to maintain its activity. It would also contribute to a general understanding on coiled coil domain variability of any protein.

We appreciate the reviewers’ concise and cogent comments on the value of our contribution.

One remaining question could be addressed in more detail in the discussion. This concern line 250 to 255 page 9. Based on the results, the authors suggest that different coiled coil sequences amplified concurrently through several subfamilies. Then, how to explain the presence of several coiled coil domains shared in different subfamilies, since these subfamilies, based on ORF2 phylogeny, appeared sequentially in a unique linear history? It would imply that the coiled coil domain of ORF1p (or the entire ORF1p sequence) can be exchanged between L1 subfamilies.

Not necessarily exchanged, but just continued to be propagated during evolution of the L1 lineage in primates. The distinct L1 families analyzed here, L1Pa7, L1Pa6...L1Pa1 have been classified mainly by characters in the 3’ UTR (personal communication from Arian Smit, developer and maintainer of Repeat Masker and participant in the consortium that developed and maintains the enhanced repeat search tool, DFAM). It is remarkable (also acknowledged by Arian Smit) that evolution of the ORF2 coding sequence tracked fairly well with changes in the 3’ UTR, perhaps reflecting some unrecognized cross talk between these regions of L1. In any event, the most ancestral L1 family that we examined, L1Pa7, contained three seemingly concurrently active coiled coils. As the only evolutionary constraint on the coiled coils is the ability to support retrotransposition despite its amino acid sequence, then there might be no negative constraint on its persistence. And we suggested that this is why the “same” coiled coils are recoverable from distinct families. We changed the Discussion to make these ideas more explicit.

It could also suggest, less likely, convergent mutations of the coiled coil sequence in different subfamilies.

Minor comments

Line 113/114, replace S1 Fig 1 by S2 Fig 2 and vice versa

We thank the reviewer for pointing this out and made these changes.

Line 254, Figs 4 and 5 instead of 5 and 6

Again, we thank the reviewer for pointing this out and made these changes.

In figure 2 and 3, for younger subfamilies L1Pa1 L1Pa2, what is the explanation for the dots that are isolated from the others of the same subfamily?

As can be seen in the coiled coil alignments of L1Pa1 (S7_Fig7_L1Pa1_cc_align.pdf) and L1Pa2 (S8_Fig8_L1Pa2_cc_align.pdf) several coiled coil sequences are fairly divergent from the consensus sequence. These would not map to the same sequence space occupied by most of the coiled coils in the family, indicating the usefulness of PCA to provide a comprehensive view of sequence diversity (space).

Line 181, Supporting Data/orf1_FL was not provided

We apologize for this oversight on our part. We have now submitted all the relevant ORF1 sequences to the journal as Supporting Data, with our revised paper.
